# Tissue Treg Secretomes and Transcription Factors Shared With Stem Cells Contribute to a Treg Niche to Maintain Treg-Ness With 80% Innate Immune Pathways, and Functions of Immunosuppression and Tissue Repair

**DOI:** 10.3389/fimmu.2020.632239

**Published:** 2021-02-05

**Authors:** Ruijing Zhang, Keman Xu, Ying Shao, Yu Sun, Jason Saredy, Elizabeth Cutler, Tian Yao, Ming Liu, Lu Liu, Charles Drummer IV, Yifan Lu, Fatma Saaoud, Dong Ni, Jirong Wang, Yafeng Li, Rongshan Li, Xiaohua Jiang, Hong Wang, Xiaofeng Yang

**Affiliations:** ^1^ Centers for Cardiovascular Research, Lewis Katz School of Medicine at Temple University, Philadelphia, PA, United States; ^2^ Department of Nephrology, The Second Hospital of Shanxi Medical University, Shanxi, China; ^3^ Shanxi Medical University, Shanxi, China; ^4^ Department of Nephrology, The Affiliated People’s Hospital of Shanxi Medical University, Shanxi, China; ^5^ Metabolic Disease Research & Thrombosis Research, Departments of Pharmacology, Microbiology and Immunology, Lewis Katz School of Medicine at Temple University, Philadelphia, PA, United States; ^6^ School of Science and Engineering, Tulane University, New Orleans, LA, United States; ^7^ Inflammation, Translational & Clinical Lung Research, Lewis Katz School of Medicine at Temple University, Philadelphia, PA, United States

**Keywords:** CD4^+^Foxp3^+^ regulatory T cells (Treg), Treg transcription factors (TFs), Treg CD markers, Treg secretome, stem cell secretomes and TFs

## Abstract

We used functional -omics angles and examined transcriptomic heterogeneity in CD4^+^Foxp3^+^ regulatory T cells (Treg) from spleen (s-Treg), lymph nodes (LN-Treg), intestine (int-Treg), and visceral adipose tissue (VAT-Treg), and made significant findings: *1)* Five new shared Treg genes including NIBAN, TNFRSF1b, DUSP4,VAV2, and KLRG1, and 68 new signatures are identified. Among 27 signaling pathways shared in four tissue Treg, 22 pathways are innate immune pathways (81.5%); *2)* s-Treg, LN-Treg, int-Treg, and VAT-Treg have zero, 49, 45, and 116 upregulated pathways, respectively; *3)* 12, 7, and 15 out of 373 CD markers are identified as specific for LN-Treg, int-Treg, and VAT-Treg, respectively, which may initiate innate immune signaling; *4)* 7, 49, 44, and 79 increased cytokines out of 1176 cytokines are identified for four Treg, respectively, suggesting that Treg have much more secretory proteins/cytokines than IL-10, TGF-β, and IL-35; *5)* LN-Treg, int-Treg, and VAT-Treg have 13 additional secretory functions more than s-Treg, found by analyzing 1,706 secretomic genes; *6)* 2, 20, 25, and 43 increased transcription factors (TFs) out of 1,496 TFs are identified four Treg, respectively; *7)* LN-Treg and int-Treg have increased pyroptosis regulators but VAT-Treg have increased apoptosis regulators; *8)* 1, 15, 19, and 31 increased kinases out of 661 kinome are identified for s-Treg, LN-Treg, int-Treg, and VAT-Treg, respectively; *9)* comparing with that of s-Treg, LN-Treg, int-Treg, and VAT-Treg increase activated cluster (clusters 1–3) markers; and decrease resting cluster (clusters 4–6) markers; and *10)* Treg promote tissue repair by sharing secretomes and TFs AHR, ETV5, EGR1, and KLF4 with stem cells, which partially promote upregulation of all the groups of Treg genes. These results suggest that stem cell-shared master genes make tissue Treg as the first T cell type using a Treg niche to maintain their Treg-ness with 80% innate immune pathways, and triple functions of immunosuppression, tissue repair, and homeostasis maintenance. Our results have provided novel insights on the roles of innate immune pathways on Treg heterogeneity and new therapeutic targets for immunosuppression, tissue repair, cardiovascular diseases, chronic kidney disease, autoimmune diseases, transplantation, and cancers.

## Introduction

Our and others’ recent reports showed that cardiovascular (CVD) stressors and risk factors such as hyperlipidemia (1, 2), hyperglycemia (3, 4), hyperhomocysteinemia (5, 6), and chronic kidney disease (7–11), promote atherosclerosis and vascular inflammation *via* several mechanisms. These mechanisms include endothelial cell (EC) activation (1, 12–15) and injury (16); caspase-1/inflammasome activation (7, 9, 16, 17), mitochondrial reactive oxygen species (ROS) (2, 18, 19); Ly6C^high^ mouse monocyte and CD40^+^ human monocyte differentiation (4, 6, 20, 21); impaired vascular repairability of bone marrow-derived progenitor cells (17, 22); downregulated histone modification enzymes (23) and increased histone 3 lysine 14 acetylation (18), and increased expressions of trained immunity pathway enzymes (24, 25). In addition, we also reported that decreased/transdifferentiated CD4^+^Foxp3^+^ regulatory T cells (26–29) (Treg) facilitate vascular inflammation.

Current understanding on T helper cell (Th) differentiation is that in response to stimulation by several different inducing cytokines such as interferon-γ (IFN-γ), interleukin-12 (IL-12), and IL-4, and also anatomical locations (30), naïve CD4+ T cells can be differentiated/polarized into at least nine terminally differentiated Th cell subsets. These subsets include Th1, Th2, Th9, follicular T (Tfh) (30), Tfh-13 (31), Th17, Treg, Th22 (28, 32), Th25 (33), CD4^+^ cytotoxic T cells (CD4+ CTL) (34), tissue-resident memory T cells (Trm), circulating effector memory T cells (Tem), central memory T cells (Tcm) (35), and CD28^null^ T cells (36), suggesting that antigen epitopes-independent innate immune inducing cytokine environments play critical roles for naïve Th0 polarization/differentiation into Treg and other Th subsets. Foxp3 is the major transcription factor (TF), and co-expression of lineage-specifying transcription factors alters the potential function and flexibility of subsets of CD4^+^ T cell; this, in turn, favors the autoimmune pathology (37). Tregs are specialized in the suppression of immuno-pathological reactions in the host immune system against antigens and dangers (28). In addition to inhibition of adaptive immune response, Tregs also play a critical role in controlling various innate immune responses involved in cancers (38), inflammatory diseases including cardiovascular diseases and atherosclerosis (36, 39). Additionally, Tregs play a highly broad spectrum of versatile anti-pathophysiological roles. For example, Tregs facilitate blood flow recovery after ischemia (40), control adipose tissue inflammation, promote muscle repair (41) and maintain tissue/organ homeostasis (42). Tregs’ roles in maintaining self-tolerance and prevention of autoimmune responses and chronic inflammation are mediated by various mechanisms including: *a)* Treg killing of target cells (38); *b)* modulation of target cells *via* cell-cell contact; *c)* inhibition of target cells by exosome-carried microRNAs (28); and *d)* secretion of anti-inflammatory/immunosuppressive cytokines (13) including interleukin-10 (IL-10), IL-35 (1, 43, 44), and transforming growth factor-β (TGF-β). Therefore, cellular therapies using regulatory T (T_reg_) cells are currently undergoing clinical trials for the treatment of autoimmune diseases, transplant rejection and graft-*versus*-host disease (45).

We previously reported that Treg cell death pathways (26–28, 46–52), Treg generated IL-35 (1, 43, 44), and epigenetic pathways (23, 53) may be novel therapeutic targets for maintaining Treg survival, preventing immunosuppressive Tregs from becoming pathological Tregs (28), plastic Tregs and even antigen-presenting Tregs (29), and suppressing inflammation (39). Recently, we proposed a novel concept, which suggests that pathological conditions/environments, *via* antigen epitopes-dependent or independent cellular interactions, re-shape physiological Tregs into pathological Tregs that have weakened immuno-suppressive functions and increased plasticity (28). The following supporting evidence published by other investigators validate our proposed model: *First*, Th1-like Treg phenotype (54, 55), and pro-inflammatory IL-17A cytokine secreting Treg (56); *Second*, immunosuppression-compromised Treg after myocardial infarction (57); *Third*, four different types of “lymphoma Treg” (38); *Fourth*, self-reactive T cells, termed anti-Treg (58); and *Fifth*, FOXO3-expressed in tolerogenic dendritic cells (DCs) in modulating Treg and activating anti-Treg (59). It is accepted that Tregs undergo phenotypic and functional plastic changes into other Th subsets under pathological conditions (32, 60), which are modulated by co-inhibitory receptors (61).

One of Tregs’ functional modes is the secretion of anti-inflammatory/immunosuppressive cytokines (13), including IL-10, IL-35 (1, 43, 44), and TGF-β. However, two important questions remain how many cytokines and secretomes are generated in Treg in various tissues and pathological conditions; and whether those secretomes create a stem cell niche-like microenvironment for Treg maintenance. The secretome, defined as a portion of total proteins secreted by cells to the extracellular space, secures a proper micro-environmental niche, thus maintaining tissue homeostasis (62, 63). Secreted molecules are key mediators in cell-cell interactions, *via* autocrine, and paracrine manners, and influence the cross-talk with the surrounding tissues in addition to their endocrine functions in long-distance by hormones, growth factors, cytokines, adipokines, myokines, cardiokines (64), and chemokines (65). There is strong evidence supporting that crucial cellular functions such as proliferation, differentiation, communication, and migration are regulated strictly by the cell secretome (66). The major differences between our current study and previous reports on the roles of cytokines and chemokines in Treg are that secretome analyses provide a panoramic view on all the secreted genes in Treg, as opposed to focusing on only one or a few cytokines/chemokines (10).

Tregs have functions in various tissue repair (67) including promoting muscle repair (68) and repair after cardiac injury (69), controlling neutrophil recruitment (70), facilitating skin epithelial stem cell differentiation (71) and wound healing (72), enhancing satellite cell expansion in muscle but blocking satellite cell differentiation (73), facilitating lung resolution (74), promoting lung epithelial cell proliferation (75) and lung injury repair *via* generating the growth factor amphiregulin (68), promoting myelin regeneration in central nerve system (76), and protecting kidney injury (77). However, molecular mechanisms underlying Treg promotion of tissue repair remained poorly defined.

In order to broaden our understanding of transcriptomic heterogeneity in Treg from s-Treg, LN-Treg, int-Treg, and VAT-Treg, we hypothesized that tissue Treg heterogeneity could be characterized by examining signature genes, upregulated signal pathways, clusters of differentiation (CD) markers, cytokines and secretomes, TFs, cell death regulatomes, activation and resting status, and Treg similarity of secretomes and TFs to that of stem cells. We conducted comprehensive data analyses on numerous microarray datasets from the NIH-NCBI-GEO database (https://www.ncbi.nlm.nih.gov/gds/). We made the following findings: ***1)*** Five new core Treg genes and 68 new signature genes are identified; ***2)*** s-Treg, LN-Treg, int-Treg, and VAT-Treg have zero, 49, 45, and 116 upregulated pathways, respectively; ***3)*** LN-Treg, int-Treg, and VAT-Treg have 12, 7, and 15 specific CD markers out of 373 CD markers, respectively; ***4)*** analyses of 1,176 cytokines and 1,706 secretomic genes suggest that LN-Treg, int-Treg, and VAT-Treg have 13 functions more than s-Treg; ***5)*** 2, 20, 25, and 43 increased transcription factors (TF) out of 1,496 TFs are identified four Treg, respectively; ***6)*** LN-Treg and int-Treg have increased pyroptosis regulators but VAT-Treg have increased apoptosis regulators, judging by 305 regulatomes in 13 cell death forms; ***7)*** IL-2 receptor β (IL2Rβ) plays an essential role in promoting all tissue Treg shared functions and specific functions; ***8***) LN-Treg, int-Treg, and VAT-Treg increase activated cluster (clusters 1–3) markers; and decrease resting cluster (clusters 4–6) markers; and ***9)*** Four Treg promote tissue repair by generating secretomes similar to that of stem cells; and sharing TFs aryl hydrocarbon receptor (AHR), ETS variant transcription factor 5 (ETV5), early growth response 1 (EGR1), and Kruppel like factor 4 (KLF4) with stem cells. Our results have provided novel insights on tissue Treg heterogeneity and new therapeutic targets for immunosuppression, tissue repair, cardiovascular diseases, chronic kidney disease, autoimmune diseases, transplantation, and cancers.

## Materials and Methods

### Expression Profiles of Splenic Regulatory T Cells, Lymph Nodes Regulatory T Cells, Intestine (Lamina Propria) Regulatory T Cells, and Visceral Adipose Tissue Regulatory T Cells

Microarray datasets were collected from National Institutes of Health (NIH)-National Center for Biotechnology Information (NCBI)-Gene Expression Omnibus (GEO) databases (https://www.ncbi.nlm.nih.gov/gds/) and analyzed with an online software GEO2R (https://www.ncbi.nlm.nih.gov/geo/geo2r/).

### Statistical Analysis of Microarray Data

We applied a statistical method similar to our previously reported meta-analysis (10, 24, 78). We designed a robust housekeeping gene list (**Supplementary Table 1** of housekeeping genes) with help from Eisenberg and Levanon’s (79) excellent work, including ACTB, GAPDH, PGK1, PPIA, B2M, YWHAZ, SDHA, HMBS, and TBP. Briefly, the mean log fold change (LogFC) of housekeeping genes between treatment and control groups vary from −1.27 to 1.28. The target genes with expression changes more than 2-folds were defined as the upregulated genes, while genes with their expression decreased more than 2-folds were defined as downregulated genes |logFC|>1).

### Ingenuity Pathway Analysis

We utilized Ingenuity Pathway Analysis (**IPA**, Qiagen, https://www.qiagenbioinformatics.com/products/ingenuity-pathway-analysis/) to characterize clinical relevance and molecular and cellular functions related to the identified genes in our microarray analysis. Differentially expressed genes were identified and uploaded into IPA for analysis. The core and pathways analysis was used to identify molecular and cellular pathways, as we have previously reported (10, 78, 80).

## Results

1. Transcriptomic differences between Treg and Tconv are small, ranging between 0.29 and 4.28% (1–14.8 folds) among s-Treg, LN-Treg, int-Treg, and VAT-Treg; five new Treg core genes including NIBAN, TNFRSF1b, DUSP4, Vav2, and Klrg1 have been identified; and non-lymphoid tissue niches and lymph node niches contribute more than splenic niches to Treg transcriptomic differences from that of CD4^+^Foxp3^−^ conventional T cell (Tconv).

Our recent report showed that Treg-specific transcription factor (TF) FOXP3 was expressed in trachea, thymus, spleen, mammary gland, lymph node, lung, eye, and blood (29). Additionally, various Treg populations in these tissues have been identified (42). Given Tregs can arise in an antigen epitopes-dependent manner, is this a driving factor in Treg differentiation, or does tissue environment shape Treg transcriptomes? To test this, we collected seven Treg microarray datasets from NIH NCBI-Geo Datasets database (https://www.ncbi.nlm.nih.gov/gds/). These covered four tissue Treg datasets from spleen (SP, s-Treg), lymph nodes (LN, LN-Treg), intestine (small intestinal lamina propria, int-Treg) and visceral adipose tissue (VAT-Treg), one IL-2 receptor β deficient (IL2rβ-/-) Treg, hepatocellular carcinoma Treg, co-stimulatory antibody-treated Treg (**Table 1**). Of note, these datasets were obtained from high-quality experiments since the expression variations of nine housekeeping genes were in limited ranges (**Table 2** and **Supplementary Table 1** of Housekeeping Genes). Surprisingly, when Treg transcriptomes from four tissues were compared to that of Tconv shown in **Table 3**, s-Treg had 31 genes (0.2%) upregulated, 13 genes (0.09%) downregulated from Tconv; LN-Treg had 325 genes (1.5%) upregulated, 72 genes (0.33%) downregulated from Tconv; int-Treg had 371 genes (1.7%) upregulated, 385 genes (1.77%) downregulated from Tconv; and VAT-Treg had 641 genes (2.97%) upregulated, 283 genes (1.31%) downregulated from Tconv. Our results correlated well with a previous report of 200 differentially expressed genes between human type 1 T helper cells (Th1) and and type 2 T helper cells (Th2) (81). These results have demonstrated that *first*, comparing to int-Treg and VAT-Treg, s-Treg and LN-Treg were 10 times less different from that of Tconv in transcriptomes, suggesting that non-lymphoid tissue environments play a significant role in re-shape Treg transcriptomes; *second*, the ratios of upregulated/downregulated genes in LN-Treg and VAT-Treg were a few folds bigger than that in s-Treg and int-Treg, suggesting that functional selection plays critical roles in re-shape the transcriptomes; and *third*, significant expansions of transcriptomic differences in Treg versus Tconv in VAT and intestine in comparison to that Treg in lymphoid tissues such as spleen and lymph nodes indicate that adipose tissues environments and intestine have more types and high strengths of stimuli such as danger/pathogen-associated molecular patterns (DAMPs/PAMPs); and transcriptomic regulatory signals from tissue environment cues are much bigger than that antigen epitopes-dependent Treg differentiation and polarization in healthy conditions.

**Table 1:** Seven microarray datasets were analyzed in our study, which were collected from the NIH GEO Database (https://www.ncbi.nlm.nih.gov/geo/) database and were associated with expression changes of regulatory T cells (Treg) *versus* (*vs.*) conventional T cell (Tconv).

Category	GEO ID	Comparison	Tissue	PMID
Regulatory T cell *vs.* Conventional T cell	GSE119169	FACS-purified CD4+CD25+Foxp3+ Treg cells *vs.* CD4+CD25+Foxp3− Tconv cells	Spleen	30962454
	GSE37532	CD3+CD4+CD25+ Treg cells *vs.* CD3+CD4+CD25- Tconv cells	Lymph node	25550516
	GSE20366	CD4+Foxp3−GFP+ T cells *vs.* CD4+Foxp3−GFP− T cells	Small intestinal lamina propria (int)	25550516
	GSE37532	CD3+CD4+CD25+ Treg cells *vs.* CD3+CD4+CD25− Tconv cells	Visceral adipose tissue(VAT)	20231436
Il2rb−/− Treg *vs.* wt Treg	GSE14350	Il2rb−/− Treg *vs.* wt Treg	Spleen	19185518
Hepatocellular carcinoma	GSE103523	CD14−CD4+CD25high Treg cells *vs.* CD3+CD4+CD25− Tconv cells	Liver	29941600
Co-stimulation	GSE42276	Co-stimulatory antibody treated Treg cells *vs.* untreated Treg cells	Spleen and lymph node	23674602

**Table 2:** The expression changes of nine widely used housekeeping genes were limited, which showed the high quality of seven datasets we studied.

Gene symbol	GSE119169 SP		GSE37532 LN		GSE20366 int		GSE37532 VAT		GSE14350 spleen		GSE103523 Liver	
	p value	logFC	p value	logFC	P value	logFC	p value	logFC	p value	logF0C	p value	Log fc
ACTB	0.05	0.09	0.79	0.03	0.56	0.41	0.77	−0.04	0.04	−0.18	0.04	0.89
GAPDH	0.00	0.18	0.19	−0.15	0.86	−0.16	0.74	0.04	0.18	0.14	0.17	0.68
PGK1	0.41	−0.04	0.27	0.12	0.60	0.09	0.13	0.21	0.24	0.06	0.67	−0.22
RPLP0	0.26	0.06	0.01	−0.37	0.70	−0.28	0.14	−0.19	0.97	0.01	0.10	−0.98
B2M	0.28	0.06	0.17	0.20	0.29	0.19	0.50	0.08	0.63	−0.04	0.04	0.20
YWHAZ	0.05	−0.13	0.00	0.45	0.09	0.61	0.37	0.12	0.24	−0.61	0.74	−0.10
GUSB	0.11	−0.10	0.11	0.19	0.51	−0.40	0.76	0.04	0.05	0.62	0.00	0.37
HMBS	0.38	−0.04	0.31	0.13	0.20	−0.61	0.33	−0.16	0.17	0.26	0.53	−0.04
TBP	0.16	0.08	0.49	−0.08	0.62	0.09	0.84	−0.03	0.17	0.28	0.28	0.17

Expression changes of housekeeping genes in the dataset GSE42276 were listed in **Supplementary Table 1**.

**Table 3:** Overall changes in the comparison of regulatory T cells (Treg) *versus* conventional T cells (Tconv) in different tissues showed that Treg can be modulated more significantly by the microenvironment in different peripheral tissues than Tconv, by which we can defined the tiers of the tissues.

Tissue	SP	LN	int	VAT
GEO ID	GSE119169	GSE37532	GSE20366	GSE37532
Up	31 (0.2%)	325 (1.5%)	371 (1.7%)	641 (2.97%)
Down	13 (0.09%)	72 (0.33%)	385 (1.77%)	283 (1.31%)
Total Changed	44 (0.29%)	397 (1.84%)	756 (3.48%)	924 (4.28%)
Total	15,281	21,611	21,720	21,611

Cutoff: p value < 0.05, |logFC| > 1); spleen is defined as the first tier and lymph node as the second; int and VAT as the third.

**Figure 1:**
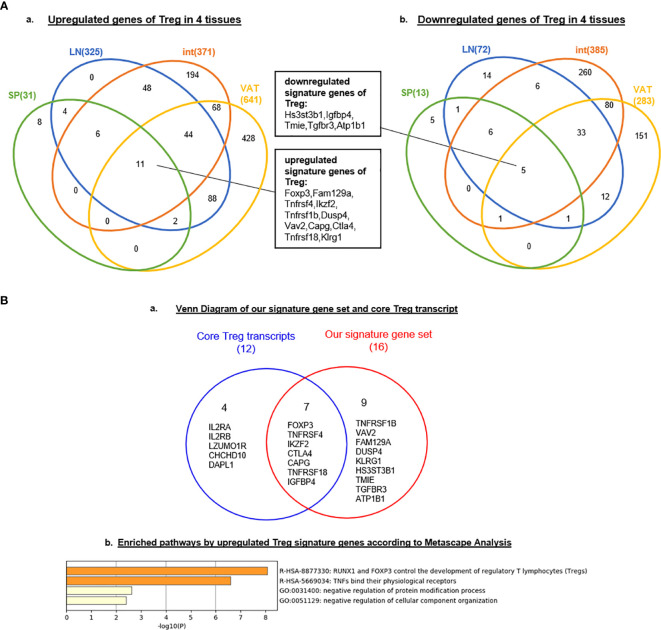
**(A)** Nine new Treg signature genes are identified. Venn Diagram showed the signature genes of Treg commonly upregulated (left) and downregulated (rignt) in four tissues we studied, indicating the same source or progenitor of Treg in different tissues. These shared genes could be identified as signature genes of regulatory T cell. **(B)** RUNX1 and Foxp3 regulate tumor necrosis factors (TNFs) and their receptors. We compared 16 signature genes (11 upregulated genes and 5 downregulated genes in Figure 1) with core Treg transcripts in spleen identified by single-cell RNA-seq (PMID: 29434354). Nine genes of our signature gene set were newly identified according to our data; Enriched pathways active by upregulated Treg signature genes we generated according to Metascape (http://metascape.org/gp/index.html#/main/step1) indicated RUNX1 and FOXP3 could control development by these signature genes and this gene set could also regulate the binding of TNFs and their receptors; meanwhile, Treg could negatively regulated the protein modification and cellular component organization.

Tissue diversification of Treg transcriptomic differentiation from Tconv emphasized the tissue environmental effects and signals on transcriptomic remodeling. We hypothesized that regardless of tissue re-modeling, Treg from four different tissues share Treg signature genes in addition to the differences. To test this hypothesis, we used the Venn Diagram to analyze Treg genes from four tissues. As shown in **Figure 1A-A**, the results on Treg upregulated genes showed that: four tissue Treg shared 11 upregulated genes including Foxp3 (38, 51), Fam129a (NIBAN, Niban apoptosis regulator 1) (82), tumor necrosis factor receptor (TNFR) superfamily member 4 (TNFRSF4, CD134, OX40) (83, 84), IKAROS family zinc finger 2 (Ikzf2) (85), TNFR type II (TNFRSF1b, CD120b) (86), Dual Specificity Phosphatase 4 (DUSP4) (87), Vav guanine nucleotide exchange factor 2 (Vav2) (88), capping actin protein, gelsolin like (Capg) (84), Ctla4 (84), TNFR superfamily member 18 (TNFRSF18, GITR, CD357) (84) and killer cell lectin like receptor G1 (Klrg1) (89); and four tissue Treg shared five downregulated genes (**Figures 1A, B**) such as heparan sulfate-glucosamine 3-sulfotransferase 3B1 (Hs3st3b1), insulin like growth factor binding protein 4 (Igfbp4), transmembrane inner ear expressed protein (Tmie), transforming growth factor Beta receptor 3 (Tgfbr3), and ATPase Na+/K+ transporting subunit Beta 1 (Atp1b1). Of note, it has been reported that nine cytokines and 88 transcription factors are differentially expressed among tissue Treg but not shared genes (90). Comparing with the 12 core Treg genes expressed in all Treg regardless of their areas recently reported using single cell RNA-Seq (84) including Foxp3, Il2ra, Il2rb, Tnfrsf4, Ikzf2, Ctla4, Capg, Izumotr, Chchd10, Tnfrsf18, Dapl1, and Igfbp4, our results in **Figure 1B-A** and **Figure 3A-A** have identified five new upregulated Treg core genes regardless of tissues including Fam129a (NIBAN, regulating p53-mediated apoptosis), TNFRSF1b (CD120b, mediating metabolic effects of TNFa), DUSP4 (dual specificity phosphatase 4, dephosphorylating mitogen-activated protein kinases (MAPKs) extracellular signal-regulated kinases 1/2, ERK1/2), Vav2, EFNA1-induced RAC1 GTPase activation and vascular endothelial cell migration and assembly), and Klrg1 [killer cell lectin like receptor G1, playing an inhibitory role on natural killer (NK) cells and T-cell functions upon binding to their non-MHC ligands] and five downregulated genes Hs3stb1, Tmie, Tgfbr3, Igfbp4 and Atp1b1 (**Figure 1A-B**).

To further determine the signaling pathways that our 16 Treg signature genes (**Figure 1B**), we used the Metascape database (http://metascape.org/gp/index.html#/main/step1http://metascape.org/) that is suitable for analyzing small numbers of genes in comparison to that of Ingenuity Pathway Analysis (IPA) database. As shown in **Figure 1B-B**, two new Treg signaling pathways were *i)* RUNX1 and Foxp3 control Treg development (49), and *ii)* TNFs bind their physiological receptors. Two additional pathways included: *1)* negative regulation of protein modification process and *2)* negative regulation of cellular component organization. Of note, a recent report identified a new all-trans retinoic acid-induced CD161+ Treg in the intestine, which have a transcription network including BTB Domain And CNC Homolog 2 (BACH2), RAR related orphan receptor C (RORγT), FOS like 2, AP-1 transcription factor subunit (FOSL2), AP-1 (c-Jun and c-Fos), and RUNX Family Transcription Factor 1 (RUNX1) (91). It was reported that RUNX1 is required for the optimal regulation of Foxp3 expression in human T cells (92); and differentiating Treg will have recognized their cognate antigens and received T cell antigen receptor (TCR) signals before initiating Foxp3 transcription, which is triggered by TCR-induced transcription factors including Nuclear Factor Of Activated T Cells 2 (NFAT2), AP-1 (Jun and Fos) and NF-κB. Once expressed, Foxp3 seizes TCR signal-induced transcriptional and epigenetic mechanisms through interacting with AML1/Runx1 and NFAT (93). Thus, Foxp3 modifies gene expression dynamics of TCR-induced genes, which constitute cardinal mechanisms for Treg-mediated immune suppression. Our results have demonstrated that RUNX1 may also be a shared Treg transcription factor. Taken together, our results demonstrated that transcriptomic differences between Treg and Tconv are small, ranging between 0.29 and 4.28% (1–14.8 folds) among s-Treg, LN-Treg, int-Treg and VAT-Treg; five new Treg core genes including NIBAN, TNFRSF1b, DUSP4, Vav2, and Klrg1 have been identified; and non-lymphoid tissue niches and lymph node niches contribute more than splenic niches to Treg transcriptomic differences from that of CD4^+^Foxp3^-^ conventional T cell (Tconv).

2. A list of 68 new Treg signature genes have been generated from three partially overlapped gene groups; and among 27 tissue Treg shared pathways, 22 pathways (81.5%) are inante immune pathways.

**Figure 2:**
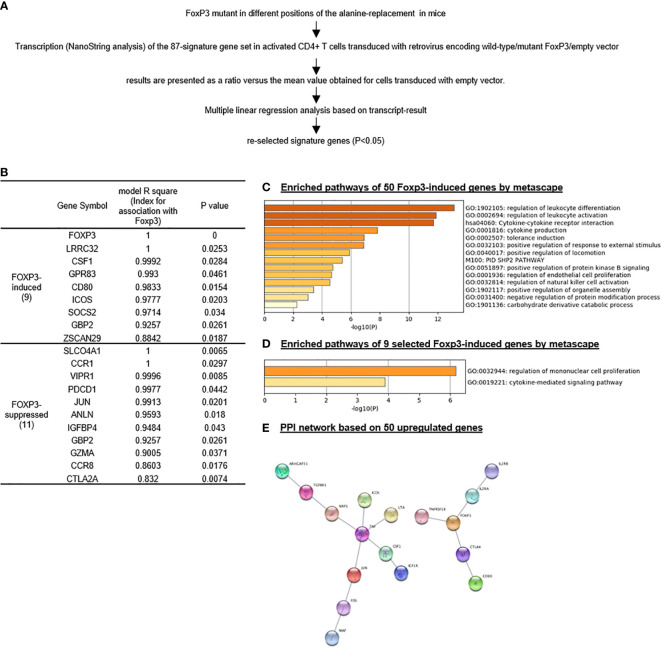
Foxp3 reliably promotes “monocyte” proliferation signaling and suppresses MAPK signaling in Treg; and nine out of 50 Foxp3-induced genes are identified as most reliable Foxp3-induced genes. We re-analyzed the Treg signature gene set, which was tested by Nanostring using Treg extracted from spleen on Foxp3 mutant mice (PMID: 28892470) and re-selected Treg signature gene set based on our re-analysis. **(A)** flow chart of Treg signature gene set re-selection by multiple linear regression based on their expression levels according to Nanostring. **(B)** To identify most closely related genes with FOXP3, we identified only 20 out of total 87 (50 Foxp3-induced and 37 Foxp3-suppressed genes) signature genes whose expression is consistent with that of Foxp3 by statistic analysis based on the expression result of Nanostring in this paper, either positively or negatively; **(C–E)** Enriched pathways by the original gene set and selected gene set according ot metascape analysis showed great difference, indicating other mechanism along with Foxp3 modulated Treg homeostasis and shape Treg signature. Protein-Protein Interaction (PPI) by App String in Cytoscape Platform identified that Tnf and Foxp3 were two central molecules of 50 upregulated signature genes; interaction of chemokine and chemokine receptors was one of the basic network in 37 downregulated signature genes. (Original results for Nanostring was downloaded from **Supplementary Table 4** (https://www-nature-com.libproxy.temple.edu/articles/ni.3835#Sec27.)

We then hypothesized that Treg signature genes are induced by Treg specific transcription factor Foxp3. An excellent report identified 50 genes induced by Foxp3 and 37 genes suppressed by Foxp3 (94). Based on the expression levels based on the results from Nanostring of the 50 Foxp3-induced Treg genes and 37 Foxp3-suppressed Treg genes by a series of wild-type Foxp3 and Foxp3 mutants, we performed multiple linear regression analyses to determine the genes statistically significantly induced by Foxp3 and suppressed by Foxp3 (**Figure 2A**). The original Nanostring data were obtained from the **Supplementary Table 4** of a paper from Dr. Benoist’s team (94). As shown in **Figure 2B**, nine out of reported 50 Foxp3-induced genes, and 11 out of reported 37 Foxp3-suppressed genes were identified with statistical significance (p < 0.05). The nine statistically significant Foxp3-induced genes were newly related to Foxp3 function.

As shown in **Figure 2C**, the Metascape analysis identified 14 signaling pathways out of the reported 50 Foxp3-induced genes. As shown in **Figure 2D**, after our statistical re-selection, the Metascape analysis only identified two pathways; regulation of mononuclear cell proliferation and cytokine mediated signaling pathway. As shown in **Figure 2E**, using protein-protein interaction (PPI) network database (https://www.sciencedirect.com/topics/medicine-and-dentistry/protein-protein-interaction), we found two hubs when using the original 50 reported Foxp3-induced genes, one TNF centered, one Foxp3 centered. Reanalylsis with the nine significant genes out of 50 resulted in only the Foxp3 hub. These results demonstrated that our re-selection of statistically significant Foxp3-induced genes was appropriate to zoom in on the Foxp3 regulated core genes.

**Figure 3:**
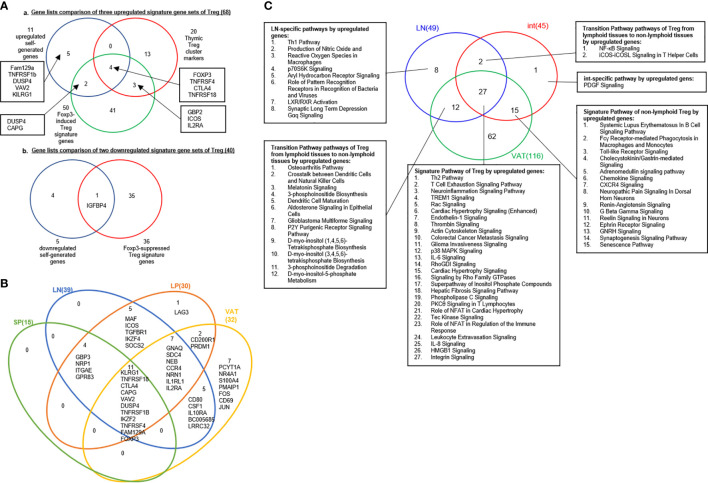
**(A)** A new 68 upregulated signature genes are identified in Treg: 11 upregulated genes in our finding, 20 thymic Treg cluster markers were identified based on single cell-sequence by S A Teichmann (PMID: 32079746) and 50 Foxp3-induced Treg signature genes (PMID: 28892470). Venn diagram showed that FOXP3, TNFRSF4, CTLA4, TNFRSF18 were common signature genes which could be upregulated in Treg; IGFBP4 was shared by downregulated gene set we generated and 36 Foxp3-suppressed Treg signature genes. **(B)** Venn Diagram showed shared consistent upregulated signature genes in four tissues we investigated in our study. **(C)** Twenty-two out of 27 pathways, shared by upregulated genes of Treg in LN, int and VAT are innate immune pathways as signature pathways of Treg. 15 pathways shared by int and VAT could be identified as peripheral Treg; two pathways shared by LN and int and 12 pathways shared by LN and VAT were classified as transition pathways from lymphoid tissues to non-lymphoid tissues. See the detailed data in **Supplementary Figures 2** and **3**.

A recent single-cell RNA-Seq analysis reported the identification of 20 Treg core genes (95). To consolidate Treg signature genes, we collected 11 Treg genes shared in four tissues, 50 reported Foxp3-induced genes, and 20 thymic Treg cluster genes and performed the Venn Diagram analysis as shown in **Figures 3A**, **B**. The results identified 68 upregulated Treg genes and 40 downregulated genes (**Table 4**). When those upregulated Treg signature genes were searched in tissue Treg transcriptomic data, 15 out of 68, 39 out of 68, 30 out of 68, and 32 out of 68 upregulated Treg signature genes were upregulated in s-Treg, LN-Treg, int-Treg and VAT-Treg, respectively. We also found 2, and 5 out of 68 upregulated Treg signature genes were downregulated in int-Treg and VAT-Treg, respectively. Similarly, we found that 0 out of 40, 4 out of 40, 2 out of 40, and 12 out of 40 downregulated Treg signature genes were upregulated in s-Treg, LN-Treg, int-Treg, and VAT-Treg, respectively. We also found 5 out of 40, 9 out of 40, 15 out of 40, and 13 out of 40 downregulated Treg signature genes were downregulated in s-Treg, LN-Treg, int-Treg, and VAT-Treg, respectively (**Table 5**). To determine the distribution of 68 upregulated Treg signature genes in Treg in four tissues, we performed the Venn Diagram analysis. As shown in **Figure 3B**, the results showed that 1) s-Treg shared the same 15 upregulated Treg signature genes with LN-Treg and int-Treg; 2) s-Treg shared 11 upregulated Treg signature with LN-Treg, int-Treg, and VAT-Treg; 3) LN-Treg shared 27 and 23 upregulated Treg signature genes with int-Treg and VAT-Treg, respectively; and 4) int-Treg shared 20 upregulated Treg signature genes with VAT-Treg.

**Table 4:** Based on all gene sets analyzed above, we generated a list of Treg signature genes including 68 upregulated and 40 downregulated genes in **Figure 4**.

Category	Gene symbol
**Upregulated signature** **(** 66)	FOXP3,FAM129A,TNFRSF4, TNFRSF1B,DUSP4,VAV2,CAPG,CTLA4,TNFRSF18,KLRG1,ITGAE,NRN1,NRP1,GPR83, NEB,IL2RA,GBP3,CSF1,GNAQ,SDC4,IL1RL1,CCR4,ICOS,IL10RA,NT5E,PGLYRP1,LAG3,PRDM1,TGFBR1,MAF,NR4A1, CD200R1,LRRC32,SOCS2,IKZF4,IL2RB,BC005685,IL6RA,CD80,ZSCAN29,TGM2,P2RY10,IGF1R,JUN,LTA,CD69,FOS,PCYT1A, ARHGAP31,TNF,RORC,GBP2,FOLR4,IFGGA2,IL32,PIM2,S100A4,BATF,CARD16,TIGIT,PMAIP1,MIR4435-2HG,CD7,LINC00152,AC133644.2,RTKN2,EPSTI1,IKZF2
**Downregulated signature** **(** 39)	HS3ST3B1,IGFBP4,TMIE,TGFBR3,ATP1B1,VIPR1,FGL2,PDE3B,CXCR6,GZMB,METTL9,LGALS3,PDCD1,TBX21,ID2,IL2,CCR8, IL12RB2,XCL1,FOSL2,LGALS1,EGR1,IL4,POLE,CTLA2A,SLCO4A1,CCR1,LDHA,SLC16A3,CXCR4,IL5,ANLN,CD226,GEN1,EZH2,GZMA,EOMES,IL18RAP,PTPN22,TXN1

**Table 5:** Comparison of signature genes in four tissues in our study showed that with the change of different tissue tier, inconsistent percentages of Treg signature genes (upregulated signature gen gets downregulated or *vice versa*) were increased, indicating Treg plasticity in tissues, especially in non-lymphoid tissues.

		SP	LN	int	VAT
Upregulated signature (66)	Up	15	39	30	32
	Down	0	0	2	5
	Total	15	39	33	37
Downregulated signature (39)	Up	0	4	2	12
	Down	5	9	15	13
	Total	5	13	17	25
Consistent signature		20	48	45	45
Inconsistent signature		0	4	4	17
Inconsistent percentage		0	8.33%	8.89%	37.78%

This result also proved the reliability of the signature gene set we generated.

Our results have demonstrated that *i)* These 11 shared Treg signature genes were the same as 11 genes shared by Treg from four tissues shown in **Figure 1A-A**, suggesting that the 11 genes are the Treg essential suppressive function-required regardless of tissue environments; *ii)* nine statistically significant Foxp3-induced genes are different from these 11 core Treg signature genes except Foxp3, suggesting that nine out of 11 core Treg signature genes are not induced by Foxp3 but contribute to Treg suppressive function; *iii)* 20 thymic Treg cluster genes share only four genes with 11 Treg signature genes shared by Treg from four peripheral tissues and reported 50 Foxp3-induced genes such as Foxp3, TNFRSF4 (CD134, OX40), CTLA4 and TNFRSF18 (GITR, CD357); *iv)* seven out of 11 Treg core genes shared by Treg from four tissues and 46 out of reported 50 Foxp3-induced genes do not share with 20 thymic Treg cluster genes, suggesting that those 53 genes (93%) out of 57 genes (**Figure 3A-A**) are the peripheral Treg genes expressed from Foxp3 induction in peripheral tissues or signaling pathways from peripheral tissue environments but not expressed in thymic Treg; and *v)* 16 out of 20 (80%) thymic Treg cluster genes are not shared with tissue Treg, suggesting that thymic Treg lose 80% of thymic cluster genes when they adapt tissue environments.

We hypothesized that tissue Treg have their own signaling pathways associated with their upregulated genes and downregulated genes. To test this hypothesis, we performed IPA with the tissue Treg genes shown in **Table 3**. IPA did not yield any significant pathways since s-Treg had 31 genes upregulated and 13 downregulated in comparison to that of Tconv (**Table 3**). As shown in **Table 6** and **Supplementary Figure 1**, the results showed LN-Treg had 47 significant pathways upregulated and one pathway downregulated; int-Treg had 45 pathways upregulated and 63 pathways downregulated; and VAT-Treg had 116 pathways upregulated and 48 pathways downregulated. As shown in **Figure 3C**, the results showed that *1)* LN-Treg shared upregulated two pathways with int-Treg in T Helper Cells, and shared 12 upregulated pathways with VAT-Treg. Of note, these 14 pathways indicated lymphoid Treg to non-lymphoid Treg transition; *2)* int-Treg shared 15 upregulated pathways with VAT-Treg, which indicated non-lymphoid Treg shared pathways; *3)* three tissue Treg shared 27 upregulated pathways; and 4) based on activation z scores, we compiled all the top signaling pathways from LN-Treg, int-Treg, and VAT-Treg in **Figure 3C**. We found that the top 30 pathways in **Figure 3C** overlapped with the 27 Treg shared pathways in **Figure 3C**.The differences between those two lists included the following few pathways: Glioma Invasiveness Signaling was on the 27 shared pathways whereas four pathways on the 30 top pathways but not on the 27 Treg shared pathways included dendritic cell maturation, osteoarthritis pathway, NF-kB signaling, and B cell receptor signaling.

**Table 6:** Ingenuity Pathway Analysis (IPA) also showed that the expression changes of upregulated, and downregulated genes of Treg *vs.* Tconv from different tiers of four tissues were involved in canonical pathways to maintain their roles of homeostasis.

Active pathways of Treg in four tissues				
Tissue	SP	LN	int	VAT
GEO ID	GSE119169	GSE37532	GSE20366	GSE37532
Up	0	49	45	116
Down	0	1	63	48

In addition, the results of active pathways also confirmed the tiers of these four tissues we defined. Cutoff: z-score >2) The details of pathways were shown in **Supplementary Figure 1**.

**Figure 4:**
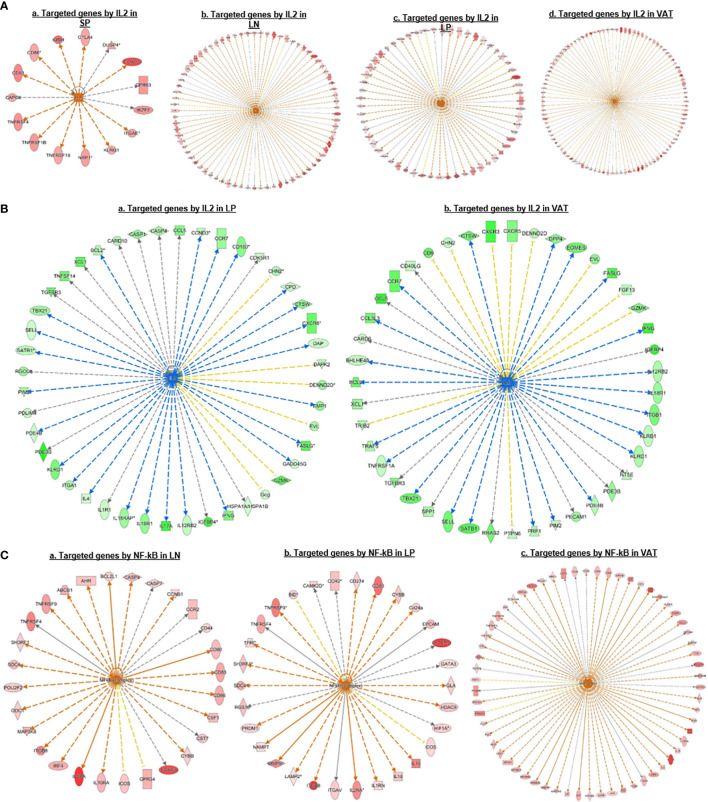
**(A)** IPA upstream regulator predictions of *upregulated* genes in Treg suggest that IL 2 is a universal regulator of Treg in different tissues; and its function could be amplificated from lymphoid tissues to non-lymphoid tissues. We also found that many signature genes we identified were targeted by IL2, such as CTLA4, FOXP3, IKZF2, KLRG1, TNFRSF18, TNFRSF4, etc. **(B)** IPA Upstream regulator predictions of *downregulated* genes also suggest that IL 2 is a universal regulator of Treg in non-lymphoid tissues but not in the lymphoid tissues. **(C)** IPA Upstream regulator predictions of *upregulated* genes suggesting that NF-kB is a universal regulator of Treg in different tissues except spleen. We found that upregulated signature genes such as FOXP3, IL2RA, TNFRSF4, and IRF4 were parts of NFkB targets, indicating the important role of NFkB in Treg homeostasis.

We then hypothesized that Treg from various tissues have shared upstream master regulators. To test this hypothesis, we performed IPA. As shown in **Figure 4A**, the results showed that Treg upregulated genes in each tissue can all be targeted by upstream regulator IL-2. In addition, Treg downregulated genes in the intestine and VAT can also be targeted by IL-2 (**Figure 4B**). Of note, IL-2 as the common upstream regulator was not on the IPA pathways on the 27 shared Treg pathways in **Figure 3C**, suggesting the IPA pathways in both tissue Treg specific pathways and shared Treg pathways are mostly downstream signaling pathways rather than the upstream regulator(s). Moreover, the results in **Figure 4C** showed that upregulated genes in Treg from LN, intestine and VAT but not spleen can all be targeted by universal upstream regulator NF-kB, which were well correlated with the top transition pathways from lymphoid Treg to non-lymphoid Treg that LN-Treg shared with int-Treg and VAT-Treg in **Figure 3C**. From the top pathway comparison data from four tissue Treg, NF-KB *z* scores were high in LN-Treg and int-Treg but lower in VAT-Treg.

Taken together, these results have demonstrated that *first*, the 27 shared signaling pathways are the key signaling pathways for Treg suppressive functions regardless of tissue differences; *second*, LN-Treg have eight specific signaling pathways; int-Treg have only one specific pathway; *third*, the 14 shared pathways between LN-Treg and int-Treg and LN-Treg and VAT-Treg focused on NF-kB, ICOS co-stimulation, DC maturation, and metabolisms; *fourth*, the pathways shared by int-Treg and VAT-Treg focused on autoimmune lupus, phagocytosis in macrophages and monocytes, neuroendocrine signaling, cytokine/chemokine and hormone secretion and senescence; *fifth*, all three tissue Treg shared pathways cover a broad spectrum of functions. The results have clearly demonstrated that Treg are functional not only in suppressing immune and autoimmune responses, inflammations, but also promoting muscle repairing, tissue regeneration, and tissue homeostasis, etc; and *sixth*, IL-2 and NF-KB were the universal upstream regulators for tissue Treg. Our previous report showed that in addition to kinase-phosphatase regulatory mode, NF-KB canonical and non-canonical pathways can be regulated in pre-translational mode (96). Our findings here are correlated well with recent reports that NF-kB canonical pathway components c-Rel is critical for thymic Treg development while p65 is essential for mature Treg identity and maintenance of immune tolerance by promoting the formation of a Foxp3-specific enhanceosome (97, 98); and NF-kB alternative (non-canonical) pathway components p100 (nfkb2) is essential for Treg suppressive function and inhibition of RelB (99–101).

3. S-Treg have eight CD markers but no specific ones; 12-, 7-, and 15- CDs have been identified as tissue-specific effector Treg markers for LN-, intestine-, and VAT-Treg, respectively; interactions between CDs and their receptors mediate tissue Treg signaling; and S-Treg are the most naïve peripheral lymphoid Treg.

**Figure 5:**
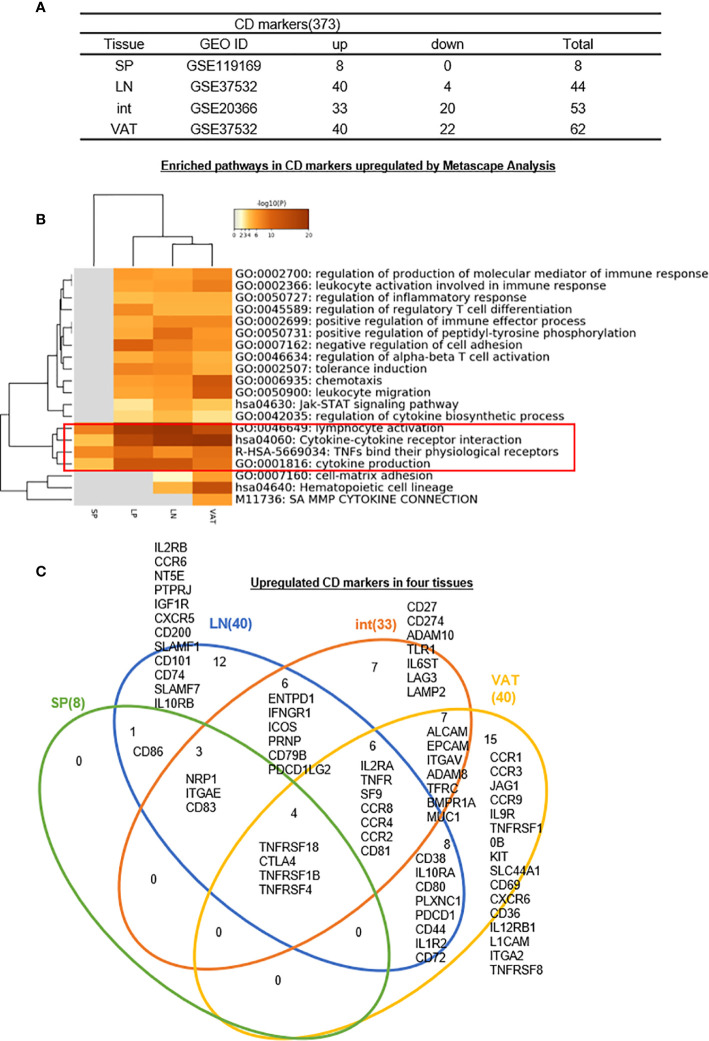
**(A–C)** Twelve lymph nodes Treg specific CD makers, seven intestine Treg-specific CD makers, and 15 VAT Treg-specific CD makers are identified, which mediate 20 immune signaling pathways after screening for total 373 CD markers (https://www.proteinatlas.org/search/protein_class:CD+markers) shared (logFC) in four tissues we studied indicating modulation and plasticity of Treg in tissues and Metascape analysis showed that cytokine-cytokine receptor interaction, TNFs binding their physiological receptors and cytokine production were all active by upregulated CD markers, showing important function of cytokines and TNF receptors of Treg; Immunoregulatory interactions between a lymphoid and a non-lymphoid cell was downregulated in Treg. See the detailed data in **Supplementary Figure 4**.

Identification of clusters of differentiation (CDs) using monoclonal antibodies has revolutionized immunology. Originally CD4+CD25+ or CD4+CD25^high^ was the markers for characterization of Treg (26, 27, 102), later on Treg specific transcription factor Foxp3 was introduced as a reliable marker for Treg (29, 49, 51, 53). As we recently reviewed, at least six Treg subsets can be identified including: *i)* Fxop3^+^CD28^+^GranzymeB^+^Helios^+^TGFβ-insensitive thymic (tTreg), *ii)* CD62L^high^CCR7 (CD197)^+^ or CD45RA^high^CD25^low^ central Treg; *iii)* Foxp3^+^CTLA4^+^IL-10^+^TGFβ-sensitive inducible Treg (iTreg)/peripheral Treg (pTreg), *iv)* IL-35 secreting Treg (iTreg35), *v)* CD62L^low^CCR7^low^CD44^high^KLRG^+^CD103^+^ or CD45RA^low^CD25^high^ effector Treg, and *vi)* Foxp3^+^other transcription factor+ resident Treg (28). Of note, Helios (transcription factor), TGFb (cytokine), IL-10 (cytokine), granzyme B (multiple cellular locations), and IL-35 (cytokine) are not cell surface CD markers, which make flow cytometry challenging since the intracellular staining of monoclonal antibodies with a Golgi blocker is needed (https://www.bdbiosciences.com/ds/pm/tds/555029.pdf). Therefore, the characterization of novel CD markers for Treg will significantly advance our understanding on Treg function and homeostasis. We hypothesized that transcriptomic analysis of CD marker expression of Treg facilitate the identification of new CD markers for tissue Treg. We collected a complete list of 373 CDs from a comprehensive protein database (https://www.proteinatlas.org/search/protein_class:CD+markers). As shown in **Figure 5A** in comparison to the counterpart Tconv, s-Treg upregulated 8 CDs; LN-Treg upregulated 40 CDs, and downregulated 4 CDs; intestine (LP) Treg upregulated 33 CDs and downregulated 20 CDs; and VAT-Treg upregulated 40 CDs and downregulated 22 CDs.

We hypothesized that Treg from four tissues have shared and specific CD markers. We performed the Venn Diagram analysis to test this hypothesis. As shown in **Figure 5C**, the results showed that *i)* s-Treg shared total eight CDs with LN-Treg. S-Treg shared seven of the eight CD markers with int-Treg except CD86, which may be the naïve-like central Treg (103) markers; *ii)* LN-Treg had 12 specific CD markers, suggesting that these 12 CD markers were the lymphoid effector Treg markers; *iii)* LN-Treg shared 19 CD markers with int-Treg; *iv)* LN-Treg shared 18 CD markers with VAT-Treg. Of note, among 17 CD markers shared, first 10 CD markers also shared with LN-Treg. The last seven markers were non-lymphoid effector Treg-specific CD markers (103) including ALCAM (CD166), EPCAM (CD326), ITGAV (CD51), ADAM8 (CD156), TFRC (CD71), BMPR1A (CD292), and MUC1 (CD227); *vii)* VAT-Treg had 15 specific CD markers; and *viii)* all four tissue Treg shared four CD markers.

As shown in **Figure 5B**, the Metascape pathway analysis identified 20 pathways using upregulated CDs from four tissue Treg, which are all related to innate and adaptive immune regulations. Among 20 pathways, four pathways were shared in all four tissue Treg even including s-Treg including *1)* lymphocyte activation, *2)* cytokine-cytokine receptor interaction, *3)* TNFs bind their physiological receptors, and *4)* cytokine production. Of note, these four pathways were addition to the 27 shared Treg pathways identified from LN-Treg, int-Treg and VAT-Treg in **Figure 3C**.

Taken together, our results have demonstrated that *first*, since LN-Treg (40 upregulated CD markers) have five folds more CD markers than s-Treg (8 upregulated CD markers), suggesting that LN-Treg are more likely to be effector Treg; and s-Treg are more likely to be naïve-like central Treg (28, 103), which may just be matured from thymic Treg in peripheral lymphoid tissue. Spleen is a peripheral tissue of the circulatory system embedded with multiple LN-like structures (White pulp, WP), which functions similarly to how LNs drain and monitor antigens from tissues. In the WP, naïve and central memory T cells are activated in response to cognate antigens. CCR7 is required for T cell concentration in the T cell zone in the WP (104). However, our results in **Figure 5C** showed that s-Treg have no increased CCR7 levels over Tconv, resulting in a scattering of relatively naïve Treg throughout the spleen. *Second*, those seven CD markers shared between three Treg such as s-Treg, LN-Treg, and int-Treg are more likely to be central Treg CD markers shared by lymphoid and non-lymphoid tissue Treg; *Third*, CD86 shared by s-Treg and LN-Treg is the only central lymphoid Treg marker; *Fourth*, LN-Treg have 12 specific Treg markers, which serve as effector lymphoid Treg markers; *Fifth*, int-Treg have seven specific markers and VAT-Treg have 15 specific markers, which serve as tissue effector Treg markers; *Sixth*, seven CD markers shared by int-Treg and VAT-Treg are the non-lymphoid effector Treg markers; *Seventh*, four s-Treg pathways have been identified, which are shared with other three tissue Treg and four CD markers such as TNFRSF18 (CD357), CTLA4 (CD152), TNFRSF1B (CD120b), and TNFRSF4 (CD134) shared by all four tissue Treg, which are the essential core CD markers and functional pathways regardless of tissue environments; and *Eighth*, since these results are transcriptomic data, the future experiments will be needed to verify those novel CD markers with flow cytometry using monoclonal antibodies.

4. Seven, 49, 44, and 79 increased genes out of total 1176 cytokines have been identified for s-Treg, LN-Treg, int-Treg, and VAT-Treg, respectively, suggesting that LN-Treg, int-Treg, and VAT-Treg are more active than s-Treg in generating immunosuppressive and homeostatic cytokines.

**Figure 6:**
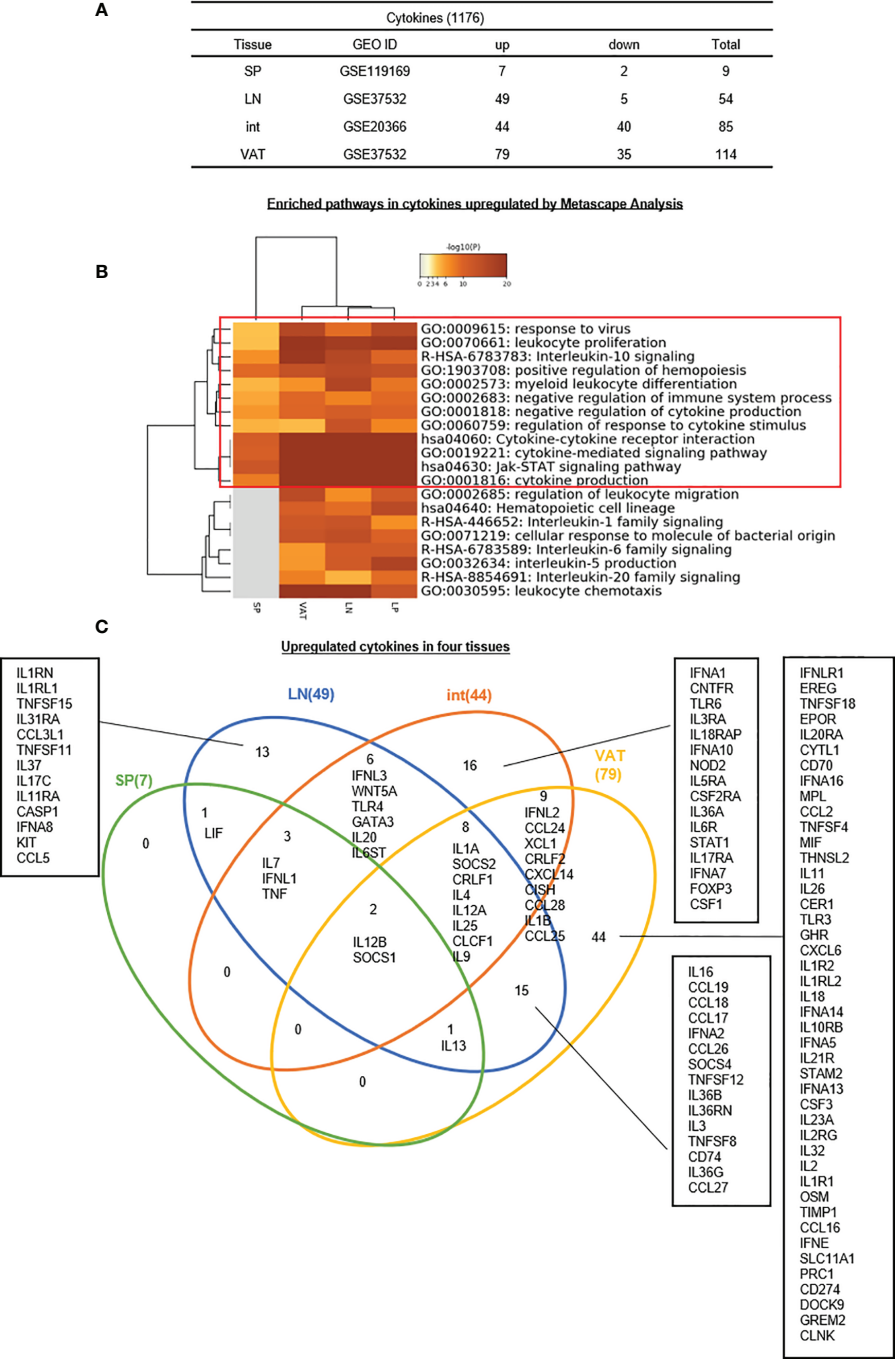
**(A–C)** Thirteen LN Treg specific cytokines, 16 Int Treg-specific cytokines, and 44 VAT Treg-specific cytokines are identified, which mediate 20 immune signaling pathways after screening for total 1,176 cytokines and their interactors (https://www.proteinatlas.org/search/cytokine) (logFC) in four tissues we studied indicated important influence of Treg in cytokine production and modulation of tissue specific microenvironment. Enriched pathway analysis by metascape showed that JAT-STAT signaling pathway and cytokine-cytokine receptor interaction were both modulated by up- and downregulated cytokines in Treg. One interesting finding was that although we have identified that IL2 may be the upstream regulator of Treg modulation; and its receptor IL2RB could induce this modulation, IL2 was downregulated in Treg from Int and VAT, which means a possibility that other interactors of IL2RB participate in this process. As IL2RB could interacts with Jak1 and RACK-1 according to NIH-NCBI Gene database (https://www-ncbi-nlm-nih-gov.libproxy.temple.edu/gene/3560). The metascape analysis also showed the important role of Jak-STAT signaling pathway, which may be a significant pathway in the modulation process of Treg, especially in non-lymphoid tissues such as int and VAT. See the detailed data in **Supplementary Figure 5**.

**Figure 7:**
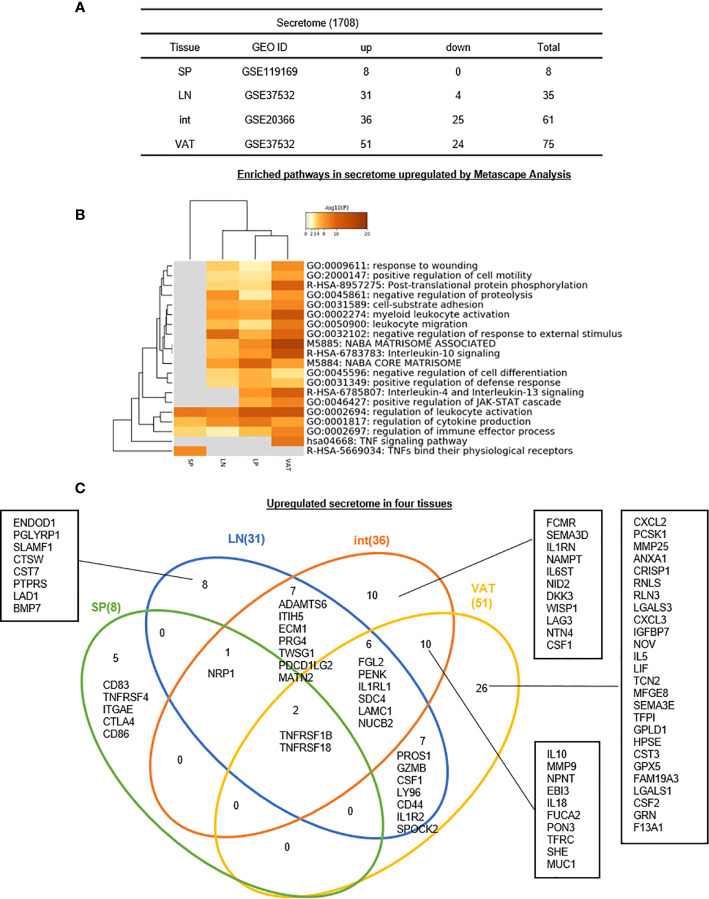
**(A–C)** Five spleen Treg specific secretomic genes, eight lymph nodes Treg specific secretomic genes, 10 intestine Treg-specific secretomic genes, and 26 VAT Treg-specific secretomic genes are identified, which mediate 20 immune signaling pathways after screening for total 1,706 secretome shared (logFC) in four tissues we studied indicated that secretomic changes of Treg in different tissues could mediate the regulation of leukocyte activation, cytokine production and regulation of immune effector process by upregulated secretomic genes in Treg from four tissues we studied. See the detailed data in **Supplementary Figure 6**.

Several functional modes have been identified for Treg such as cytokine secretion such as Amphiregulin secreted from muscle Treg and acted on muscle satellite cells (68), cell surface protein interactions such as co-stimulation receptors and immune checkpoint receptors (105, 106), and granzyme B-mediated killing of target cells, etc (28, 29, 53).. We hypothesized that Treg from different tissues, lymphoid and non-lymphoid, secrete different cytokines from re-shaped Treg transcriptomes. To test this hypothesis, we collected 1,176 cytokines and their interactors (receptors) from a comprehensive protein database (https://www.proteinatlas.org/search/cytokine) as we reported recently (10). As shown in **Figure 6A**, 7, 49, 44, and 79 cytokines were upregulated in s-Treg, LN-Treg, int-Treg and VAT-Treg, respectively. Two, 5, 40, and 35 cytokines were downregulated in s-Treg, LN-Treg, int-Treg and VAT-Treg, respectively.

In addition, the Metascape analysis showed in **Figure 6B** that 12 signal pathways associated with upregulated cytokines were shared among four tissue Treg. These results suggest that these 12 pathways are Treg cytokines-shared pathways and functions. In addition, eight pathways including regulation of leukocyte migration, hematopoietic cell lineage, interleukin-1 family signaling, cell response to molecule of bacterial origin, interleukin-6 family signaling, interleukin-5 signaling, interleukin-20 family signaling, and leukocyte chemotaxis are only shared by LN-Treg and tissue Treg, which are innate immune function pathways.

The Venn Diagram analysis of upregulated cytokines in four tissue Treg showed in **Figure 6C** that seven cytokines in s-Treg all shared with LN-Treg including LIF, IL-7, IFNL1, TNF, IL12B, and SOCS1. LN-Treg had 13 specific cytokines, shared 14 cytokines and 15 cytokines with int-Treg, and VAT-Treg, respectively. Int-Treg had 16 specific cytokines, and shared 19 cytokines with VAT-Treg. VAT-Treg had 44 specific cytokines. These results have demonstrated that *first*, LN-Treg, int-Treg, and VAT-Treg are most secretory tissue Treg in comparison to s-Treg; *second*, LN-Treg, int-Treg, and VAT-Treg have their specific cytokine panels, which have clearly indicated their own immune regulatory functions in addition to the shared Treg functions carried out by only two cytokines such as IL-12B and SOCS1; and *third*, VAT-Treg are highly secretory, which suggests that VAT-Treg play significant homeostatic roles for whole body.

5. Eight, 31, 36, and 51 upregulated genes out of total 1,706 secretomic genes have been identified for s-Treg, LN-Treg, int-Treg, and VAT-Treg, respectively, suggesting that LN-Treg, int-Treg, and VAT-Treg have 13 pathway functions more than s-Treg *via* additional secretomic proteins.

**Figure 8:**
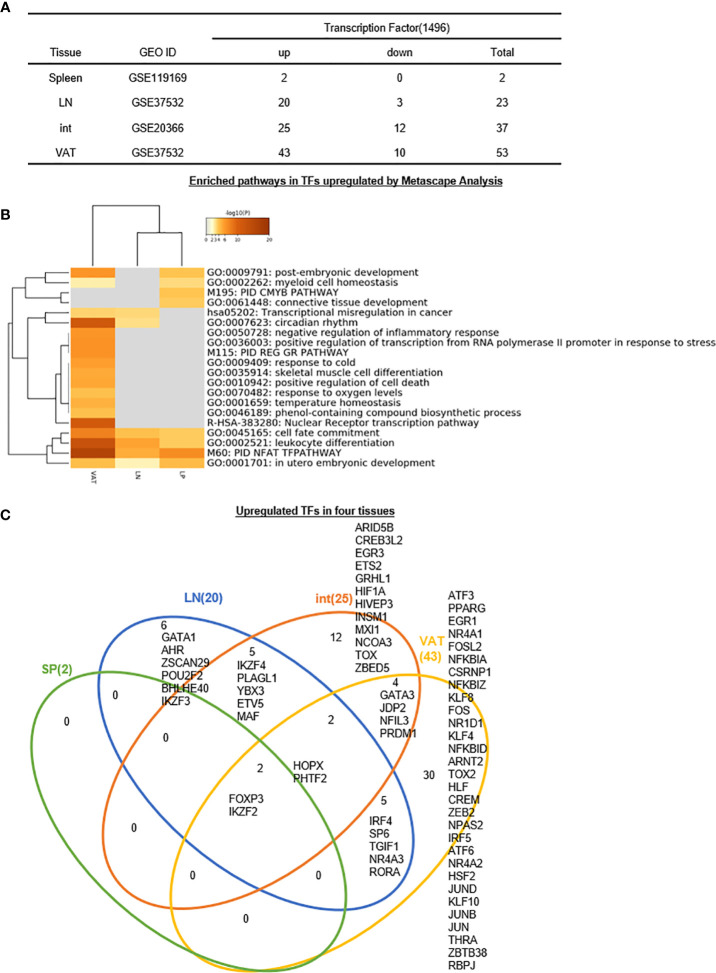
**(A–C)** Six LN Treg specific transcription factors (TFs), 12 intestine Treg-specific TFs, and 30 VAT Treg-specific TFs are identified, which mediate 20 immune signaling pathways after screening for total 1,496 TFs. The original gene lists were all obtained according to the leading program the Human Protein Atlas(HPA, https://www.proteinatlas.org/). See the detailed data in **Supplementary Figure 7**.

As we introduced in the beginning, the secretome, defined as a portion of total proteins secreted by cells to the extracellular space, secures a proper micro-environmental niche, thus maintaining tissue homeostasis (62, 63). Secreted molecules are key mediators in cell-cell interactions and influence the cross-talk with the surrounding tissues in addition to their endocrine functions in long-distance as previously demonstrated by hormones, growth factors, cytokines, adipokines, myokines, cardiokines (64), and chemokines (65). In addition to cytokines discussed in the previous section, we hypothesized that Treg in various tissues have different secretomes to fulfill their immunosuppressive and homeostatic functions. To test this hypothesis, we collected 1,706 secretomic genes from a comprehensive protein database (https://www.proteinatlas.org/search/cytokine) as we reported recently (10). As shown in **Figure 7A**, 8, 31, 36, and 51 secretomic genes were upregulated in s-Treg, LN-Treg, int-Treg, and VAT-Treg, respectively. Zero, 4, 25, and 24 secretomic genes were downregulated in s-Treg, LN-Treg, int-Treg, and VAT-Treg, respectively.

In addition, the Metascape analysis showed in **Figure 7B** that in upregulated secretomes, all four tissue Treg have three shared pathways. In addition, s-Treg have a specific pathway; VAT-Treg also have a specific pathway; int-Treg and VAT-Treg shared two pathways. Moreover, LN-Treg, int-Treg, and VAT-Treg shared 13 pathways.

The Venn Diagram analysis of upregulated secretomes in four tissue Treg showed in **Figure 7C** that s-Treg had five upregulated secretomic genes, s-Treg shared one secretomic gene with LN-Treg, and shared two secretomic genes such as TNFRSF1B and TNFRSF18 with LN-Treg and other two tissue Treg. LN-Treg have eight specific secretomic genes, seven shared with int-Treg, and six shared with int-Treg and VAT-Treg. Int-Treg had 10 specific secretomic genes, and shared six with VAT-Treg. VAT-Treg had 26 specific secretomic genes. Taken together, these results have demonstrated that first, each tissue Treg have their own secretomes in addition to cytokines to fulfill their functions; and second, LN-Treg, int-Treg, and VAT-Treg share 13 pathway functions more than s-Treg; and third, VAT-Treg have more secretomic genes upregulated than other tissue Treg to carry out unique systemic regulatory functions.

6. Two, 20, 25, and 43 increased transcription factors (TFs) out of total 1,496 TFs have been identified for s-Treg, LN-Treg, int-Treg, and VAT-Treg, respectively, suggesting that non-lymphoid tissue (NLT) Treg such as int-Treg and VAT-Treg carry out half to 70% non-immunosuppressive functions, which are not shared with other tissue Treg.

We previously reported that GATA3, HDAC6, and Bcl-6 regulate Foxp3+ Treg plasticity and determine Treg conversion into either novel antigen-presenting cell-like Treg or Th1-Treg (29), suggesting that other T helper cell subsets such as Th2 TF GATA3, Tfh TF Bcl-6 and HDAC6 cooperate with Foxp3 to determine Treg transcriptomes and functions. We hypothesized that tissue Treg have a specific TF sets in addition to Foxp3. To test this hypothesis, we collected 1,496 TFs from a comprehensive protein database (https://www.proteinatlas.org/search/cytokine) as we reported recently (10). As shown in **Figure 8A**, 2, 20, 25, and 43 increased genes out of total 1,496 transcription factors (TFs) have been identified for s-Treg, LN-Treg, int-Treg, and VAT-Treg, respectively. In addition, 0, 3, 12, and 10 downregulated genes out of total 1,496 TFs have been identified for s-Treg, LN-Treg, int-Treg, and VAT-Treg, respectively.

In addition, the Metascape analysis showed in **Figure 8B** that in upregulated TFs, s-Treg had no specific pathways from two upregulated TFs; all three tissue Treg had four shared pathways including cell fate commitment, leukocyte differentiation, PID NFAT TFpathway, *in utero* embryonic development. In addition, VAT-Treg and LN-Treg shared two pathways such as transcriptional misregulation in cancer, and circadian rhythm; and VAT-Treg shared with int-Treg two pathways such as post-embryonic development and myeloid cell homeostasis. Moreover, int-Treg had two specific pathways such as cmyb pathway and connective tissue development.

The Venn Diagram analysis of upregulated TFs in four tissue Treg showed in **Figure 8C** that s-Treg had no specific TFs and had two TFs shared with other three tissue Treg such as Foxp3 and IKZF2. In addition, LN-Treg had six specific TFs; LN-Treg shared five TFs with int-Treg; LN-Treg shared two TFs with int-Treg and VAT-Treg; and LN-Treg shared five TFs with VAT-Treg. Moreover, int-Treg had 12 specific TFs; and int-Treg shared four TFs with VAT-Treg. Finally, VAT-Treg had 30 specific TFs.

Taken together, these results have demonstrated that *first*, s-Treg have no specific TFs out of two upregulated TFs; LN-Treg have six specific TFs (30%) out of 20 upregulated TFs, int-Treg have 12 specific TFs (48%) out of 25 upregulated TFs; and VAT-Treg have 30 specific TFs (69.8%) out of 43 upregulated TFs for their own transcriptomes and functions; *second*, tissue Treg specific TFs allow those Treg to carry out more homeostatic functions than Foxp3-defined immunosuppressive functions; and *third*, int-Treg and VAT-Treg carry out half and 70% non-immunosuppressive functions, which are not shared with other tissue Treg. Our findings are well correlated with previous reports of others (107). For example, *1)* intestine Treg can be classified into three subsets including GATA3^+^Helios^+^ (Nrp1^+^, thymic origin) (107); RORgt^+^Helios^−^ (microbial immunity), and RORgt^−^Helios^−^ (against dietary antigens); *2)* contrary to obese animals, depletion of VAT-Treg in aged animals improves the metabolic parameters and rescues aging-induced insulin resistance (107); and *3)* brown adipose tissue Treg help in thermogenesis (107).

7. Zero, 14, 10, and 16 increased regulators out of total 305 cell death regulatomes in 13 cell death forms have been identified for s-Treg, LN-Treg, int-Treg, and VAT-Treg, respectively; LN-Treg and int-Treg have increased pyroptosis regulators but VAT-Treg have increased apoptosis regulators.

We and others previously reported that Treg cell death pathways related to disease conditions (26–28, 46–52). However, an important question remained unknown that the expression of how many cell death regulators are regulated in tissue Treg in physiological conditions. We hypothesized that the expressions of cell death pathway regulators are differentially regulated in tissue Treg. To test this hypothesis, we collected 13 newly characterized cell death pathway regulators (termed cell death regulatome), total 305 regulatory genes as we reported previously (108). As shown in **Table 7A**, these 13 cell death pathways include apoptosis, autophagy-dependent cell death (ADCD) regulated, anoikis (specific variant of intrinsic apoptosis initiated by the loss of integrin-dependent anchorage) related, entotic cell death [a type of regulated cell death (RCD) that originates from actomyosin-dependent cell-in-cell internalization (entosis) and is executed by lysosomes], ferroptosis [a form of RCD initiated by oxidative perturbations of the intracellular microenvironment that is under constitutive control by glutathione peroxidase 4 (GPX4) and can be inhibited by iron chelators and lipophilic antioxidants], immunogenic cell death (ICD) regulated, lysosome dependent cell death, mitotic catastrophe regulated (oncosuppressive mechanism for the control of mitosis-incompetent cells by RCD or cellular senescence), mitochondrial permeability transition (MPT)-driven necrosis, necroptosis [a modality of RCD triggered by perturbations of extracellular or intracellular homeostasis that critically depends on mixed lineage kinase domain like pseudokinase (MLKL), receptor interacting serine/threonine kinase 3 (RIPK3), and (at least in some settings) on the kinase activity of RIPK1], NETotic [a reactive oxygen species (ROS)-dependent modality of RCD restricted to cells of hematopoietic derivation and associated with neutrophil extracellular trap (NET) extrusion], ParThanatos [A modality of RCD initiated by poly(ADP-ribose) polymerase 1 (PARP1) hyperactivation and precipitated by the consequent bioenergetic catastrophe coupled to Apoptosis-inducing factor (AIF)-dependent and macrophage migration inhibitory factor (MIF)-dependent DNA degradation] (109), and inflammatory cell death [pyroptosis, a type of RCD that critically depends on the formation of plasma membrane pores by members of the gasdermin protein family, often (but not always) as a consequence of inflammatory caspase activation] (110).

**Table 7A:** The expression changes of 305 signature genes of 13 cell death pathways were identified to reveal the main pattern of cell death in Treg from different tissues.

Category		Apoptosis	ADCD regulated	Anoikis related	Entotic cell death	Ferroptosis	ICD regulated	Lysosome dependent cell death	Mitotic cata-strophe regulated	MPT-driven necrosis	Necro-ptosis	NETotic	Par Thanatos	Pyroptosis	Total
Associated gene		102	22	10	23	24	23	7	28	18	10	12	3	23	305
SP	Total	0													
LN	Up	2	1	0	0	0	2	0	3	0	1	0	0	5	14
	Down	0	0	0	1	0	0	0	0	0	0	0	0	0	1
	Total	2	1	0	1	0	2	0	3	0	1	0	0	5	15
Int	Up	1	1	1	1	1	1	0	0	0	1	0	0	3	10
	Down	2	0	1	1	0	0	1	0	0	0	0	0	2	7
	Total	3	1	2	2	1	1	1	0	0	1	0	0	5	17
VAT	Up	5	0	0	2	2	0	0	2	0	2	1	0	2	16
	Down	3	0	0	1	1	1	0	0	0	0	0	0	1	7
	Total	8	0	0	3	3	1	0	2	0	2	1	0	3	23

We found that with the tiers of tissue changes, the number of changed genes were increased, indicating modification of cell death pathways may be one of the mechanisms in Treg development of different tissues.

As shown in **Table 7A**, we identified 305 regulatory genes associated 13 types of cell death pathways. The results showed that s-Treg had no the expression changes of cell death regulators in comparison to that of Tconv. LN-Treg had 14 cell death regulator upregulation including two (out of 102 in total) in apoptosis, one out of 22 regulators in ADCD regulated, two out of 23 regulators in ICD regulated, three out of 28 regulators in mitotic catastrophe regulated, one out of 10 necroptosis regulators, six out of 23 (26.1%) pyroptosis regulators (**Table 7B**) including Naip5, P2Rx7, caspase-1, caspase-4, IFNGR1, and TLR7. In addition, int-Treg had one regulator upregulation in each of seven cell death forms such as apoptosis, ADCD, anoikis, entotic, ferroptosis, ICD, necroptosis and three out of 23 (13%) regulator upregulation in pyroptosis. Moreover, VAT-Treg had five regulator (out of 102, 4.9%) upregulation in apoptosis, two regulator upregulation in each of five cell death forms such as entotic, ferroptosis, mitotic catastrophe, necroptosis, and pyroptosis, and one regulator upregulation in NETotic. Furthermore, LN-Treg had 14 regulator upregulation but only one regulator downregulation (up/down = 14/1); int-Treg had 10 regulator upregulation but seven regulator downregulation (up/down = 10/7); and VAT-Treg had 16 regulator upregulation but seven regulator downregulation (up/down = 16/7) (**Table 7A**). These results have demonstrated that first, s-Treg have no expression changes of cell death regulators in comparison to that of Tconv, suggesting that s-Treg is more protected from cell death stimulations in physiological conditions than other tissue Treg; second, tissue Treg have more upregulation than downregulation of cell death regulators, suggesting that upregulation of cell death regulators is a Treg response to stimuli; third, LN-Treg and int-Treg have more pyroptosis regulator upregulation than VAT-Treg but VAT-Treg have more apoptosis regulator upregulation than other tissue Treg, suggesting that LN-Treg and int-Treg have higher inflammatory cell death and major histocompatibility complex class II (MHC-II)/antigen epitope-T cell antigen receptor (TCR) signaling-independent innate immune potential, as we recently reported (29), in response to stimulations of DAMPs than Tconv in the same tissues and Treg in other tissues, which are well correlated with that reported (111). It has been reported that the activation of the pyroptosis effector NLRP3 inflammasome has a crucial role in the immunoprotection against pulmonary paracoccidioidomycosis by promoting the expansion of Th1/Th17 immunity and reducing the suppressive control mediated by Treg cells (112); loss of Treg cytokine IL10 signaling leads to intestinal inflammation, at least in part, through increased production of IL-1 by innate immune cells, leading to activation of CD4^+^ T cells (113); and particulate matter (PM) exposure leads to an immunosuppressive lung environment with higher recruitment of Treg in the presence of caspase-1 inhibitor (114). These results suggest that immunosuppressive Treg inhibit activation of inflammasomes/caspase-1 activation-pyroptosis. Taken together, our results that increased pyroptosis potential in LN Treg and int-Treg may suggest higher potential of these Treg develop Treg pyroptosis and plasticity than s-Treg and VAT-Treg.

**Table 7B:** Expression modulation of cell death-associated genes showed a tissue-specific pattern.

Category	Gene symbol	logFC			
		SP	LN	Int	VAT
Apoptosis	Tnfrsf10B				2.0850284
	Pmaip1				2.0157141
	Bcl2L1		1.0995571		1.4952833
	Birc3				1.0889109
	Bag3				1.0197759
	Tnfrsf1A				−1.1182975
	Bcl2L11				−1.2775628
	Bcl2			−1.727972	−2.7449579
	Bid			1.54738346	
	Bik			−2.3173606	
	Casp7		1.1105407		
Pyroptosis	Naip5		2.0169637	1.59618642	2.4546001
	Il18			1.83266871	1.3360937
	P2Rx7		1.689888		−1.923491
	Casp1		1.4067099	−2.4469822	
	Casp4		1.5428512	−1.8950963	
	Ifngr1		1.4007254	1.00873405	
	Tlr7		2.1263581		
Ferroptosis	Tfrc			1.17241948	1.4131957
	Got1				1.329216
	Dpp4				−2.1885734
Entotic cell death	Itgav			1.64749073	1.8527778
	Ctnna1				2.0618036
	Itgb1				−2.4862489
	Itgb3		−1.2667506		
	Itga1			−1.8605296	
ICD regulated	Entpd1		3.4476903	1.53291419	
	Nt5E		2.3812727		−1.3166589
Lysosome dependent cell death	Hspa1A			−1.0392272	
Mitotic catastrophe regulated	Wee1		1.4810863		1.5899063
	Chek1		1.1857745		
	Mapkapk2				1.0394488
	Ccnb1		1.0084829		
Necroptosis	Mlkl			1.09476108	1.2619722
	Ripk3				1.188382
NETotic	Map2K3				1.1578226
ADCD regulated	Gabarapl1		1.0626488	1.19783692	
Anoikis related	Ptk2			−1.1185414	
	Src			1.00754235	

8. One, 15, 19, and 31 increased kinases out of total 661 kinome have been identified for s-Treg, LN-Treg, int-Treg, and VAT-Treg, respectively.

**Figure 9:**
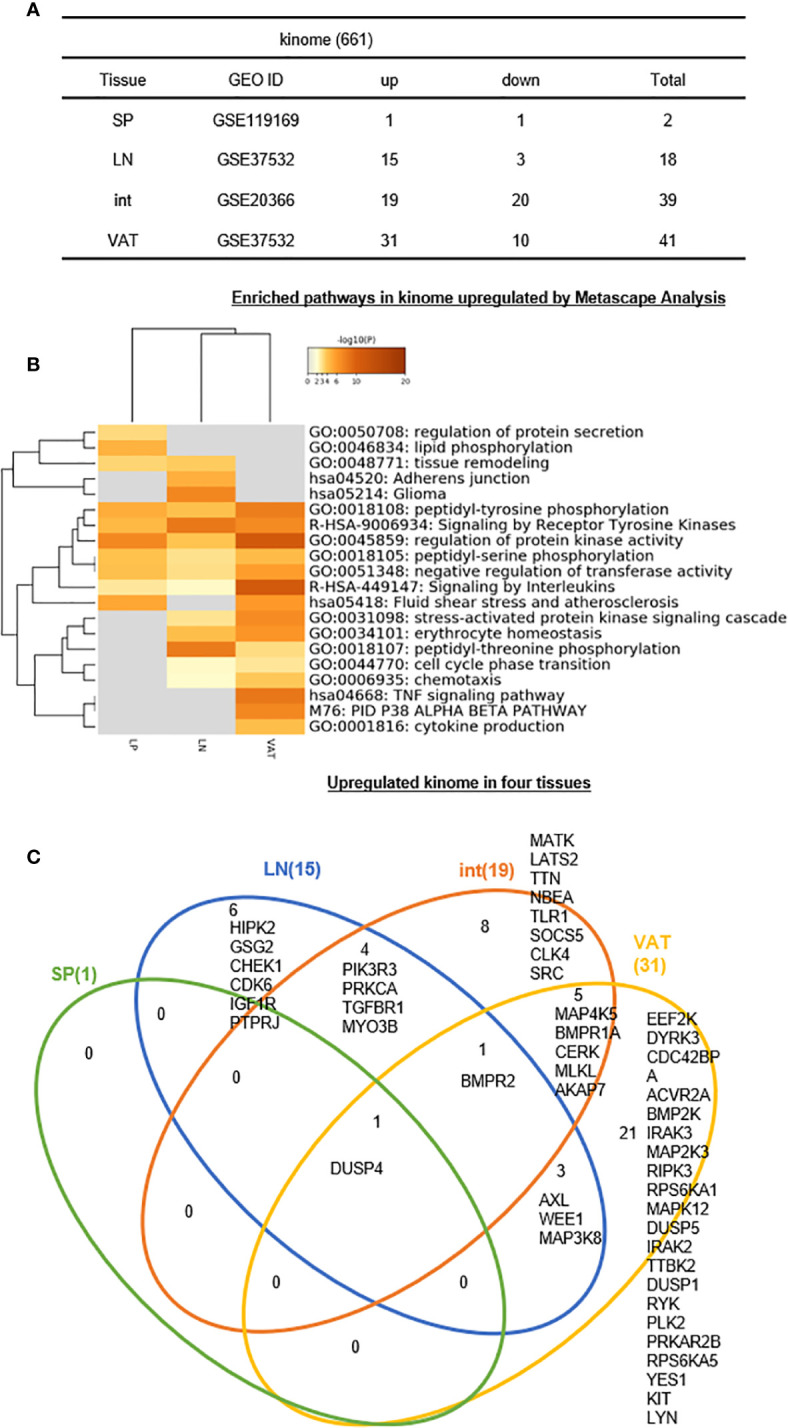
**(A–C)** Six lymph nodes Treg specific kinases, eight intestine Treg-specific kinases, and 21 VAT Treg-specific kinases are identified, which mediate 20 immune signaling pathways after screening for total 661 human kinome expression changes of kinome profile were analyzed in our study. See the detailed data in **Supplementary Figure 8**.

It has been reported that kinases play important roles in regulating Treg functions including serine/threonine mammalian target of rapamycin (mTOR) pathway (115), phosphoinositide 3-kinase δ (PI3K δ) (116), PI3K/Akt/mTOR cascade (117), AMPK (118), non-receptor tyrosine kinase IL-2 inducible T cell kinase (ITK) (119), glycogen synthase kinase 3β (120), STAT5B (121), and NF-kB (101), etc. Also, reduced expression of phosphatase PTPN2 promotes pathogenic conversion of Tregs in autoimmunity (122). However, an important question remained whether tissue Treg have different kinase pathways. We hypothesized that tissue Treg have upregulated kinase pathways that fit their specific functions and Treg shared functions. To test this hypothesis, we examined the expression changes of total kinome (a complete list of 661 kinases encoded in human genome) (123) in four tissue Treg. As shown in **Figure 9A**, 1, 15, 19, and 31 kinases out of 661 total kinome are upregulated in s-Treg, LN-Treg, int-Treg, and VAT-Treg, respectively. In addition, 1, 3, 20, and 10 kinases were downregulated in s-Treg, LN-Treg, int-Treg, and VAT-Treg, respectively. As shown in **Figure 9B** for upregulated kinase pathways, six pathways were shared between int-Treg, LN-Treg, and VAT-Treg. In addition, LN-Treg shared five pathways with VAT-Treg. Moreover, int-Treg shared one pathway with LN-Treg tissue remodeling. Int-Treg shared one pathway with VAT-Treg fluid shear stress and atherosclerosis. Furthermore, int-Treg had two specific pathways such as regulation of protein secretion, and lipid phosphorylation. LN-Treg had two specific pathways. VAT-Treg had three specific pathways.

The Venn Diagram analysis of upregulated kinases in four tissue Treg showed in **Figure 9C** that s-Treg had one kinase DUSP4 shared with other three tissue Treg. In addition, LN-Treg had six specific kinases; LN-Treg shared four kinases with int-Treg; LN-Treg shared one kinase BMPR2 with int-Treg and VAT-Treg; and LN-Treg shared three kinases with VAT-Treg. Moreover, int-Treg had eight specific kinases; and int-Treg shared five kinases with VAT-Treg. Finally, VAT-Treg had 21 specific kinases. These results have demonstrated that *first*, LN-Treg, int-Treg, and VAT-Treg have six, eight, and 21 specific kinase pathways, respectively, for their specific functions; *second*, DUSP4 is the kinase pathway shared between all four tissue Treg including s-Treg; and BMPR2 is the kinase pathway shared between three Treg such as LN-Treg, int-Treg, and VAT-Treg; and *third*, additional three sets of two tissue Treg-shared 12 kinases: *a)* four kinases for LN-Treg and int-Treg, *b)* three kinases LN-Treg and VAT-Treg, and *c)* five kinases for int-Treg and VAT-Treg are all important for tissue Treg functions.

9. IL2Rβ plays essential roles in promoting all tissue Treg shared functions and specific functions.

**Figure 10:**
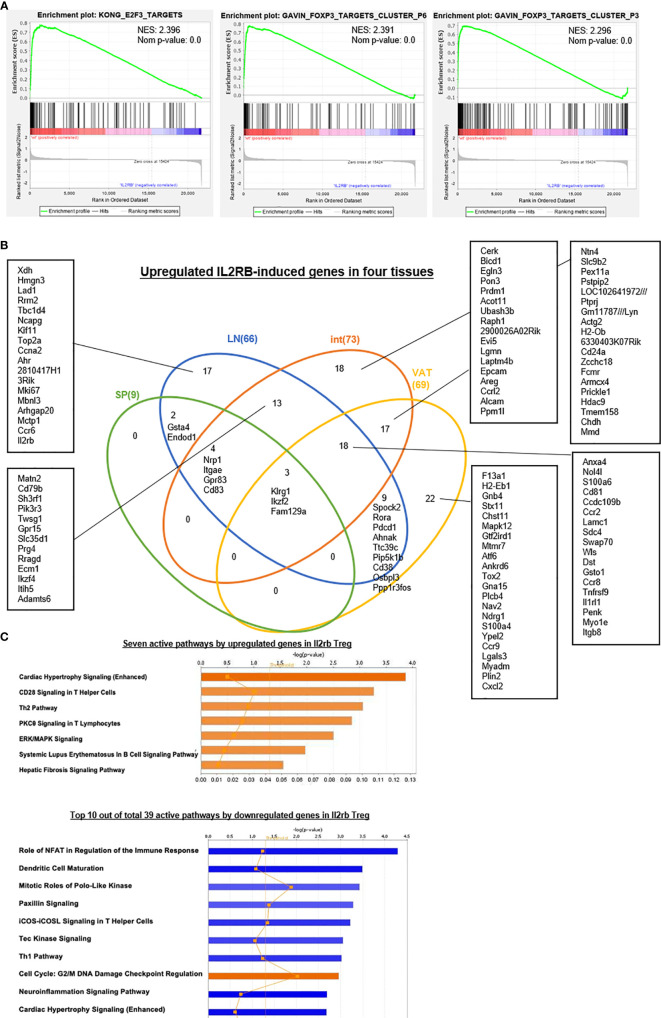
**(A)** The gene set enrichment analysis (GSEA) were applied to investigate the potential mechanisms that how IL2RB modulates Treg signature genes. We found that Il2rb could suppress the function of E2F3 and FOXP3 and thus modulated Treg. Another interesting finding was that Il2rb could suppress some cancer, such as pediatric cancer, liver cancer, and myeloma, indicating its potential role in cancer therapy. **(B)** Venn Diagram showed shared and tissue-specific IL2RB-induced genes both upregulated and downregulated in four tissues we studied. **(C)** Active pathways analyzed by IPA indicated that seven pathways were activated by upregulated genes in Il2rb−/− Treg, including Cardiac Hypertrophy Signaling (Enhanced), CD28 Signaling in T Helper Cells, Th2 Pathway, PKCθ Signaling in T Lymphocytes, ERK/MAPK Signaling, Systemic Lupus Erythematosus In B Cell Signaling Pathway and Hepatic Fibrosis Signaling Pathway. In addition, 39 pathways were activated by downregulated genes in Il2rb−/− Treg, among which three pathways were positively activated including Cell Cycle: G2/M DNA Damage Checkpoint Regulation, RhoGDI Signaling and Endocannabinoid Cancer Inhibition Pathway. All these results indicated Il2rb may mediate the plasticity of Treg in different tissues.

We and others reported that IL-2 signaling pathway is essential for Treg survival (26–28, 46–52); and low dose IL-2 has been developed as a new Treg-based therapy (124). However, a few important questions remained how IL-2 regulate Treg signature genes and cytokines and chemokines. We hypothesized that IL-2 receptor signaling plays a critical role in regulating the expression of Treg signature genes and cytokines and chemokines. To examine this hypothesis, we found IL-2 receptor b (IL2Rβ, CD122) KO Treg microarray datasets (GSE14350) in the NCBI-GeoDatasets database. Of note, IL-2 binds with low affinity to IL-2Rα (CD25) or the common γ -chain (γc, CD132)-IL-2Rβ heterodimers, but receptor affinity increases ~1,000 folds when these three subunits together interact with IL-2. IL-2 shares the common γ -chain (γc)-IL-2Rβ heterodimers with IL-15; and IL2Rβ is indispensable for Treg cell function (125). As shown in **Table 8A**, 87 genes were upregulated and 235 genes were downregulated in IL2Rβ (IL2RB) KO Treg, suggesting that IL2RB induces 235 genes and suppresses 87 genes. We then examined whether IL2RB regulates Treg signature gene expressions by determining the expression of 68 Treg upregulated signature genes and 40 Treg downregulated genes. As shown in **Table 8B**, 12 out of 68 (17.6%) Treg upregulated signature genes were induced by IL-2RB; and four out of 40 (10%) Treg downregulated genes were also inhibited by IL-2RB in Treg. We further examined whether IL2Rb regulates tissue Treg genes differentially. As shown in **Table 8C**, 129 out of 235 IL-2Rb induced genes were upregulated in tissue Treg including 9 genes upregulated in s-Treg, 66 genes upregulated in LN-Treg, 73 genes upregulated in int-Treg, and 69 genes upregulated in VAT-Treg. The Gene Set Enrichment Analysis (GSEA, http://www.gsea-msigdb.org/gsea/index.jsp) showed that IL2RB modulate the functions of E2F3 and Foxp3 (**Figure 10A**). The Venn Diagram analysis of IL2RB upregulated genes in four tissue Treg showed in **Figure 10B** that *first*, s-Treg shared all nine genes with LN-Treg including two genes specifically shared with LN-Treg, four genes specifically shared with int-Treg, and three genes specifically shared with VAT-Treg; *second*, LN-Treg had 17 specific genes, shared 13 genes with int-Treg, shared 18 genes with int-Treg and VAT-Treg, and shared 9 genes with VAT-Treg; and third, int-Treg had 18 specific genes, shared 17 genes with VAT-Treg; finally, VAT-Treg had 22 specific genes.

**Table 8A:** Our data and many other studies identified the function of interleukin-2 (IL2) in facilitating development and maintaining hemostasis of Treg.

IL2RB-/- Treg *vs.* wt Treg (p value < 0.05, |logFC|>1)		
GEO ID	GSE14350	
Up (IL2RB suppressed genes)	Down (IL2RB induced genes)	Total
87	235	322

In order to further discuss its roles of Il2, we applied a microarray dataset comparing IL2-2Rbeta-deficient Treg and wild-type (wt) Treg to identify the roles of Il2rb in Treg (NIH-NCBI Geo Datasets database ID: GSE14350; CD4+CD25hi Treg cells were directly isolated from the spleen of C57BL/6 WT vs. IL2RbWT/Thymus mice by FACS sorting). We identified 235 genes were downregulated while 87 genes were upregulated in Il2rb−/− Treg. According to these changes, we defined IL2RB induced genes and IL2RB suppressed genes (Gene list was shown in **Supplementary Data**).

**Table 8B:** The expression changes of Treg signature genes including 68 upregulated signature genes and 40 downregulated signature genes were identified to determine whether Il2rb could modulate Treg.

Promoted signature (6/108)				Suppressed signature (16/108)				
	Gene symbol	p. value	Log FC			Gene symbol	p. value	Log FC
Upregulation signature (2/68) 2.94%	TGM2	0.00667	2.12768333	Upregulation signature (12/68) 17.65%		IL2RB	0.000102	−4.8216367
						KLRG1	0.000581	−4.7959733
						IL1RL1	0.00262	−3.21797
						ITGAE	0.000159	−3.06763
						IKZF2	0.00236	−3.0146033
						SDC4	0.00128	−2.59206
	GBP2	0.000155	1.30956667			S100A4	0.000745	−2.3235333
						GPR83	0.00061	−2.17642
						NRP1	0.0000256	−1.5508567
						PRDM1	0.00953	−1.54456
						FAM129A	0.000841	−1.1724933
						IKZF4	0.00123	−1.00432
Downregulated signature (4/40) 10%	PDCD1	0.00179	−1.62164	Downregulated signature (4/40) 10%		IGFBP4	0.000037	4.28697667
	CCR8	0.0182	−2.8821033			IL2	0.0000023	3.32267333
	IKZF2	0.00236	−3.0146033			PDE3B	0.0245	1.79125
	LGALS3	0.00000557	−3.6406367			VIPR1	0.00751	1.33250333

Six out of 108 signature genes were upregulated (the expression of upregulated signature genes were increased and expressions of downregulated ones were decreased) while 16 out 108 signature genes were suppressed (the expressions of upregulated signature genes were decreased; and expressions of downregulated ones were increased). This results indicated that Il2rb could promote Treg signatures to maintain the homeostasis of Treg.

**Table 8C:** In order to investigate the modulation of IL2RB in Treg from different tissues, we identified the expression change of IL2RB induced genes in four tissues we studied.

	IL2RB-modulated genes			
	Tissue	Up	Down	Total
IL2RB induced genes	SP	9	0	9
(126)	LN	66	0	66
	int	73	10	83
	VAT	69	6	75
IL2RB suppressed genes	SP	1	2	3
(1)	LN	6	11	17
	int	3	27	30
	VAT	10	21	31

Total 54.8% (129 out of 235) IL2RB induced genes were modulated while 51.72% (45 out of 87) were modulated in these four tissues; indicating the role of IL2RB in Treg.

We further examined the expression of 1,176 cytokines and their receptors and 199 chemokines in IL2Rb KO Treg. As shown in **Table 8D**, 24 out of 1,176 cytokines and cytokine receptors and 8 out of 199 chemokines were induced by IL2Rb. Finally, our IPA analysis showed that, **Figure 10C**, IL2Rb induces 39 pathways. Taken together, these results have demonstrated that IL2Rb plays essential roles in promoting all tissue Treg shared functions and tissue Treg specific functions too.

**Table 8D:** Twenty-four cytokines out of total 1,176 cytokines and cytokine receptors and eight chemokines out of 199 chemokines according to HPA database (https://www.proteinatlas.org/search/cytokine; https://www.proteinatlas.org/search/chemokine) were promoted by IL2RB in Treg; eight cytokines and one chemokine were suppressed by IL2RB in Treg.

	IL2RB promoted cytokine/chemokine			IL2RB suppressed cytokine/chemokine		
	Gene Symbol	p. value	Log FC	Gene Symbol	p. value	Log FC
Cytokine	AREG	0.0341	−1.39817	SOCS3	0.00796	1.2743
	AURKB	0.0485	−1.04236	IL2	0.0000023	3.32267333
	BCL6	0.00517	−1.2783333	TNFSF11	0.0216	1.38351667
	BIRC5	0.0112	−1.85682	PIK3CD	0.0431	1.36664333
	CD38	0.00154	−1.9301033	BMP7	0.00792	1.41885333
	CD83	0.00197	−2.13187	PTPN9	0.000168	1.10329333
	CDC25B	0.000932	−2.40707	CD86	0.0228	1.37924667
	CDCA8	0.0343	−1.27154	STX1A	0.0175	1.04116667
	CEP55	0.0415	−1.26684			
	ECM1	0.000315	−1.6672033			
	F13A1	0.000316	−2.5573367			
	HMOX1	0.0488	−1.0020233			
	IL18R1	0.00442	−1.4419333			
	IL1RL1	0.00262	−3.21797			
	IL2RB	0.000102	−4.8216367			
	ITGA4	0.0324	−1.26069			
	LITAF	0.00721	−1.0614467			
	NRP1	0.0000256	−1.5508567			
	NUSAP1	0.0385	−1.7501567			
	RORA	0.0316	−2.21042			
	STMN1	0.0119	−1.3578133			
	TNFRSF9	0.0256	−1.3178533			
	TPX2	0.00274	−1.0684967			
	TWSG1	0.00186	−1.0704433			
Chemokine	CCL5	0.0334	−3.48414	PADI2	0.00271	1.06578333
	CCR2	0.0334	−1.8350633			
	CCR6	0.000097	−1.9195967			
	CCR8	0.0182	−2.8821033			
	CCR9	0.00192	−1.9802			
	CCRL2	0.0342	−1.12603			
	CXCL2	0.000827	−3.02322			
	GPR15	0.00206	−1.4584967			

10. Antibody bindings to T cell antigen receptor (TCR) CD3, co-stimulation receptors, and immune checkpoint receptors modulate the expression of 108 Treg signature genes.

Our recent report showed that co-signaling receptors including 14 T cell co-stimulation receptors, 10 co-inhibition receptors (immune checkpoint receptors), and 4 dual function receptors regulate T cell plasticity and immune tolerance (105). However, an important question remained whether a panel of antibodies to TCR-CD3, co-stimulation receptors, and immune checkpoint receptors regulate Treg signature gene expressions in four tissue Treg. We hypothesized that ligation with antibodies to TCR-CD3, co-stimulation receptors and immune checkpoint receptors regulate Treg signature gene expressions. As shown in **Table 9** and **Tables S2A** and **S2B**, ligation with antibodies for 1 to 4 h (early time course) and 20 h (late time course) to B- and T-lymphocyte attenuator (BTLA)-early, BTLA-late, CD3-CD28-early, CD3-CD28-late, CD3-early, CD3-late, CD80-early, CD80-late, cytotoxic T-lymphocyte-associated protein 4 (CTLA4, CD152)-early, CTLA4-late, *i*nducible T-cell *cos*timulator (CD278, ICOS)-early, ICOS-late, programmed cell death protein 1 (PD-1, CD279)-early, PD-1-late resulted in increase of five to nine out of 68 Treg upregulated signature genes, respectively. Interestingly, these results have demonstrated that *first*, activation of Treg with anti-CD3 antibody ligation and anti-CD3 and anti-CD28 results in upregulation of Treg signature genes; *second*, antibody ligation of co-stimulation receptors ICOS and CD80 also lead to upregulation of Treg signature genes; and *finally*, blocking immune checkpoint receptors BTLA, CTLA-4, and PD-1 has interesting effects on Treg (61) in upregulating Treg signature genes, which are similar to that seen in antibody ligation of TCR and co-stimulation receptors.

**Table 9:** Treg signature genes from spleen and lymph nodes were stimulated by anti-CD3 and either anti-CD28, -CTLA4, -ICOS, -PD1, -BTLA, or -CD80 antibodies for a time course of 1, 4, 20, and 48 h (hr), respectively (GSE42276).

Category		BTLA early	BTLA late	CD3-28 early	CD3-28 late	CD3 early	CD3 late	CD80 early	CD80 late	CTLA4 early	CTLA4 late	ICOSearly	ICOSlate	PD-1 early	PD-1 late
Upregulated signature	Up	6	5	9	7	7	5	8	6	7	5	6	3	8	8
	Down	2	5	0	9	2	3	1	2	2	3	5	12	2	2
	Total	8	10	9	16	9	8	9	8	9	8	11	15	10	10
Downregulated signature	Up	6	8	9	11	6	7	6	9	5	8	7	11	8	7
	Down	1	1	0	2	1	1	1	1	1	1	2	3	1	1
	Total	7	9	9	13	7	8	7	10	6	9	9	14	9	8
Total		15	19	18	29	16	16	16	18	15	17	20	29	19	18

The 1 and 4 h lysates were defined as the “early” samples, and the 20 h samples were defined as the “late” samples. We found that 27 upregulated signature genes were modulated whereas 27 downregulated signature genes were modulated in stimulated Treg, indicating that co-stimulatory receptors could modify the expressions of Treg signatures.

11. Based on scRNA-Seq-identified six cluster markers in s-Treg, LN, intestine and VAT-Treg increase activated Treg cluster (clusters 1–3) markers; and decrease resting Treg cluster (clusters 4–6) markers.

Single cell RNA sequencing (scRNA-Seq), in an unbiased manner that does not rely on assumptions of cell-type identities, has revolutionized traditional cell type profiling approaches with fluorescence-conjugated antibodies (126, 127). A recent report with scRNA-Seq classified s-Treg into six clusters including S100a4^high^S100a6^high^ cluster 1 (activated), Itgb1^high^ cluster 2 (activated), Dusp2^high^Nr4a1^high^Foxp3^high^IL2ra^high^ cluster 3 (activated), Ikzf2^high^Foxp3^high^ cluster 4 (resting), Bach2^high^ cluster 5 (resting), and Satb1^high^Sell^high^ cluster 6 (resting) (84). This new classification has significantly improved our understanding on heterogeneity of s-Treg, However, heterogeneity of other LN-Treg, int-Treg and VAT-Treg remained poorly characterized. We hypothesized that LN-Treg, int-Treg and VAT-Treg have heterogeneity more than that of s-Treg. As shown in **Table 10**, we found 112 markers for the cluster 1, 112 markers for the cluster 2, 60 markers for the cluster 3, 59 markers for the cluster 4, 34 markers for the cluster 5, and 72 markers for the cluster 6. Since the six clusters were identified in s-Treg, as expected, s-Treg had four markers (3.57%) upregulated for the cluster 1, four markers (3.57%) upregulated for the cluster 2, zero markers changed for the cluster 3, zero markers changed for the cluster 4, zero markers changed for the cluster 5, one marker (1.39%) upregulated for the cluster 6. In addition, LN-Treg had 14 markers (12.5%) upregulated for the cluster 1, 14 markers (12.5%) upregulated for the cluster 2, two markers (3.33%) upregulated for the cluster 3, one marker (1.69%) upregulated for the cluster 4, one marker (2.94%) upregulated for the cluster 5, four markers (5.56%) upregulated for the cluster 6. Moreover, int-Treg had 12 markers (10.71%) upregulated for the cluster 1, 12 markers (10.71%) upregulated for the cluster 2, one marker (1.67%) upregulated for the cluster 3, zero marker upregulated and three markers (5.08%) downregulated for the cluster 4, one marker (2.94%) upregulated and five markers (14.71%) downregulated for the cluster 5, four markers (5.56%) upregulated and three markers (4.17%) downregulated for the cluster 6. Furthermore, VAT-Treg had 21 markers (18.75%) upregulated and six markers (5.36%) downregulated for the cluster 1, 21 markers (18.75%) upregulated and seven markers (6.25%) downregulated for the cluster 2, seven markers (11.67%) upregulated and two markers (3.33%) downregulated for the cluster 3, two markers (3.39%) upregulated and two markers (3.39%) downregulated for the cluster 4, one marker (2.94%) upregulated and four markers (11.76%) downregulated for the cluster 5, six markers (8.33%) upregulated and six markers (8.33%) downregulated for the cluster 6. Taken together, these results have demonstrated that tissue Treg increase more activated Treg cluster (clusters 1–3) markers than s-Treg; and tissue Treg decrease more resting Treg cluster (clusters 4–6) markers than s-Treg.

**Table 10:** Six clusters of Treg from spleen have been identified by single cell RNA sequencing.

Tissue	Cluster 1 (112)			Cluster 2 (112)			Cluster 3 (60)			Cluster 4 (59)			Cluster 5 (34)			Cluster 6 (72)		
	Up	Down	Total	Up	Down	Total	Up	Down	Total	Up	Down	Total	Up	Down	Total	Up	Down	Total
SP	4 (3.57%)	0	4 (3.57%)	4 (3.57%)	0	4 (3.57%)	0	0	0	0	0	0	0	0	0	1 (1.39%)	0	1 (1.39%)
LN	14 (12.5%)	1 (0.89%)	15 (13.39%)	14 (12.5%)	1 (0.89%)	15 (13.39%)	2 (3.33%)	1 (1.67%)	3 (5%)	1 (1.69%)	0	1 (1.69%)	1 (2.94%)	0	1 (2.94%)	4 (5.56%)	0	4 (5.56%)
int	12 (10.71%)	1 (0.89%)	13 (11.6%)	12 (10.71%)	3 (2.68%)	15 (13.39%)	1 (1.67%)	2 (3.33%)	3 (5%)	0	3 (5.08%)	3 (5.08%)	1 (2.94%)	5 (14.71%)	6 (17.65%)	4 (5.56%)	3 (4.17%)	7 (9.72%)
VAT	21 (18.75%)	6 (5.36%)	27 (24.11%)	21 (18.75%)	7 (6.25%)	28 (25%)	7 (11.67%)	2 (3.33%)	9 (15%)	2 (3.39%)	2 (3.39%)	4 (6.78%)	1 (2.94%)	4 (11.76%)	5 (14.70%)	6 (8.33%)	6 (8.33%)	12 (16.67%)

We found that the percentages of six clusters, originally identified in spleen, in four tissue Treg showed that clusters 1 and 2 were the main components of Treg from spleen, while the components of other tissue Treg were more variable and complicated, especially that of VAT. (The details of expression changes of cluster markers were shown in **Supplementary Tables 3A**, **3H**.)

As shown in **Supplementary Table 3A**, s-Treg-specific upregulated cytokines and cytokine receptors discussed previously were expressed differentially in six clusters; and four out of seven cytokines (57.1%) were expressed in more than four clusters of s-Treg such as IL-7, SOCS1, TNF, and LIF. In addition, in **Supplementary Table 3B**, 11 out of 49 (22.4%) LN specific upregulated cytokines were expressed in more than four clusters. Moreover, in **Supplementary Table 3C**, 14 out of 44 (31.8%) intestine specific upregulated cytokines were expressed in more than four clusters. Furthermore, in **Supplementary Table 3D**, 19 out of 79 (24.1%) VAT specific upregulated cytokines were expressed in more than four clusters. Taken together, these results have demonstrated that first, in three tissue Treg, four clusters-shared cytokines and receptors were in 22.4 to 31.8% range; second, there were cytokines and receptors specific for one of two clusters in tissue Treg; and third, many cytokines and receptors in LN-Treg, int-Treg, and VAT-Treg were not expressed in six s-Treg clusters.

As shown in **Supplementary Table 3E**, s-Treg-specific upregulated TF discussed previously were expressed differentially in six clusters; and one out of two TF (50%) were expressed in more than four clusters of s-Treg such as Foxp3. In addition, in **Supplementary Table 3F**, 12 out of 20 (60%) LN specific upregulated TF were expressed in more than four clusters. Moreover, in **Supplementary Table 3G**, 17 out of 25 (68%) intestine specific upregulated TF were expressed in more than four clusters. Furthermore, in **Supplementary Table 3H**, 24 out of 43 (55.8%) VAT specific upregulated TF were expressed in more than four clusters. Taken together, these results have demonstrated that first, in three tissue Treg, four clusters-shared TF were in 55.8 to 68% range; second, there were TF specific for one of two clusters in tissue Treg; and third, a few TF in LN-Treg, int-Treg, and VAT-Treg were not expressed in six s-Treg clusters.

12. Four tissue Treg promote tissue repair by generating secretomes similar to that of stem cells; and sharing transcription factors AHR, ETV5, EGR1, and KLF4 with stem cells.

**Figure 11:**
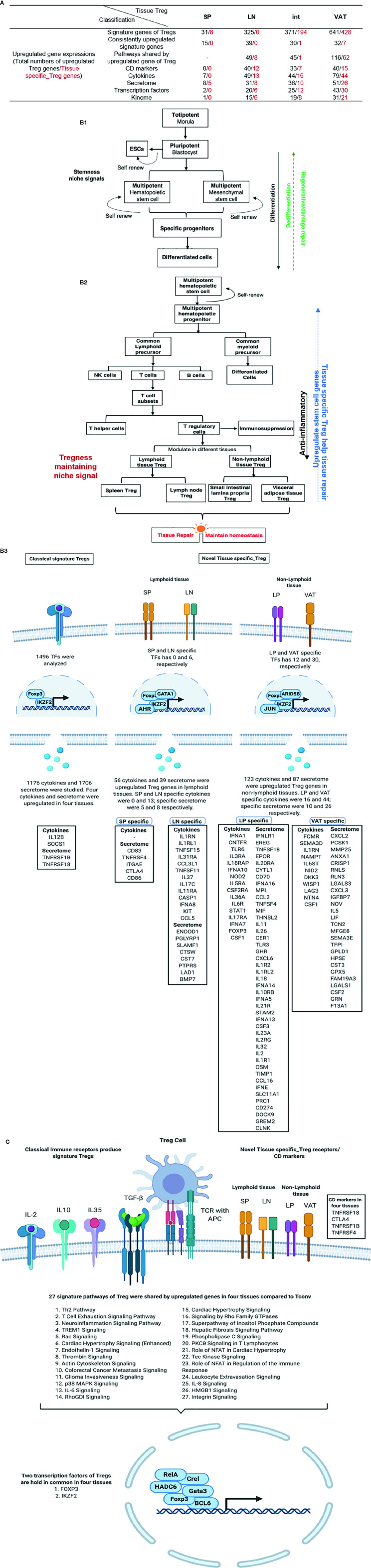
**(A)** A new working model: Stem cell-shared secretomes and transcription factors shared by tissue Treg make tissue Treg as the first T cell type with dual functions of immunosuppression and tissue repair. **(A)** Tissue Treg from spleen, lymph nodes (LN), intestine (int), and visceral adipose tissue (VAT) have been profiled in eight aspects. **(B)** Stem cells in tissues have a special microenvironment or niche in maintaining the stemness with dual functions of self-renewal and differentiate into progenitor cells and differentiated cells. Similar to the stem cells in tissues, tissue Treg also have a special niche in maintaining our newly proposed Tregness with immunosuppression and tissue repair. **(B-1)** Hierarchy of stem cell in homeostasis and regeneration. Stemness niche signal drives stem cell differentiate to specialized lineage populations that maintain tissue homeostasis. During tissue damage, cells can be dedifferentiation in order to increasing proliferation of stem cells. PMID: 25308311. **(B-2)** Hierarchy of Tregs in immunosuppression, tissue repair, and maintaining homeostasis. Tregness maintaining niche signal upregulates tissue specific Treg cytokines and secretome in stem cells cytokines and secretome, which drives Tregs participating in tissue repair and homeostasis. **(C)** Stem cell-shared secretomes and transcription factors make tissue CD4^+^Foxp3^+^ Treg as first T cell type use a Treg niche to maintain their Treg-ness with dual functions of immunosuppression and tissue repair.

Treg have functions in various tissue repair (67) including promoting muscle repair (68), controlling neutrophil recruitment (70) and promoting repair after cardiac injury (69), facilitating skin epithelial stem cell differentiation (71) and wound healing (72), enhancing satellite cell expansion in muscle but blocking satellite cell differentiation (73), facilitating lung resolution (74), promoting lung epithelial cell proliferation (75) and lung injury repair *via* generating the growth factor amphire gulin (68), promoting myelin regeneration in central nerve system (76), and protecting kidney injury (77). However, molecular mechanisms underlying Treg promotion of tissue repair remained poorly defined. We hypothesized that tissue Treg promote tissue repair by generating secretome similar to that of stem cells (128). To examine this hypothesis, we collected four different stem cell (SC) secretomes including human embryonic SC secretome (hESC) with 129 proteins (129), human mesenchymal SC (hMSC) secretome with 51 proteins (130), human visceral adipose SC (hASC) secretome with 182 proteins (131), and human bone marrow SC (hBMSC) secretome with 315 proteins (132) for comparison with our results of tissue Treg secretome and cytokines (**Table 11**). As shown in **Figure 11A**, we made comparison of s-Treg upregulated cytokines and cytokine receptors with seven proteins, s-Treg upregulated secretome with eight proteins, LN-Treg upregulated cytokines and cytokine receptors with 49 proteins, LN-Treg upregulated secretome with 31 proteins, int-Treg upregulated cytokines and cytokine receptors with 44 proteins, int-Treg upregulated secretome with 37 proteins, VAT-Treg upregulated cytokines and cytokine receptors with 79 proteins, and VAT-Treg upregulated secretome with 51 proteins to stem cell secretomes, respectively. The Venn Diagram results were shown in **Table 12**: *1)* s-Treg shared one cytokine out of seven (14.3%), LIF, with hMSC secretome and hASC secretome (**Supplementary Figure 9A**); *2)* LN-Treg secretome shared five out of 31 (16.1%) secretory proteins with SC secretomes including PENK with hASC secretome, sharing LAMC1 with hBMSC secretome, sharing ECM1 and CD44 with secretomes of hBMSC and hASC. In addition, LN-Treg cytokines shared one cytokine, CRLF1, out of 49 upregulated cytokines (2.0%) with hASC secretome (**Supplementary Figure 9B**); *3)* int-Treg shared four out of 37 (11.0%) secretory proteins with SC secretomes including sharing PENK with hASC, sharing ECM1 with hBMSC and hhASC secretomes, and sharing DKK3 and LAMC1 with hBMSC secretome (**Supplementary Figure 9C**); *4)* VAT-Treg shared 12 out of 51 secretomic proteins (23.5%) with SC secretomes including sharing PENK, CXCL2, and TFPI with hASC, sharing LIF with hASC and hMSC secretomes, sharing IGFBP7, CST3, LGALS1, CD44 with hBMSC and hASC secretomes; sharing LAMC1, ANXA1, LGALS3, GRN with hBMSC secretome. In addition, VAT-Treg cytokines shared five cytokines out of 79 upregulated cytokines (6.3%) with SC secretomes including sharing MIF and TIMP1 with hBMSC and hASC secretomes and sharing CRLF1, CCL2, and CXCL6 with hASC secretome (**Supplementary Figure 9D**). Taken together, these results have demonstrated that *first*, tissue Treg share secretomes with stem cell secretomes in the ranges of 11.0 to 23.5%; *second*, LN-Treg and VAT-Treg share cytokines with stem cell secretomes in the ranges of 2.0 to 6.3%, but s-Treg and int-Treg do not share cytokines with stem cell secretomes, suggesting that Treg secretomes are more than Treg cytokines in sharing with stem cell secretomes; and third, for comparison, s-Treg share two out of upregulated eight secretomic genes (25% for s-Treg) with 51 upregulated VAT-Treg secretomic genes (3.9% for VAT-Treg), suggesting that the secretomic gene percentages shared by s-Treg and VAT-Treg are much smaller than that shared by four tissue Treg and stem cells, suggesting that tissue Treg play significant stem cell-like roles for tissue repair and regeneration.

**Table 11:** The cytokine genes and secretomic genes identified in tissue Treg and stem cells were summarized here.

List names	Number of genes
SP_Cytokines	7
SP_Secretome	8
LN_Cytokines	49
LN_Secretome	31
int_Cytokines	44
int_Secretome	37
VAT_Cytokines	79
VAT_Secretome	51
MSC	51
hESCs	129
hASCs	182
hBMSCs	315

**Table 12:** The cytokines and secretomic genes shared between four tissue Treg (SP, LN, int, VAT) and four types of stem cells were summarized (hESCs_PMID: 22984290, hBMSCs_PMID: 22674502, hASCs_PMID: 20184379, and MSCs_PMID: 23685070).

Tissue	Overlapped gene lists	Numbers of overlapped genes	Genes names
SP	[MSC] and [SP_Cytokines] and [hASCs]	1	LIF
LN	[LN_Secretome] and [hASCs]	1	PENK
	[LN_Secretome] and [hBMSCs]	1	LAMC1
	[LN_Secretome] and [hBMSCs] and [hASCs]	2	ECM1
			CD44
	[LN_Cytokines] and [hASCs]	1	CRLF1
int	[int_Secretome] and [hASCs]	1	PENK
	[int_Secretome] and [hBMSCs] and [hASCs]:	1	ECM1
	[int_Secretome] and [hBMSCs]:	2	DKK3
			LAMC1
	[int_Cytokines] and [int_Secretome]:	2	CSF1
			IL6ST
VAT	[VAT_Secretome] and [hASCs]	3	PENK
			CXCL2
			TFPI
	[VAT_Secretome] and [hASCs] and [MSC]	1	LIF
	[VAT_Secretome] and [hBMSCs] and [hASCs]	4	IGFBP7
			CST3
			LGALS1
			CD44
	[VAT_Secretome] and [hBMSCs]:	4	LAMC1
			ANXA1
			LGALS3
			GRN
	[VAT_Cytokines] and [hBMSCs] and [hASCs]:	2	MIF
			TIMP1
	[VAT_Cytokines] and [hASCs]:	3	CRLF1
			CCL2
			CXCL6
	[VAT_Cytokines] and [VAT_Secretome]:	2	IL1R2
			IL18

The Venn diagram figures were shown in **Supplementary Figures 9A**–**9D**.

To further consolidate the transcription regulatory mechanisms for tissue Treg in tissue repair, we then examined a new hypothesis that tissue Treg TFs share stem cell TFs. To test this hypothesis, we performed Venn Diagram analyses with a comparison of upregulated tissue Treg TFs including s-Treg (2 TFs), LN-Treg (20 TFs), int-Treg (25 TFs), and VAT-Treg (43 TFs) to 49 hematopoietic stem cell (HSC) TFs, 41 mesenchymal stem cell (MSC) TFs, and 61 pluripotent stem cell (PSC) TFs (133) (**Table 13**). As shown in **Table 14**, s-Treg did not share any upregulated TFs with stem cell TFs (**Supplementary Figure 10A**); LN TFs shared aryl hydrocarbon receptor (AHR) and E26 transformation-specific (ETS) family TF (ETV5) with MSC TFs (**Supplementary Figure 10B**); int-Treg shared one TF, ETV5, with MSC (**Supplementary Figure 10C**); VAT-Treg shared TF early growth response protein 1 (EGR1, zinc finger protein 268) with HSC, and shared one TF, Krüppel-like factor 4 (KLF4), with PSC (**Supplementary Figure 10D**). AHR plays a significant role in hematopoietic stem cell transcriptome regulation (134). ETV5 plays a critical role in maintaining alveolar type II cells (135), controlling cell type specification in developing mouse brain (136), and having versatile functions in male reproduction (137). EGR1 directs tendon differentiation, promotes tendon repair (138) and blocks energy expenditure *via* direct uncoupling protein 1 (UCP1) transcription repression and counteracts obesity (139). KLF4 is one of 2012 Nobel Laureate Yamanaka’s four key stem cell TFs (140) and a reprogramming factor, and plays an essential role for stem cell maintenance and myeloid and lymphoid cell developments (141). To further determine the causative effects that these four TFs are partially responsible for upregulating Treg genes, as shown in **Table 15**, deficiencies or decreased expressions of the four TFs resulted in partially downregulation of some Treg genes identified in previous eight Results sections. These results suggest that these four Treg-stem cell-shared TFs at least partially promote upregulation of all the eight groups of Treg genes including Treg signature genes, CD markers, cytokines, secretome, TFs, cell death regulators, kinome, and Treg cluster markers. Taken together, our analyses have demonstrated that *first*, tissue Treg both from lymphoid tissue (LN-Treg) and non-lymphoid tissues (int-Treg and VAT-Treg) partially share stem cell TFs; and *second*, tissue Treg upregulated TFs would make Treg play roles in regenerating lung alveolar type II cells, promoting brain cell specification, facilitating male reproduction, promoting tendon differentiation and repair, blocking energy expenditure and inhibiting obesity, and maintaining stem cell and blood cell developments.

**Table 13:** The transcription factor (TF) genes identified in tissue Treg and stem cells were summarized.

List names	Number of TFs
SP_TF	2
LN_TF	20
INT_TF	25
VAT_TF	43
HSC_TF	49
MSC_TF	41
PSC_TF	61

**Table 14:** The transcription factors (TFs) shared between four tissue Treg (SP, LN, int, VAT) and three types of stem cells (HSC, MSC, and PSC) were summarized.

Tissue	Overlapped TF lists	Numbers of overlapped TFs	TF names
SP	–	–	–
LN	[LN_TF] and [HSCs_TF]:	1	AHR
	[LN_TF] and [MSC_TF]:	1	ETV5
INT	[int_TF] and [MSC_TF]:	1	ETV5
VAT	[VAT_TF] and [HSCs_TF]:	1	EGR1
	[VAT_TF] and [PSC_TF]:	1	KLF4

The original stem cell gene lists were obtained from R&D Systems (https://www.rndsystems.com). The Venn diagram figures were shown in **Supplementary Figures S10A** to **S10D**.

**Table 15:** Deficiencies or decreased Treg-stem cell shared transcription factors partially downregulate the upregulated Treg genes identified in 8 *Results* sections.

	(A)	(B)	(C)	(D)	(E)	(F)	(G)	(H)
	**Treg signature genes (42)**	**CD markers (69)**	**Cytokines (118)**	**Secretome (82)**	**TFs (66)**	**Apoptosis and pyroptosis (11)**	**Kinome (49)**	**Cluster markers (269)**
AHR (GSE76276)	1 (2.4%)	1 (1.4%)	1 (0.84%)	–	1 (1.5%)	–	–	2 (0.7%)
ETV5 (GSE30683)	5 (11.9%)	6 (8.7%)	8 (6.8%)	13 (15.9%)	13 (19.7%)	1 (9%)	8 (16.3%)	25 (9%)
EGR1 (GSE60964)	6 (14.3%)	14 (20.3%)	18 (15.3%)	22 (26.8%)	19 (28.8%)	5 (45%)	10 (20.4%)	50 (18.6%)
KLF4 (GSE84742)	1 (2.4%)	4 (5.8%)	2 (1.7%)	2 (2.4%)	2 (3.0%)	–	3 (6.1%)	7 (2.6%)

Deficiency or decreased levels of four transcription factors in **Table 14** (AHR, ETV5, EGR1, AND KLF4) can reverse the expressions of upregulated Treg genes in four tissues (SP, LN, INT, VAT) into downregulation of some Treg genes (the high percentages for each TF were highlighted in red). Deficiency or decreased levels of four transcription factors are from microarray datasets: AHR (GSE76276: AHR KO versus (vs.) AHR floxed), ETV5 (NIH-NCBI Geo datasets database ID GSE30683: ETV5 Knockdown vs. Control), EGR1 (GSE60964: Low level of EGR1 vs. Control), and KLF4 (GSE84742: low levels of KLF4 vs. high levels of KLF4).

Genes in eight columns (A to H) are upregulated genes in four tissues from the data presented in Figures and Tables, which indicate below: Column A: 42 upregulated Treg signature genes came from **Figure 3B**. Column B. 69 upregulated CD markers came from **Figure 5C**. Column C. 118 upregulated cytokines came from **Figure 6C**. Column D. 82 upregulated secretomes came from **Figure 7C**. Column E. 66 upregulated transcription factors came from **Figure 8C**. Column F. 11 were upregulated cell death associated genes (apoptosis and pyroptosis) from **Table 7B**. Column G. 49 were upregulated Kinomes come from **Figure 9C**. Column H. 269 upregulated Cluster markers from **Supplementary Tables 3A** to **3H**.

## Discussion

Treg have been under intensified investigation continuously (51) since being first reported in 1995 (142). Treg are also a new therapeutic target for numerous diseases (143) including cardiovascular disease (144), monogenic disease [immunodysregulation polyendocrinopathy enteropathy X-linked (or IPEX) syndrome], systemic lupus erythematosus, organ-specific autoimmune diseases (type I diabetes, psoriasis, myasthenia, inflammatory bowel disease, and multiple sclerosis), transplantation, and cancers (143, 145). Although many wonderful technological advances including single-cell RNA sequencings (84) have been made to profile lymphoid and non-lymphoid tissue Treg heterogeneity, some important functional issues remained poorly characterized including upregulated signature genes and signaling pathways, CD markers, cytokines and secretomes, TFs, cell death regulatomes, activation and resting status, and Treg similarity of secretomes and TFs to that of stem cells. To address those issues, we performed a comprehensive transcriptomic database mining with the strategies we pioneered (10, 43, 146, 147) to compare four tissue Treg including two lymphoid tissues s-Treg and LN-Treg and two non-lymphoid tissues such as int-Treg and VAT-Treg, and made significant findings.

Our recent paper reported the use of a new function -omics angle to determine the transcriptomic changes of all the human genome-encoded cytokines and secretomes in human peripheral blood mononuclear cells (PBMCs) in patients with chronic kidney disease and end-stage renal disease (10). General transcriptomic analysis of microarrays, RNA-Seq and single-cell RNA-Seq emphasize global transcriptomic profiling regardless the functions of transcripts. Our previous report applied a function angle to examine cytokine changes using a cytokine array in the aorta in atherogenic apolipoprotein E deficient (ApoE−/−) mice in the presence or absence of caspase-1, a key regulator of inflammatory cell death (pyroptosis) (1). In this study, we attempted to multiple function -omics angles plus differentially expressed genes to profile tissue Treg.

Based on our results, we propose a new working model. *First*, as shown in **Figure 11A** including differentially expressed genes, and seven function -omics angles such as: *1)* Treg signature (master regulators and signaling), *2)* pathway analysis (signaling), *3)* human genome-encoded total (HGET) 1,176 cytokines and their receptors (effectors), *4)* 1,706 secretomes (HGET secretory proteins) (effectors), *5)* total 373 CD markers (cell surface receptors for extracellular signals, cell-cell contact effectors, and signaling initiators), *6)* HGET 1,496 transcription factors (nuclear master regulators), *7)* HGET 661 kinases (signaling), and *8)* 305 cell death regulators functional in 13 newly formulated types of cell death to profile both lymphoid tissue Treg (s-Treg and LN-Treg) and non-lymphoid tissue Treg (int-Treg and VAT-Treg). *Second*, these multiple function -omics angles profile from cell surface, intracellular signaling pathways, to nuclear master regulators-transcription factors, and from cell-cell-contact effectors (CD markers) including 28 T cell co-stimulation receptors and immune checkpoint receptors (105) to secretory protein effectors (cytokines and secretomes) to maintain Treg transcriptomic signatures and functional signatures. It has been well documented that stem cell signatures are termed by their stemness with self-renewal capacity and differentiation potential (148), which are maintained by the membranes markers CD73 (5’-nucleotidase to convert AMP to adenosine), CD90 (cell-cell and cell-matrix interactions), and CD105 (endoglin, an accessory receptor for TGF-β), as well as the stemness genes homeobox transcription factor (NANOG), octamer-binding transcription factor 4 (OCT4), sex determining region Y (SRY)-box 2 transcription factor (SOX2), C2H2 zinc-finger transcription factor (REX1, Zfp-42), cell fate controlling membrane receptor NOTCH1 and, type IV intermediate filament protein NESTIN (128, 149). Similarly, we proposed a new concept of Treg-ness for Treg identity maintenance with dual functions of immunosuppression and tissue repair. As demonstrated previously, Treg compartmentalization and trafficking are tissue and organ-specific potentially mediated by distinct chemokine receptors and integrins (150). Similar to stem cells, in order for Treg to maintain their Treg-ness, tissue Treg generate stem cell-shared secretomes and transcription factors to establish a special microenvironment, Treg niche (151, 152), to maintain Treg-ness and suppress Treg plasticity (29, 105), by which tissue Treg can maintain their Treg-ness with dual functions of immunosuppression and tissue repair including promoting stem cell maintenance (153–156) (**Figure 11B**). As shown in **Figure 11C**, our results have demonstrated further that *1)* stem cell-shared secretomes and transcription factors make tissue CD4^+^Foxp3^+^ Treg as first T cell type use a Treg niche to maintain their Treg-ness with dual functions of immunosuppression and tissue repair; *2)* lymphoid tissue Treg share Treg niche and immunosuppressive functions with non-lymphoid tissue Treg; and *3)* non-lymphoid Treg develop more stem cell promoting and tissue repair functions than lymphoid tissue Treg. Our findings have provided novel insights on tissue Treg heterogeneity and new therapeutic targets for immunosuppression, tissue repair, cardiovascular diseases, chronic kidney disease, autoimmune diseases, transplantation, and cancers.

One limitation of the current study is that due to the low throughput nature of verification techniques in the laboratories, we could not verify every result we identified with the analyses of high throughput data [see **Table 1** of Dr. Lai’s paper (106), and **Table 10** of Dr. Zhang’s paper (10) for explanations]. We acknowledge that carefully designed *in-vitro* and *in-vivo* experimental models will be needed to verify all the findings further and underlying mechanisms. Nevertheless, our findings provide novel insights on the roles of tissue Treg in controlling immune responses, and promoting tissue repair and regeneration as well as novel targets for the future therapeutic interventions for immunosuppression, cardiovascular diseases, autoimmune diseases, transplantation, cancers, and tissue repair.

## Data Availability Statement

The original contributions presented in the study are included in the article/**Supplementary Material** further inquiries can be directed to the corresponding author.

## Author Contributions

RZ carried out the data gathering, data analysis and prepared the tables and figures. KX, YSh, YSu, JS, EC, TY, ML, LL, CD, YLu, FS, DN, JW, YLi, RL, XJ, and HW aided the analysis of the data. XY supervised the experimental design, data analysis, and manuscript writing. All authors contributed to the article and approved the submitted version.

## Funding

This work was supported by the hospital fellowship to RZ.

## Conflict of Interest

The authors declare that the research was conducted in the absence of any commercial or financial relationships that could be construed as a potential conflict of interest.

## Supplementary Material

Supplementary Table 1 Supplemental data of housekeeping gene expressions from the datasets of GSE119169, GSE37532, GSE20366, GSE13306, and GS42276.

Supplementary Table 2A Upregulated Treg signature genes were modulated by co-stimulatory molecular antibodies. NR4A1, DUSP4 CD80, P2RY10 were most common upregulated, and CD69, CTLA4 and IL1RL1 were early-phase specific signatures, which could be upregulated by co-stimulatory antibodies; ITGAE, KLRG1, GPR83 and FOS were the most common downregulated Treg signature genes.

Supplementary Table 2B Downregulated Treg signature genes were also modulated by co-stimulatory molecular antibodies. FOSL2, PDE were most common upregulated by co-stimulatory antibody; IGFBP4 were most common downregulated.

Supplementary Table 3A The average expression level in six clusters of both up- and downregulated cytokines in SP we identified were studied.

Supplementary Table 3B The average expression level in six clusters of both up- and downregulated cytokines in LN we identified were studied.

Supplementary Table 3C The average expression level in six clusters of both up- and downregulated cytokines in int we identified were studied.

Supplementary Table 3D The average expression levels of both up- and downregulated cytokines in VAT Treg were analyzed in six Treg clusters.

Supplementary Table 3E The average expression level in six clusters of upregulated TFs in SP we identified were studied.

Supplementary Table 3F The average expression level in six clusters of both up- and downregulated TFs in LN we identified were studied.

Supplementary Table 3G The average expression level in six clusters of both upregulated TFs in int we identified were studied.

Supplementary Table 3H The average expression levels of both upregulated TFs in VAT Treg were analyzed in six Treg clusters.

Supplementary Figure 1 Ingenuity Pathway Analysis also showed expression changes of upregulated and downregulated genes of Treg *vs.* Tconv from different tiers of four tissues we studied were involved in canonical pathways to maintain their roles of homeostasis, the results of active pathways also confirmed the tiers of these four tissues we defined (cutoff: z-score>2).

Supplementary Figure 2 Ingenuity Pathway Analysis of comparison analysis among all the four tissues showed common top 10 pathways by downregulated genes of Treg also indicate with the tier changed, efficiency of the common pathways could be changed responsively.

Supplementary Figure 3 Venn Diagram showed that the Hepatic Fibrosis Signaling Pathyway was shared by downregulated genes of Treg in LN, int, and VAT compared to Tconv, indicating an important function of Treg during hepatic fibrosis which we identified in our latter data. Thirty-six pathways were shared by that of Treg in peripheral tissues int and VAT, indicating their function could be amplified in peripheral non-lymphoid tissues rather that lymphoid tissues.

Supplementary Figure 4 The expression changes of total 373 CD markers (https://www.proteinatlas.org/search/protein_class:CD+markers) shared (logFC) in four tissue Treg indicated the modulation and plasticity of Treg in tissues. The Metascape analysis showed that cytokine-cytokine receptor interactions, TNFs binding their physiological receptors, and cytokine production were all activated by upregulated CD markers, showing important functions of cytokines and TNF receptors of Treg; and Immunoregulatory interactions between a lymphoid and a non-lymphoid cell were downregulated in Treg.

Supplementary Figure 5 Summary of expression changes of total 1176 cytokines and their interactors (https://www.proteinatlas.org/search/cytokine) (logFC) in four tissues we studied indicated important influence of Treg in cytokine production and modulation of tissue specific microenvironment. Enriched pathway analysis by metascape showed that JAT-STAT signaling pathway and cytokine-cytokine receptor interaction were both modulated by up- and downregulated cytokines in Treg. One interesting finding was that although we have indentified IL2 may be the upstream regulator of Treg modulation and its receptor IL2RB could induce this modulation, IL2 was downregulated in Treg from int and VAT, which means a possibility that other interactors of IL2RB participate this process. As IL2RB could interacts with Jak1 and RACK-1 according to PUBMED gene database (https://www-ncbi-nlm-nih-gov.libproxy.temple.edu/gene/3560) and metascape analysis also showed the important role of Jak-STAT signaling pathway, Jak-STAT may be a significant pathway in the modulation process of Treg, especially in non-lymphoid tissues such as int and VAT.

Supplementary Figure 6 The expression changes of total 1,706 secretome shared (logFC) in four tissue Treg indicated that secretomic changes of Treg in different tissue Treg could mediate the regulation of leukocyte activation, cytokine production, and regulation of immune effector process by upregulated secretomic genes in four tissue Treg.

Supplementary Figure 7 Expression changes of total of 1,496 transcription factors (TFs) were analyzed in our studys. The original gene lists were all obtained according to the leading program the Human Protein Atlas(HPA, https://www.proteinatlas.org/).

Supplementary Figure 8 The expression changes of kinome (all the kinases encoded by human genome) were identified in four tissue Treg.

Supplementary Figure 9A The cytokine and secretomic genes shared between splenic Treg and four types of stem cells were identified (hESCs, hBMSCs, hASCs, MSCs), which were illustrated by Venn diagram.

Supplementary Figure 9B The cytokine and secretomic genes shared between lymph node Treg and four types of stem cells (hESCs, hBMSCs, hASCs, MSCs) were identified, which were illustrated by Venn diagram.

Supplementary Figure 9C The cytokine and secretomic genes shared by Treg in small intestinal lamina propria and four types of stem cells (hESCs, hBMSCs, hASCs, MSCs) were identified, which were illustrated by Venn diagram.

Supplementary Figure 9D The cytokine and secretomic genes shared by VAT Treg and four types of stem cells (hESCs, hBMSCs, hASCs, MSCs) were identified, which were illustrated by Venn diagram.

Supplementary Figure 10A Transcription factors (TFs) of Tregs in spleen overlap with three types of stem cells (HSC_TF, MSC_TF, and PSC_TF). There is no overlapped TFs in spleen among other three stem cell groups.

Supplementary Figure 10B The transcription factors (TFs) of Tregs shared by lymph node (LN) Treg and three types of stem cells (HSC_TF, MSC_TF, and PSC_TF) were identified. The overlapped TFs among these Treg and stem cell groups were illustrated by Venn diagram.

Supplementary Figure 10C The transcription factors (TFs) of Tregs in small intestinal lamina propria overlapped with three types of stem cells (HSC_TF, MSC_TF, and PSC_TF) were identified. The overlapped TFs among these four groups were illustrated by Venn diagram.

Supplementary Figure 10D The transcription factors (TFs) of Tregs in visceral adipose tissue overlapped with three types of stem cells (HSC_TF, MSC_TF, and PSC_TF) were identified. The overlapped TFs among these four groups were illustrated by Venn diagram.
