# Supplementary material for: Tissue Treg Secretomes and Transcription Factors Shared With Stem Cells Contribute to a Treg Niche to Maintain Treg-Ness With 80% Innate Immune Pathways, and Functions of Immunosuppression and Tissue Repair
Source: Front Immunol. 2021 Feb 5;11:632239. doi: 10.3389/fimmu.2020.632239 (PMC7892453; doi:10.3389/fimmu.2020.632239)
Supplement: Supplementary Table 1 — Supplemental data of housekeeping gene expressions from the datasets of GSE119169, GSE37532, GSE20366, GSE13306, and GS42276. [file Table_1.docx]

**Table S1. Supplemental data of housekeeping gene expressions from the datasets of GSE119169, GSE37532, GSE20366, GSE13306 and GS42276 .**

|  | **GSE119169 Treg vs Tconv spleen** | | **GSE37532 VAT Treg vs. Tconv** | | **GSE37532 LN Treg vs. Tconv** | | **GSE20366 Treg vs Tconv LP** | |
| --- | --- | --- | --- | --- | --- | --- | --- | --- |
| Gene symbol | p value | logFC | p value | logFC | p value | logFC | P value | logFC |
| ACTB | 0.0459 | 0.094119 | 0.785 | 0.029094 | 0.77 | -0.03563 | 0.559582 | 0.41446 |
| GAPDH | 0.00146 | 0.180642 | 0.188 | -0.14662 | 0.74 | 0.040702 | 0.86141 | -0.16312 |
| PGK1 | 0.413 | -0.03769 | 0.273 | 0.117591 | 0.129 | 0.206691 | 0.595475 | 0.092428 |
| RPLP0 | 0.257 | 0.06301 | 0.00687 | -0.36523 | 0.144 | -0.18742 | 0.699881 | -0.28241 |
| B2M | 0.284 | 0.064523 | 0.17 | 0.196193 | 0.503 | 0.084074 | 0.291313 | 0.191991 |
| YWHAZ | 0.0521 | -0.13095 | 0.00227 | 0.44757 | 0.369 | 0.122164 | 0.093604 | 0.609745 |
| GUSB | 0.108 | -0.09717 | 0.106 | 0.188932 | 0.759 | 0.0423 | 0.514421 | -0.40103 |
| HMBS | 0.382 | -0.03854 | 0.314 | 0.128109 | 0.328 | -0.15825 | 0.200532 | -0.6071 |
| TBP | 0.16 | 0.076802 | 0.489 | -0.0835 | 0.841 | -0.02556 | 0.619519 | 0.090181 |

|  | **GSE13306 Treg vs Tconv Spleen** | | **GSE119169 Treg vs Tconv spleen** | | **GSE37532 VAT Treg vs. Tconv** | | **GSE37532 LN Treg vs. Tconv** | | **GSE20366 Treg vs Tconv LP** | |
| --- | --- | --- | --- | --- | --- | --- | --- | --- | --- | --- |
| Gene symbol | p value | logFC | p value | logFC | p value | logFC | p value | logFC | P value | logFC |
| ACTB | 0.115112 | 0.698 | 0.05 | 0.09 | 0.79 | 0.03 | 0.77 | -0.04 | 0.56 | 0.41 |
| GAPDH | 0.233051 | 0.604 | 0.00 | 0.18 | 0.19 | -0.15 | 0.74 | 0.04 | 0.86 | -0.16 |
| PGK1 | 0.726193 | 0.0485 | 0.41 | -0.04 | 0.27 | 0.12 | 0.13 | 0.21 | 0.60 | 0.09 |
| RPLP0 | 0.940539 | -0.0108 | 0.26 | 0.06 | 0.01 | -0.37 | 0.14 | -0.19 | 0.70 | -0.28 |
| B2M | 0.185436 | 0.205 | 0.28 | 0.06 | 0.17 | 0.20 | 0.50 | 0.08 | 0.29 | 0.19 |
| YWHAZ | 0.043923 | -0.342 | 0.05 | -0.13 | 0.00 | 0.45 | 0.37 | 0.12 | 0.09 | 0.61 |
| GUSB | 0.154641 | 0.249 | 0.11 | -0.10 | 0.11 | 0.19 | 0.76 | 0.04 | 0.51 | -0.40 |
| HMBS | 0.348816 | -0.13 | 0.38 | -0.04 | 0.31 | 0.13 | 0.33 | -0.16 | 0.20 | -0.61 |
| TBP | 0.33482 | -0.155 | 0.16 | 0.08 | 0.49 | -0.08 | 0.84 | -0.03 | 0.62 | 0.09 |

| **Gene symbol** | **p value** | **BTLA early** | **p value** | **BTLA late** | **p value** | **cd3 28 early** | **p value** | **cd3 28 late** | **p value** | **cd3 early** | **p value** | **cd3 late** | **p value** | **cd80 early** | **p value** | **cd80 late** | **p value** | **ctla4 early** | **p value** | **ctla4 late** | **p value** | **icos early** | **p value** | **ctla4 early** | **p value** | **pd1 early** | **p value** | **pd1 late** |
| --- | --- | --- | --- | --- | --- | --- | --- | --- | --- | --- | --- | --- | --- | --- | --- | --- | --- | --- | --- | --- | --- | --- | --- | --- | --- | --- | --- | --- |
| ACTB | 0.402 | -0.13141 | 0.574 | -0.07294 | 0.193 | -0.189 | 0.894 | 0.016417 | 0.148 | -0.19509 | 0.577 | -0.06371 | 0.2 | -0.26102 | 0.863 | -0.02603 | 0.157 | -0.228 | 0.963 | -0.00588 | 0.107 | -0.31669 | 0.994 | -0.00139 | 0.343 | -0.17541 | 0.586 | -0.07415 |
| GAPDH |  |  |  |  |  |  |  |  |  |  |  |  |  |  |  |  |  |  |  |  |  |  |  |  |  |  |  |  |
| PGK1 | 0.0968 | -0.36434 | 0.151 | 0.341449 | 0.215 | -0.259 | 0.000324 | 1.059045 | 0.128 | -0.2726 | 0.461 | -0.1327 | 0.124 | -0.43796 | 0.132 | 0.376801 | 0.139 | -0.325 | 0.509 | 0.151287 | 0.0144 | -0.71818 | 0.00174 | 1.130816 | 0.278 | -0.29594 | 0.724 | -0.0706 |
| RPLP0 | 0.729 | -0.06408 | 0.803 | -0.04067 | 0.463 | -0.139 | 0.323 | 0.168692 | 0.661 | -0.0655 | 0.541 | -0.08131 | 0.346 | -0.19427 | 0.693 | -0.073 | 0.474 | -0.118 | 0.816 | 0.036303 | 0.095 | -0.34566 | 0.646 | 0.096669 | 0.672 | -0.09499 | 0.618 | -0.08469 |
| B2M | 0.794 | -0.03895 | 0.833 | 0.024696 | 0.9 | -0.0158 | 0.763 | -0.0397 | 0.648 | -0.04793 | 0.998 | -0.00035 | 0.722 | -0.06516 | 0.728 | 0.050553 | 0.461 | -0.109 | 0.842 | 0.024728 | 0.939 | -0.01364 | 0.849 | -0.03164 | 0.899 | 0.021083 | 0.869 | 0.021154 |
| YWHAZ | 0.125 | 0.250928 | 0.679 | 0.057223 | 0.151 | 0.21 | 0.416 | 0.103249 | 0.234 | 0.164554 | 0.49 | 0.089493 | 0.5 | 0.134458 | 0.566 | 0.085481 | 0.251 | 0.173 | 0.298 | 0.162046 | 0.39 | 0.161584 | 0.55 | 0.100703 | 0.178 | 0.244266 | 0.516 | 0.104935 |
| GUSB | 0.00226 | -0.63658 | 0.743 | -0.04081 | 0.00132 | -0.609 | 0.0139 | 0.455895 | 0.000368 | -0.6519 | 0.0138 | -0.33115 | 0.0147 | -0.58139 | 0.252 | -0.19305 | 0.00208 | -0.604 | 0.207 | -0.1739 | 0.00165 | -0.76553 | 0.0854 | 0.33399 | 0.0061 | -0.61998 | 0.0434 | -0.34016 |
| HMBS | 0.0318 | -0.47857 | 0.373 | 0.147881 | 0.0413 | -0.369 | 0.00774 | 0.840869 | 0.345 | -0.14302 | 0.371 | -0.15655 | 0.11 | -0.38218 | 0.315 | 0.154607 | 0.0233 | -0.464 | 0.615 | -0.10194 | 0.0333 | -0.50436 | 0.00571 | 0.734148 | 0.219 | -0.26294 | 0.203 | -0.231 |
| TBP | 0.568 | -0.08785 | 0.733 | -0.04282 | 0.632 | -0.0648 | 0.06 | 0.266786 | 0.698 | -0.04301 | 0.397 | 0.113044 | 0.391 | -0.16134 | 0.239 | 0.184516 | 0.386 | -0.126 | 0.381 | 0.123431 | 0.475 | -0.13588 | 0.198 | 0.23145 | 0.5 | -0.11663 | 0.475 | 0.095093 |

**Table S2A. Upregulated Treg signature genes were modulated by co-stimulatory molecular antibodies. NR4A1, DUSP4 CD80, P2RY10 were most common upregulated, and CD69, CTLA4 and IL1RL1 were early-phase specific signatures, which could be upregulated by co-stimulatory antibodies; ITGAE, KLRG1, GPR83 and FOS were the most common downregulated Treg signature genes.**

| Cate-gory | upregulated signature | BTLA early | BTLA  late | CD3-28 early | CD3-28 late | CD3 early | CD3  late | CD80 early | CD80 late | CTLA4 early | CTLA4 late | ICOS  early | ICOS  late | PD-1 early | PD-1  late |
| --- | --- | --- | --- | --- | --- | --- | --- | --- | --- | --- | --- | --- | --- | --- | --- |
| up | NR4A1 | 2.20 | 1.06 | 2.40 |  | 2.18 |  | 2.29 | 1.30 | 2.14 | 1.05 | 2.47 | 1.78 | 2.29 | 2.29 |
| (17) | DUSP4 | 2.14 |  | 2.39 | 1.15 | 2.16 |  | 2.27 | 1.03 | 2.11 |  | 2.15 | 1.24 | 2.37 | 2.37 |
|  | CD80 | 1.11 |  | 1.60 | 1.04 | 1.14 | 1.27 | 1.05 |  | 1.19 |  | 1.76 |  | 1.26 | 1.26 |
|  | P2RY10 |  | 1.03 | 1.24 |  | 1.13 |  | 1.16 | 1.18 | 1.10 | 1.25 |  |  | 1.19 | 1.19 |
|  | CD69 | 1.25 |  | 1.49 |  | 1.32 |  | 1.30 |  | 1.18 |  | 1.49 |  | 1.34 | 1.34 |
|  | CTLA4 | 1.13 |  | 1.17 |  | 1.11 |  | 1.20 |  | 1.14 |  |  |  | 1.21 | 1.21 |
|  | IL1RL1 | 1.39 |  | 1.71 |  | 1.49 |  | 1.64 |  | 1.49 |  |  |  | 1.57 | 1.57 |
|  | SOCS2 |  | 1.39 |  | 1.45 |  | 1.17 |  | 1.28 |  | 1.20 |  |  |  |  |
|  | GBP3 |  | 1.28 |  |  |  | 1.29 |  | 1.40 |  | 1.38 |  |  |  |  |
|  | ZSCAN29 |  | 1.28 |  |  |  | 1.40 |  | 1.59 |  | 1.49 |  |  |  |  |
|  | TNFRSF4 |  |  | 1.11 | 1.11 |  |  |  |  |  |  | 1.04 |  |  |  |
|  | TNFRSF18 |  |  | 1.07 |  |  | 1.03 |  |  |  |  | 1.09 |  |  |  |
|  | LTA |  |  |  | 1.04 |  |  |  |  |  |  |  | 1.09 |  |  |
|  | ARHGAP31 |  |  |  |  |  |  |  |  |  |  |  |  | 1.05 | 1.05 |
|  | PIM2 |  |  |  | 1.48 |  |  |  |  |  |  |  |  |  |  |
|  | BATF |  |  |  | 1.04 |  |  |  |  |  |  |  |  |  |  |
|  | PMAIP1 |  |  |  |  |  |  | 1.03 |  |  |  |  |  |  |  |
| down | ITGAE |  | -1.48 |  | -1.42 |  | -1.41 |  | -1.00 |  | -1.11 | -1.44 | -2.35 |  |  |
| (15) | KLRG1 |  | -1.53 |  | -1.64 |  | -1.16 |  |  |  | -1.13 | -1.92 | -2.06 |  |  |
|  | GPR83 | -1.03 |  |  |  | -1.41 |  | -1.03 |  | -1.47 |  |  |  | -1.46 | -1.46 |
|  | FOS |  | -2.22 |  | -2.39 |  | -2.38 |  | -2.29 |  | -2.46 |  | -1.81 |  |  |
|  | BC005685 | -1.23 |  |  |  | -1.33 |  |  |  | -1.20 |  |  |  | -1.01 | -1.01 |
|  | NRP1 |  | -1.00 |  | -1.39 |  |  |  |  |  |  |  | -1.48 |  |  |
|  | PGLYRP1 |  |  |  | -1.06 |  |  |  |  |  |  | -1.06 | -1.67 |  |  |
|  | S100A4 |  |  |  | -1.19 |  |  |  |  |  |  | -1.21 | -1.87 |  |  |
|  | CD7 |  | -1.05 |  | -1.85 |  |  |  |  |  |  |  | -1.87 |  |  |
|  | IL6RA |  |  |  | -1.38 |  |  |  |  |  |  |  | -1.41 |  |  |
|  | EPSTI1 |  |  |  | -1.37 |  |  |  |  |  |  |  | -1.18 |  |  |
|  | CAPG |  |  |  |  |  |  |  |  |  |  | -1.17 |  |  |  |
|  | IL1RL1 |  |  |  |  |  |  |  |  |  |  |  | -1.08 |  |  |
|  | NT5E |  |  |  |  |  |  |  |  |  |  |  | -1.12 |  |  |
|  | MAF |  |  |  |  |  |  |  |  |  |  |  | -1.64 |  |  |

**Table S2B. Downregulated Treg signature genes were also modulated by co-stimulatory molecular antibodies.** FOSL2, PDE were most common upregulated by co-stimulatory antibody; IGFBP4 were most common downregulated.

| Cate-gory | downregulated signature | BTLA early | BTLA  late | CD3-28 early | CD3-28 late | CD3 early | CD3  late | CD80 early | CD80 late | CTLA4 early | CTLA4 late | ICOS  early | ICOS  late | PD-1 early | PD-1  late |
| --- | --- | --- | --- | --- | --- | --- | --- | --- | --- | --- | --- | --- | --- | --- | --- |
| up | FOSL2 | 2.93 | 1.33 | 3.05 | 1.02 | 3.01 | 1.16 | 2.92 | 1.35 | 2.95 | 1.22 | 3.06 | 1.36 | 3.11 | 1.22 |
| (16) | PDE3B | 1.21 | 1.60 | 1.60 | 1.19 | 1.44 | 1.39 | 1.07 | 2.10 |  | 1.79 | 1.06 | 1.70 | 1.26 | 1.84 |
|  | IL12RB2 | 1.15 | 2.81 | 1.27 | 3.22 |  | 1.87 |  | 2.62 |  | 1.66 | 1.71 | 3.11 | 1.08 | 1.98 |
|  | IL2 |  | 2.68 | 1.82 | 3.53 |  | 1.73 |  | 2.12 |  | 1.47 | 1.60 | 2.16 |  | 1.66 |
|  | CCR8 | 1.93 |  | 2.17 | 1.71 | 1.90 |  | 1.98 |  | 1.84 |  | 2.23 | 2.29 | 1.88 |  |
|  | GZMB |  | 2.20 |  | 2.27 |  | 1.56 |  | 2.37 |  | 1.16 |  | 2.45 |  | 1.61 |
|  | PDCD1 | 1.42 |  | 1.36 |  | 1.60 |  | 1.48 |  | 1.69 | 1.13 |  |  | 1.59 |  |
|  | XCL1 |  | 2.00 |  | 1.99 |  | 1.68 |  | 2.25 |  | 1.91 |  | 3.41 |  | 2.09 |
|  | EZH2 |  | 1.82 |  | 2.06 |  | 1.53 |  | 1.65 |  | 1.25 |  | 1.75 |  | 1.54 |
|  | TBX21 |  | 1.36 | 1.58 | 2.64 |  |  |  | 1.28 |  |  | 1.96 | 2.76 |  |  |
|  | SLC16A3 | 1.40 |  | 1.12 |  | 1.38 |  | 1.37 |  | 1.58 |  |  |  | 1.48 |  |
|  | CXCR4 |  |  | 1.02 |  | 1.14 |  | 1.08 |  | 1.12 |  |  |  | 1.23 |  |
|  | EOMES |  |  |  | 1.37 |  |  |  | 1.15 |  |  |  | 2.75 |  |  |
|  | POLE |  |  |  | 1.04 |  |  |  |  |  |  |  | 1.16 |  |  |
|  | ID2 |  |  |  |  |  |  |  |  |  |  |  |  | 1.06 |  |
|  | EGR1 |  |  |  |  |  |  |  |  |  |  | 1.18 |  |  |  |
| down | IGFBP4 | -1.05 | -2.18 |  | -2.24 | -1.06 | -2.59 | -1.34 | -2.04 | -1.11 | -2.45 |  | -2.12 | -1.08 | -2.36 |
| (4) | FGL2 |  |  |  |  |  |  |  |  |  |  | -1.11 | -1.31 |  |  |
|  | CXCR4 |  |  |  | -1.51 |  |  |  |  |  |  |  | -1.30 |  |  |
|  | LGALS1 |  |  |  |  |  |  |  |  |  |  | -1.12 |  |  |  |

**Table S3A. The average expression level in six clusters of both up- and downregulated cytokines in SP we identified were studied.**

|  | Gene Symbol | spleen_tr_cluster1 | spleen_tr_cluster2 | spleen_tr_cluster3 | spleen_tr_cluster4 | spleen_tr_cluster5 | spleen_tr_cluster6 |
| --- | --- | --- | --- | --- | --- | --- | --- |
| upregulated  cytokine in SP | IL13 | 0 | 0 | 0 | 0 | 0 | 0 |
|  | IL7 | 30.549276 | 5.12820513 | 0 | 10.2309644 | 8.53414208 | 0 |
|  | IFNL1 |  |  |  |  |  |  |
|  | SOCS1 | 32.8518516 | 202.279382 | 155.40693 | 201.486063 | 146.999891 | 139.973836 |
|  | TNF | 0 | 35.2012689 | 164.490803 | 18.0459084 | 13.8733607 | 10.8760672 |
|  | LIF | 0 | 13.9878581 | 12.266176 | 14.563216 | 8.92439046 | 0 |
|  | IL12B | 9.69781605 | 3.95817006 | 0 | 0 | 0 | 0 |
| downregulated cytokine in SP | CCL8 | 0 | 0 | 0 | 3.31193821 | 2.00227458 | 0 |
|  | IL17F |  |  |  |  |  |  |

**Table S3B. The average expression level in six clusters of both up- and downregulated cytokines in LN we identified were studied.**

|  | SYMBOL | spleen_tr_cluster1 | spleen_tr_cluster2 | spleen_tr_cluster3 | spleen_tr_cluster4 | spleen_tr_cluster5 | spleen_tr_cluster6 |
| --- | --- | --- | --- | --- | --- | --- | --- |
| upregulated  cytokine in LN | IL1A | 327.306443 | 272.799666 | 222.367447 | 299.827075 | 285.571084 | 382.322057 |
|  | IL16 | 0 | 0 | 0 | 0 | 0 | 0 |
|  | IFNL3 | 9.69781605 | 3.95817006 | 0 | 0 | 0 | 0 |
|  | IL12B | 0 | 35.2012689 | 164.490803 | 18.0459084 | 13.8733607 | 10.8760672 |
|  | TNF | 0 | 4.78020612 | 0 | 5.91796078 | 0 | 0 |
|  | IL1RN | 0 | 0 | 0 | 0 | 0 | 0 |
|  | IL13 | 58.0394472 | 59.8658235 | 128.489616 | 109.232661 | 81.7309945 | 222.370055 |
|  | SOCS2 | 0 | 0 | 0 | 0 | 0 | 0 |
|  | CRLF1 | 0 | 0 | 0 | 1.36475546 | 0 | 0 |
|  | IL4 | 0 | 7.21155756 | 0 | 13.0946796 | 3.75741181 | 0 |
|  | WNT5A | 11.0458181 | 57.3846953 | 0 | 10.6568911 | 12.209996 | 9.51013305 |
|  | IL1RL1 | 30.549276 | 5.12820513 | 0 | 10.2309644 | 8.53414208 | 0 |
|  | IL7 | 0 | 0 | 0 | 0 | 0 | 0 |
|  | CCL19 |  |  |  |  |  |  |
|  | CCL18 | 0 | 13.9878581 | 12.266176 | 14.563216 | 8.92439046 | 0 |
|  | LIF | 0 | 0 | 0 | 0 | 0 | 0 |
|  | TNFSF15 | 0 | 0 | 0 | 0 | 0 | 0 |
|  | CCL17 | 0 | 0 | 0 | 2.63146815 | 0 | 0 |
|  | TLR4 | 32.8518516 | 202.279382 | 155.40693 | 201.486063 | 146.999891 | 139.973836 |
|  | SOCS1 | 0 | 0 | 0 | 0 | 0 | 0 |
|  | IL31RA | 0 | 0 | 0 | 0 | 0 | 0 |
|  | IFNA2 | 0 | 0 | 0 | 0 | 0 | 0 |
|  | CCL26 | 0 | 3.58916932 | 14.2196943 | 12.4480515 | 0 | 0 |
|  | SOCS4 | 0 | 0 | 0 | 0 | 0 | 0 |
|  | TNFSF12 |  |  |  |  |  |  |
|  | IL36B | 0 | 0 | 0 | 0 | 0 | 0 |
|  | IL12A |  |  |  |  |  |  |
|  | CCL3L1 | 0 | 0 | 0 | 0 | 0 | 0 |
|  | IL25 | 0 | 4.81250481 | 0 | 3.42977871 | 4.00996222 | 6.85294103 |
|  | TNFSF11 |  |  |  |  |  |  |
|  | IL37 | 236.834553 | 160.98833 | 425.540187 | 297.561481 | 205.80146 | 164.829394 |
|  | GATA3 | 0 | 0 | 0 | 0 | 0 | 0 |
|  | IL17C |  |  |  |  |  |  |
|  | IL11RA |  |  |  |  |  |  |
|  | IFNL1 | 0 | 0 | 0 | 0 | 0 | 0 |
|  | CLCF1 |  |  |  |  |  |  |
|  | IL36RN | 0 | 0 | 0 | 0 | 0 | 0 |
|  | IL20 | 0 | 0 | 0 | 0 | 0 | 0 |
|  | IL3 | 67.4196176 | 64.6596167 | 68.942505 | 66.3543627 | 50.8034051 | 88.3728752 |
|  | IL6ST | 0 | 0 | 0 | 8.52934792 | 6.19710365 | 0 |
|  | TNFSF8 | 67.2539109 | 82.7688252 | 14.2196943 | 28.6670175 | 10.0089062 | 0 |
|  | CD74 | 0 | 14.9172513 | 0 | 3.85985634 | 8.21627531 | 0 |
|  | CASP1 |  |  |  |  |  |  |
|  | IFNA8 |  |  |  |  |  |  |
|  | IL36G | 0 | 0 | 0 | 0 | 0 | 0 |
|  | IL9 |  |  |  |  |  |  |
|  | CCL27 | 36.2844702 | 0 | 0 | 8.12195934 | 3.07741832 | 0 |
|  | KIT | 77.1253873 | 110.893781 | 0 | 1.4578152 | 10.0247989 | 0 |
|  | CCL5 |  |  |  |  |  |  |
| downregulated cytokine in LN | SOCS3 | 0 | 0 | 0 | 0 | 0 | 0 |
|  | IL15 | 0 | 0 | 0 | 0 | 0 | 0 |
|  | CCL22 | 0 | 0 | 229.591837 | 20.2766723 | 5.98540176 | 0 |
|  | SOCS6 | 0 | 0 | 0 | 0 | 0 | 0 |
|  | TSLP |  |  |  |  |  |  |

**Table S3C. The average expression level in six clusters of both up- and downregulated cytokines in int we identified were studied.**

|  | SYMBOL | spleen_tr_cluster1 | spleen_tr_cluster2 | spleen_tr_cluster3 | spleen_tr_cluster4 | spleen_tr_cluster5 | spleen_tr_cluster6 |
| --- | --- | --- | --- | --- | --- | --- | --- |
| upregulated  cytokine in int | IL12B | 30.549276 | 5.12820513 | 0 | 10.2309644 | 8.53414208 | 0 |
|  | IL7 | 0 | 0 | 0 | 0 | 0 | 0 |
|  | CRLF1 | 0 | 0 | 0 | 0 | 0 | 0 |
|  | IFNL2 | 0 | 0 | 0 | 0 | 0 | 0 |
|  | CCL24 | 0 | 0 | 0 | 0 | 0 | 0 |
|  | IFNA1 | 0 | 0 | 0 | 0 | 0 | 0 |
|  | IL1A | 0 | 35.2012689 | 164.490803 | 18.0459084 | 13.8733607 | 10.8760672 |
|  | TNF | 0 | 0 | 0 | 0 | 0 | 0 |
|  | IL9 | 32.8518516 | 202.279382 | 155.40693 | 201.486063 | 146.999891 | 139.973836 |
|  | SOCS1 | 0 | 0 | 0 | 0 | 0 | 0 |
|  | CLCF1 | 0 | 0 | 0 | 0 | 2.52260252 | 3.42647051 |
|  | XCL1 | 67.4196176 | 64.6596167 | 68.942505 | 66.3543627 | 50.8034051 | 88.3728752 |
|  | IL6ST | 194.117915 | 94.9889312 | 74.8180334 | 87.7486148 | 41.9712561 | 56.1034758 |
|  | CRLF2 | 58.0394472 | 59.8658235 | 128.489616 | 109.232661 | 81.7309945 | 222.370055 |
|  | SOCS2 | 0 | 0 | 0 | 0 | 0 | 0 |
|  | CNTFR | 0 | 6.80494311 | 40.5510263 | 9.83868156 | 14.5367265 | 11.1870588 |
|  | TLR6 | 0 | 0 | 0 | 0 | 0 | 0 |
|  | IL12A |  |  |  |  |  |  |
|  | IFNL1 | 0 | 0 | 0 | 0 | 2.35417088 | 0 |
|  | CXCL14 | 0 | 0 | 0 | 1.36475546 | 0 | 0 |
|  | IL4 | 0 | 117.887757 | 151.519365 | 146.135357 | 127.491517 | 77.9445585 |
|  | CISH | 30.549276 | 12.9091757 | 0 | 0 | 2.72683219 | 0 |
|  | IL3RA | 0 | 0 | 0 | 4.11881971 | 1.95256821 | 0 |
|  | IL18RAP |  |  |  |  |  |  |
|  | IFNA10 | 0 | 7.21155756 | 0 | 13.0946796 | 3.75741181 | 0 |
|  | WNT5A | 0 | 0 | 0 | 0 | 2.81937929 | 0 |
|  | NOD2 | 0 | 13.4642067 | 61.7283951 | 4.09731953 | 12.4531383 | 6.07614626 |
|  | IL5RA | 0 | 0 | 0 | 3.76593934 | 0 | 0 |
|  | CSF2RA |  |  |  |  |  |  |
|  | IL36A | 0 | 6.87426961 | 0 | 4.22901706 | 4.30165561 | 3.74289318 |
|  | CCL28 | 0 | 0 | 0 | 0 | 0 | 0 |
|  | IL20 |  |  |  |  |  |  |
|  | IL6R | 251.119878 | 280.066074 | 261.217528 | 437.667891 | 364.121748 | 422.481569 |
|  | STAT1 | 104.123768 | 56.4429536 | 62.565668 | 86.1878135 | 116.926813 | 108.256489 |
|  | IL17RA | 0 | 0 | 0 | 0 | 0 | 0 |
|  | IFNA7 | 309.248697 | 351.828231 | 728.194115 | 349.764833 | 433.481706 | 330.117334 |
|  | FOXP3 | 0 | 0 | 0 | 0 | 0 | 0 |
|  | IL25 | 0 | 0 | 0 | 0 | 0 | 0 |
|  | IL1B | 0 | 0 | 0 | 2.63146815 | 0 | 0 |
|  | TLR4 | 0 | 0 | 0 | 2.87369534 | 1.64300196 | 0 |
|  | CCL25 | 0 | 0 | 0 | 0 | 0 | 0 |
|  | IFNL3 | 236.834553 | 160.98833 | 425.540187 | 297.561481 | 205.80146 | 164.829394 |
|  | GATA3 | 89.6344112 | 20.8413107 | 25.5102041 | 4.62890101 | 5.94704277 | 0 |
|  | CSF1 |  |  |  |  |  |  |
| downregulated cyotine int | IL17F | 0 | 0 | 0 | 0 | 0 | 0 |
|  | CSF1R | 0 | 0 | 0 | 0 | 0 | 0 |
|  | IL19 | 14.2661493 | 0 | 0 | 0 | 0 | 0 |
|  | IL9R | 38.5425221 | 176.888592 | 76.5306122 | 217.392478 | 184.517481 | 82.1571235 |
|  | SOCS3 | 0 | 0 | 0 | 0 | 0 | 0 |
|  | CTF1 | 0 | 0 | 0 | 0 | 0 | 0 |
|  | IL17D | 0 | 0 | 0 | 0 | 0 | 0 |
|  | CD70 | 0 | 0 | 0 | 0 | 0 | 0 |
|  | IL15 | 0 | 0 | 0 | 0 | 0 | 0 |
|  | IL5 | 0 | 0 | 0 | 0 | 0 | 8.05749831 |
|  | DOCK3 |  |  |  |  |  |  |
|  | IL37 | 0 | 0 | 0 | 0 | 0 | 0 |
|  | IL1F10 | 0 | 8.49040584 | 0 | 8.9565645 | 4.55402529 | 7.85089579 |
|  | CSF2RB | 0 | 0 | 0 | 17.5980068 | 9.42614021 | 21.1320436 |
|  | SOCS7 |  |  |  |  |  |  |
|  | CCL21 |  |  |  |  |  |  |
|  | CCL14 | 0 | 0 | 0 | 0 | 0 | 0 |
|  | IL17B |  |  |  |  |  |  |
|  | IFNW1 | 0 | 12.847262 | 59.9210485 | 26.2391243 | 20.1902288 | 12.9811125 |
|  | LIFR |  |  |  |  |  |  |
|  | CCL13 | 0 | 0 | 0 | 0 | 0 | 7.9082016 |
|  | TNFSF9 | 0 | 0 | 0 | 0 | 0 | 0 |
|  | IL12RB2 | 0 | 0 | 0 | 0 | 0 | 0 |
|  | IL34 | 0 | 0 | 0 | 0 | 0 | 0 |
|  | IL27 | 0 | 0 | 0 | 0 | 0 | 0 |
|  | FLT3 | 0 | 0 | 0 | 0 | 0 | 0 |
|  | LYN | 0 | 36.7921508 | 40.8535106 | 30.7495738 | 34.0875481 | 54.1462527 |
|  | MAPK14 | 0 | 67.1142348 | 0 | 30.6588189 | 8.97110891 | 9.19354246 |
|  | SOCS5 |  |  |  |  |  |  |
|  | CCL16 | 252.49901 | 253.138808 | 487.031695 | 229.496394 | 216.114147 | 263.229225 |
|  | STAT3 | 0 | 0 | 0 | 0 | 0 | 0 |
|  | IFNB1 | 0 | 0 | 0 | 0 | 0 | 0 |
|  | IL17A | 0 | 0 | 229.591837 | 20.2766723 | 5.98540176 | 0 |
|  | SOCS6 | 0 | 27.2197724 | 0 | 0 | 1.86473232 | 0 |
|  | TNFSF13B | 107.826209 | 71.507057 | 175.238037 | 71.1844902 | 90.1015198 | 53.6525001 |
|  | AIMP1 | 33.7639851 | 26.006061 | 18.1077411 | 20.9208983 | 2.52848466 | 27.687968 |
|  | TGFB1 | 24.8424417 | 74.5864267 | 20.1409869 | 50.6976905 | 10.6182313 | 15.7075446 |
|  | JAK3 | 11.0458181 | 71.6527702 | 98.1294082 | 96.0006342 | 46.3998547 | 23.648607 |
|  | CIAPIN1 |  |  |  |  |  |  |
|  | IL11RA | 208.581065 | 339.302221 | 140.776392 | 160.507953 | 144.125504 | 230.355404 |
|  | CRLF3 |  |  |  |  |  |  |

**Table S3D. The average expression levels of both up- and downregulated cytokines in VAT Treg were analyzed in six Treg clusters.**

|  | SYMBOL | spleen_tr_cluster1 | spleen_tr_cluster2 | spleen_tr_cluster3 | spleen_tr_cluster4 | spleen_tr_cluster5 | spleen_tr_cluster6 |
| --- | --- | --- | --- | --- | --- | --- | --- |
| upregulated  Cytokine in VAT | IFNLR1 | 0 | 0 | 0 | 0 | 2.52260252 | 3.42647051 |
|  | XCL1 | 0 | 0 | 0 | 0 | 0.89745016 | 0 |
|  | EREG | 0 | 0 | 0 | 0 | 0 | 0 |
|  | IL1A | 0 | 0 | 0 | 0 | 0 | 0 |
|  | IFNL2 | 58.0394472 | 59.8658235 | 128.489616 | 109.232661 | 81.7309945 | 222.370055 |
|  | SOCS2 | 0 | 0 | 0 | 0 | 0 | 0 |
|  | TNFSF18 | 9.69781605 | 3.95817006 | 0 | 0 | 0 | 0 |
|  | IL12B | 0 | 0 | 0 | 0 | 0 | 0 |
|  | EPOR | 0 | 3.58916932 | 14.2196943 | 12.4480515 | 0 | 0 |
|  | SOCS4 | 0 | 0 | 0 | 0 | 0 | 0 |
|  | IL9 | 0 | 0 | 0 | 0 | 0 | 0 |
|  | IL12A | 0 | 0 | 0 | 0 | 0 | 0 |
|  | IL20RA | 0 | 0 | 0 | 0 | 0 | 0 |
|  | CYTL1 | 0 | 0 | 0 | 1.36475546 | 0 | 0 |
|  | IL4 |  |  |  |  |  |  |
|  | CCL18 | 0 | 0 | 0 | 0 | 0 | 0 |
|  | CD70 | 0 | 0 | 0 | 0 | 0 | 0 |
|  | IFNA16 | 0 | 0 | 0 | 0 | 0 | 0 |
|  | MPL | 0 | 0 | 0 | 0 | 0 | 0 |
|  | CCL2 | 0 | 0 | 0 | 0 | 0 | 0 |
|  | CRLF1 | 0 | 0 | 0 | 0 | 0 | 0 |
|  | CCL24 | 0 | 0 | 0 | 0 | 0 | 0 |
|  | TNFSF4 |  |  |  |  |  |  |
|  | IL36RN | 0 | 0 | 0 | 0 | 0 | 0 |
|  | TNFSF12 | 235.885882 | 135.784282 | 245.14983 | 242.807242 | 200.872956 | 178.103877 |
|  | MIF | 0 | 0 | 0 | 0 | 0 | 0 |
|  | IL1B | 0 | 0 | 0 | 0 | 0 | 0 |
|  | IFNA2 | 0 | 0 | 0 | 0 | 0 | 0 |
|  | CCL17 |  |  |  |  |  |  |
|  | CCL27 | 0 | 0 | 0 | 0 | 1.60098364 | 0 |
|  | THNSL2 | 0 | 0 | 0 | 0 | 0 | 0 |
|  | IL3 | 0 | 0 | 0 | 0 | 0 | 0 |
|  | IL13 | 86.068954 | 48.96576 | 58.5283134 | 25.7720181 | 32.3329139 | 26.6500739 |
|  | IL11 |  |  |  |  |  |  |
|  | IL26 | 35.5280883 | 13.1390975 | 0 | 7.84748326 | 6.11798956 | 0 |
|  | CER1 |  |  |  |  |  |  |
|  | IL36B | 0 | 0 | 0 | 0 | 0 | 0 |
|  | TLR3 | 0 | 0 | 0 | 0 | 0 | 0 |
|  | GHR | 0 | 0 | 0 | 2.87369534 | 1.64300196 | 0 |
|  | CCL25 | 0 | 0 | 0 | 0 | 0 | 0 |
|  | IL25 |  |  |  |  |  |  |
|  | CXCL6 | 0 | 9.73463388 | 0 | 6.24375624 | 0 | 0 |
|  | IL1R2 | 0 | 0 | 0 | 8.52934792 | 6.19710365 | 0 |
|  | TNFSF8 | 0 | 0 | 0 | 0 | 0 | 0 |
|  | IL1RL2 | 0 | 0 | 0 | 0 | 0 | 0 |
|  | IL18 | 0 | 0 | 0 | 0 | 0 | 0 |
|  | IFNA14 | 36.2844702 | 144.986815 | 30.9984919 | 137.779527 | 45.3723382 | 76.1676693 |
|  | IL10RB | 67.2539109 | 82.7688252 | 14.2196943 | 28.6670175 | 10.0089062 | 0 |
|  | CD74 | 0 | 0 | 0 | 0 | 0 | 0 |
|  | IFNA5 | 0 | 117.887757 | 151.519365 | 146.135357 | 127.491517 | 77.9445585 |
|  | CISH | 84.1188814 | 151.204206 | 75.8685726 | 113.200456 | 138.32781 | 107.230718 |
|  | IL21R | 0 | 8.60437102 | 79.3977274 | 59.999491 | 16.4876894 | 63.2785023 |
|  | STAM2 | 0 | 0 | 0 | 0 | 0 | 0 |
|  | IFNA13 | 0 | 0 | 0 | 0 | 0 | 0 |
|  | CSF3 |  |  |  |  |  |  |
|  | IL36G | 0 | 0 | 0 | 0 | 0 | 0 |
|  | IL23A | 0 | 0 | 0 | 0 | 0 | 0 |
|  | CCL26 | 194.117915 | 94.9889312 | 74.8180334 | 87.7486148 | 41.9712561 | 56.1034758 |
|  | CRLF2 | 1303.70027 | 838.242166 | 863.375151 | 792.704049 | 822.602605 | 1094.43219 |
|  | IL2RG | 32.8518516 | 202.279382 | 155.40693 | 201.486063 | 146.999891 | 139.973836 |
|  | SOCS1 | 0 | 0 | 0 | 0 | 0 | 0 |
|  | CLCF1 |  |  |  |  |  |  |
|  | IL32 | 0 | 0 | 0 | 0 | 0 | 0 |
|  | IL2 | 18.7984059 | 18.6983758 | 49.3241964 | 21.9515028 | 14.1436124 | 44.8708428 |
|  | IL1R1 | 109.494231 | 0 | 0 | 6.68280048 | 16.0548265 | 1.71323526 |
|  | OSM | 0 | 6.87426961 | 0 | 4.22901706 | 4.30165561 | 3.74289318 |
|  | CCL28 | 0 | 0 | 0 | 3.72761567 | 0 | 0 |
|  | TIMP1 |  |  |  |  |  |  |
|  | CCL16 | 0 | 0 | 0 | 0 | 0 | 0 |
|  | IFNE | 0 | 0 | 0 | 0 | 2.35417088 | 0 |
|  | CXCL14 | 9.04339019 | 32.6307038 | 27.7970813 | 29.6363915 | 33.6170994 | 25.8633863 |
|  | SLC11A1 | 9.69781605 | 54.0640322 | 64.4622426 | 43.5396602 | 81.3384427 | 94.0386743 |
|  | PRC1 | 327.306443 | 272.799666 | 222.367447 | 299.827075 | 285.571084 | 382.322057 |
|  | IL16 | 162.299437 | 51.4070343 | 64.4537544 | 52.0234716 | 42.7776743 | 65.4333478 |
|  | CD274 | 0 | 0 | 0 | 0 | 0 | 0 |
|  | CCL19 | 0 | 29.303873 | 0 | 2.62267565 | 5.51648383 | 0 |
|  | DOCK9 | 0 | 0 | 0 | 0 | 0 | 0 |
|  | GREM2 | 0 | 0 | 0 | 0 | 0 | 0 |
|  | CLNK |  |  |  |  |  |  |

|  | SYMBOL | spleen_tr_cluster1 | spleen_tr_cluster2 | spleen_tr_cluster3 | spleen_tr_cluster4 | spleen_tr_cluster5 | spleen_tr_cluster6 |
| --- | --- | --- | --- | --- | --- | --- | --- |
| downregulated cytokine in VAT | IL5 | 0 | 0 | 0 | 0 | 0 | 0 |
|  | IL17D | 14.2661493 | 0 | 0 | 0 | 0 | 0 |
|  | DOCK1 | 0 | 0 | 0 | 0 | 0 | 0 |
|  | IL17B | 0 | 0 | 0 | 0 | 0 | 0 |
|  | IL17A |  |  |  |  |  |  |
|  | CCL14 | 0 | 12.847262 | 59.9210485 | 26.2391243 | 20.1902288 | 12.9811125 |
|  | LIFR | 38.5425221 | 176.888592 | 76.5306122 | 217.392478 | 184.517481 | 82.1571235 |
|  | SOCS3 | 0 | 0 | 0 | 0 | 0 | 0 |
|  | CCL20 |  |  |  |  |  |  |
|  | IFNA17 | 0 | 0 | 0 | 3.31193821 | 2.00227458 | 0 |
|  | IL17F | 0 | 0 | 0 | 0 | 0 | 7.9082016 |
|  | TNFSF9 | 0 | 0 | 0 | 0 | 0 | 0 |
|  | TNFSF15 | 0 | 0 | 0 | 0 | 0 | 0 |
|  | IFNB1 | 0 | 0 | 0 | 0 | 0 | 0 |
|  | IFNK | 0 | 0 | 0 | 0 | 0 | 0 |
|  | IL31RA | 0 | 0 | 0 | 0 | 0 | 0 |
|  | IL1F10 | 0 | 0 | 0 | 0 | 0 | 0 |
|  | IL19 | 0 | 0 | 0 | 0 | 0 | 0 |
|  | CSF2 | 0 | 0 | 0 | 0 | 0 | 0 |
|  | IL15 | 29.6570991 | 3.58916932 | 33.4351782 | 29.6621598 | 29.4898181 | 16.8621158 |
|  | CSF3R | 0 | 67.1142348 | 0 | 30.6588189 | 8.97110891 | 9.19354246 |
|  | SOCS5 | 0 | 0 | 229.591837 | 20.2766723 | 5.98540176 | 0 |
|  | SOCS6 | 0 | 0 | 0 | 0 | 0 | 0 |
|  | OSMR |  |  |  |  |  |  |
|  | CCL4L2 | 0 | 0 | 0 | 0 | 0 | 0 |
|  | IL21 |  |  |  |  |  |  |
|  | CCL13 |  |  |  |  |  |  |
|  | CCL21 |  |  |  |  |  |  |
|  | IFNA21 | 22.413484 | 7.24732211 | 0 | 0 | 16.9875931 | 6.32159203 |
|  | IL22RA1 | 0 | 0 | 0 | 0 | 0 | 0 |
|  | CCL22 | 0 | 0 | 0 | 0 | 0 | 0 |
|  | IL6 | 0 | 0 | 0 | 0 | 0 | 0 |
|  | IFNA4 | 0 | 0 | 0 | 0 | 0 | 0 |
|  | IL17RC | 0 | 0 | 0 | 0 | 0 | 0 |
|  | IFNA6 |  |  |  |  |  |  |

**Table S3E. The average expression level in six clusters of upregulated TFs in SP we identified were studied.**

|  | SYMBOL | spleen_tr_cluster1 | spleen_tr_cluster2 | spleen_tr_cluster3 | spleen_tr_cluster4 | spleen_tr_cluster5 | spleen_tr_cluster6 |
| --- | --- | --- | --- | --- | --- | --- | --- |
| upregulated TF in SP | FOXP3 | 816.809438 | 525.60076 | 475.207069 | 408.896001 | 285.237522 | 261.408924 |
|  | IKZF2 |  |  |  |  |  |  |

**Table S3F. The average expression level in six clusters of both up- and downregulated TFs in LN we identified were studied.**

|  | SYMBOL | spleen_tr_cluster1 | spleen_tr_cluster2 | spleen_tr_cluster3 | spleen_tr_cluster4 | spleen_tr_cluster5 | spleen_tr_cluster6 |
| --- | --- | --- | --- | --- | --- | --- | --- |
| upregulated  TFs in LN | FOXP3 | 816.809438 | 525.60076 | 475.207069 | 408.896001 | 285.237522 | 261.408924 |
|  | IKZF2 | 102.639921 | 60.3365575 | 138.409221 | 97.1652458 | 71.4244416 | 23.0439891 |
|  | HOPX | 119.706392 | 52.0069557 | 50.3069572 | 21.555766 | 31.1861293 | 23.7072468 |
|  | IKZF4 | 0 | 13.7392552 | 230.77757 | 25.028742 | 3.96404047 | 0 |
|  | IRF4 | 0 | 0 | 0 | 44.2205641 | 30.6842739 | 43.6371586 |
|  | GATA1 | 0 | 9.06451465 | 0 | 37.0030716 | 41.0876967 | 80.1347806 |
|  | SP6 | 0 | 0 | 0 | 0 | 0 | 9.11028916 |
|  | PLAGL1 | 0 | 75.1714666 | 0 | 34.7508496 | 40.0799725 | 57.9793682 |
|  | PHTF2 | 161.594493 | 153.060086 | 89.90177 | 63.321099 | 51.7388551 | 49.9434509 |
|  | YBX3 | 0 | 24.6643186 | 0 | 9.68727007 | 7.97290681 | 0 |
|  | AHR | 0 | 0 | 0 | 15.1156467 | 17.6549606 | 0 |
|  | ETV5 | 69.1961711 | 34.0319044 | 20.1409869 | 48.3764394 | 42.7056947 | 41.6851127 |
|  | ZSCAN29 | 27.1301706 | 0 | 85.3181657 | 8.53515417 | 29.8305114 | 0 |
|  | TGIF1 | 422.990278 | 204.992506 | 91.0125142 | 23.3539514 | 45.0813532 | 23.7133226 |
|  | MAF | 0 | 0 | 0 | 0 | 0 | 0 |
|  | NR4A3 | 87.7974795 | 99.2519334 | 146.036657 | 186.086834 | 85.3208654 | 149.518206 |
|  | POU2F2 | 0 | 8.43454791 | 0 | 0 | 21.0186699 | 4.4769571 |
|  | BHLHE40 | 24.3098894 | 81.5080973 | 20.5444273 | 58.7261793 | 15.8705925 | 35.9517242 |
|  | IKZF3 | 0 | 0 | 0 | 0 | 2.89414763 | 0 |
|  | RORA |  |  |  |  |  |  |
| downregulated TFs in LN | EPAS1 | 19.7441163 | 142.842389 | 125.784282 | 129.645428 | 167.796185 | 246.487583 |
|  | TCF7 | 345.143681 | 49.9510326 | 79.6575748 | 34.1844935 | 33.179563 | 18.1842721 |
|  | ID2 |  |  |  |  |  |  |

**Table S3G. The average expression level in six clusters of both upregulated TFs in int we identified were studied.**

|  | SYMBOL | spleen_tr_cluster1 | spleen_tr_cluster2 | spleen_tr_cluster3 | spleen_tr_cluster4 | spleen_tr_cluster5 | spleen_tr_cluster6 |
| --- | --- | --- | --- | --- | --- | --- | --- |
| upregulated TFs in int | ARID5B | 24.7182124 | 0 | 0 | 0 | 7.74647505 | 6.9690782 |
|  | CREB3L2 | 0 | 0 | 0 | 0 | 0 | 0 |
|  | EGR3 | 15.0829563 | 7.3176443 | 119.777379 | 42.006298 | 23.3685055 | 9.29670432 |
|  | ETS2 | 0 | 0 | 0 | 15.1156467 | 17.6549606 | 0 |
|  | ETV5 | 309.248697 | 351.828231 | 728.194115 | 349.764833 | 433.481706 | 330.117334 |
|  | FOXP3 | 236.834553 | 160.98833 | 425.540187 | 297.561481 | 205.80146 | 164.829394 |
|  | GATA3 | 0 | 11.9761913 | 0 | 3.32798637 | 0 | 0 |
|  | GRHL1 | 375.215182 | 140.690907 | 195.136785 | 104.621601 | 54.7101326 | 63.4727893 |
|  | HIF1A | 61.14712 | 3.8681398 | 19.4080543 | 0 | 7.90735705 | 0 |
|  | HIVEP3 | 102.639921 | 60.3365575 | 138.409221 | 97.1652458 | 71.4244416 | 23.0439891 |
|  | HOPX | 816.809438 | 525.60076 | 475.207069 | 408.896001 | 285.237522 | 261.408924 |
|  | IKZF2 | 119.706392 | 52.0069557 | 50.3069572 | 21.555766 | 31.1861293 | 23.7072468 |
|  | IKZF4 | 0 | 5.5356273 | 0 | 23.2351063 | 14.0403484 | 30.3669354 |
|  | INSM1 | 0 | 11.4982178 | 0 | 0 | 1.33543085 | 0 |
|  | JDP2 | 422.990278 | 204.992506 | 91.0125142 | 23.3539514 | 45.0813532 | 23.7133226 |
|  | MAF | 49.0916631 | 29.5259536 | 22.9357798 | 33.8591941 | 19.5068656 | 0 |
|  | MXI1 | 38.6628657 | 167.315308 | 133.304254 | 173.299101 | 155.386362 | 185.492969 |
|  | NCOA3 | 0 | 34.7266527 | 0 | 0 | 0 | 0 |
|  | NFIL3 | 0 | 75.1714666 | 0 | 34.7508496 | 40.0799725 | 57.9793682 |
|  | PHTF2 | 0 | 0 | 0 | 0 | 0 | 9.11028916 |
|  | PLAGL1 | 43.8261784 | 45.5656847 | 0 | 5.10246474 | 3.9075599 | 0 |
|  | PRDM1 | 407.539933 | 183.829985 | 27.6094825 | 88.6871843 | 205.111054 | 492.461859 |
|  | TOX | 161.594493 | 153.060086 | 89.90177 | 63.321099 | 51.7388551 | 49.9434509 |
|  | YBX3 | 111.190195 | 212.454035 | 150.895747 | 165.922737 | 176.35872 | 209.056716 |
|  | ZBED5 |  |  |  |  |  |  |
| downregulated TFs in int | AR | 35.5426542 | 46.8528725 | 34.7129004 | 30.2443319 | 39.1323278 | 53.317403 |
|  | ARID5A | 18.446781 | 8.10692377 | 79.8361361 | 137.45512 | 158.15867 | 79.8012702 |
|  | BACH2 | 22.0916361 | 307.55198 | 416.792284 | 381.614484 | 483.824992 | 472.709793 |
|  | FOXP1 | 0 | 0 | 0 | 0 | 1.45309553 | 0 |
|  | HLF | 154.493452 | 238.689604 | 86.038237 | 249.365064 | 237.766176 | 207.041207 |
|  | IKZF1 | 14.4050706 | 25.2605758 | 0 | 71.263307 | 88.8280628 | 22.2456706 |
|  | LEF1 | 0 | 204.397453 | 530.379613 | 380.805129 | 644.084353 | 474.01431 |
|  | SATB1 | 0 | 0 | 12.266176 | 13.2196363 | 28.463295 | 9.51655881 |
|  | SMAD1 | 47.65043 | 13.8650103 | 15.3433065 | 0 | 4.75754774 | 0 |
|  | TBX21 | 19.7441163 | 142.842389 | 125.784282 | 129.645428 | 167.796185 | 246.487583 |
|  | TCF7 | 0 | 5.57413601 | 0 | 0 | 0 | 6.32159203 |
|  | ZEB2 |  |  |  |  |  |  |

**Table S3H. The average expression levels of both upregulated TFs in VAT Treg were analyzed in six Treg clusters.**

|  | SYMBOL | spleen_tr_cluster1 | spleen_tr_cluster2 | spleen_tr_cluster3 | spleen_tr_cluster4 | spleen_tr_cluster5 | spleen_tr_cluster6 |
| --- | --- | --- | --- | --- | --- | --- | --- |
| upregulated  TFs in VAT | FOXP3 | 0 | 0 | 0 | 0 | 0 | 0 |
|  | ATF3 | 0 | 0 | 0 | 0 | 0 | 0 |
|  | PPARG | 65.819623 | 57.1931424 | 648.857949 | 36.5735657 | 50.6109906 | 0 |
|  | EGR1 | 0 | 13.7392552 | 230.77757 | 25.028742 | 3.96404047 | 0 |
|  | IRF4 | 816.809438 | 525.60076 | 475.207069 | 408.896001 | 285.237522 | 261.408924 |
|  | IKZF2 | 19.9592831 | 4.21727395 | 1380.27461 | 44.5596905 | 1.91478443 | 4.4769571 |
|  | NR4A1 | 0 | 9.06451465 | 0 | 37.0030716 | 41.0876967 | 80.1347806 |
|  | SP6 | 236.834553 | 160.98833 | 425.540187 | 297.561481 | 205.80146 | 164.829394 |
|  | GATA3 | 0 | 5.46251079 | 0 | 0 | 0 | 0 |
|  | FOSL2 | 0 | 0 | 0 | 0 | 2.89414763 | 0 |
|  | RORA | 1050.37204 | 584.946206 | 1051.09254 | 466.534548 | 390.428512 | 385.243463 |
|  | NFKBIA | 0 | 0 | 0 | 0 | 0 | 0 |
|  | NR4A3 | 0 | 45.174212 | 106.345645 | 42.2767741 | 49.7320281 | 147.327969 |
|  | CSRNP1 | 0 | 6.44246875 | 33.0317378 | 40.5842261 | 36.5683317 | 13.1589268 |
|  | NFKBIZ | 0 | 0 | 0 | 0 | 0 | 0 |
|  | KLF8 | 0 | 34.7266527 | 0 | 0 | 0 | 0 |
|  | NFIL3 | 38.7912642 | 0 | 33.2724862 | 3.14151258 | 2.39740888 | 0 |
|  | FOS | 32.2934832 | 0 | 0 | 7.02622186 | 4.04279079 | 0 |
|  | NR1D1 | 0 | 4.19382166 | 0 | 0 | 18.2901712 | 0 |
|  | KLF4 | 36.6796332 | 30.5651284 | 566.400221 | 20.82966 | 9.34835104 | 32.9310435 |
|  | NFKBID | 43.8261784 | 45.5656847 | 0 | 5.10246474 | 3.9075599 | 0 |
|  | PRDM1 | 0 | 30.8276288 | 0 | 0 | 12.4525764 | 0 |
|  | ARNT2 | 102.639921 | 60.3365575 | 138.409221 | 97.1652458 | 71.4244416 | 23.0439891 |
|  | HOPX | 0 | 0 | 0 | 0 | 0 | 0 |
|  | TOX2 | 0 | 0 | 0 | 0 | 1.45309553 | 0 |
|  | HLF | 19.7441163 | 9.96205947 | 30.9575982 | 14.3330198 | 18.44494 | 0 |
|  | CREM | 0 | 5.57413601 | 0 | 0 | 0 | 6.32159203 |
|  | ZEB2 | 0 | 0 | 0 | 0 | 0 | 0 |
|  | NPAS2 | 40.8296587 | 1.74904677 | 0 | 8.91863222 | 8.82089149 | 0 |
|  | IRF5 | 30.1659125 | 29.7353093 | 0 | 15.240853 | 6.05907595 | 0 |
|  | ATF6 | 0 | 11.3808369 | 0 | 0 | 0 | 0 |
|  | NR4A2 | 0 | 0 | 0 | 0 | 2.62178176 | 0 |
|  | HSF2 | 521.38853 | 572.049185 | 520.858646 | 576.404387 | 534.569612 | 451.792002 |
|  | JUND | 28.8101412 | 33.7606401 | 18.1077411 | 2.93040293 | 30.3720604 | 0 |
|  | KLF10 | 463.9988 | 167.275272 | 375.104452 | 201.585969 | 185.147575 | 115.385314 |
|  | JUNB | 0 | 4.19382166 | 0 | 3.75694706 | 22.5716671 | 27.7909609 |
|  | JUN | 0 | 75.1714666 | 0 | 34.7508496 | 40.0799725 | 57.9793682 |
|  | PHTF2 | 0 | 0 | 21.8221495 | 0 | 4.45378295 | 8.02536014 |
|  | THRA | 27.1301706 | 0 | 85.3181657 | 8.53515417 | 29.8305114 | 0 |
|  | TGIF1 | 22.0916361 | 23.060871 | 23.0946882 | 0 | 3.2141434 | 0 |
|  | ZBTB38 | 0 | 11.4982178 | 0 | 0 | 1.33543085 | 0 |
|  | JDP2 | 23.0556752 | 40.9092274 | 60.4229607 | 31.4625454 | 30.690186 | 56.8385074 |
|  | RBPJ |  |  |  |  |  |  |
| downregulated  TFs in VAT | TCF7 | 0 | 204.397453 | 530.379613 | 380.805129 | 644.084353 | 474.01431 |
|  | SATB1 | 47.65043 | 13.8650103 | 15.3433065 | 0 | 4.75754774 | 0 |
|  | TBX21 | 18.446781 | 8.10692377 | 79.8361361 | 137.45512 | 158.15867 | 79.8012702 |
|  | BACH2 | 0 | 0 | 0 | 2.03048984 | 0 | 0 |
|  | EOMES | 14.4050706 | 25.2605758 | 0 | 71.263307 | 88.8280628 | 22.2456706 |
|  | LEF1 | 31.9467945 | 170.325939 | 22.9357798 | 59.9675766 | 113.487279 | 64.4001994 |
|  | KLF3 | 154.493452 | 238.689604 | 86.038237 | 249.365064 | 237.766176 | 207.041207 |
|  | IKZF1 | 0 | 8.43454791 | 0 | 0 | 21.0186699 | 4.4769571 |
|  | BHLHE40 | 35.5426542 | 46.8528725 | 34.7129004 | 30.2443319 | 39.1323278 | 53.317403 |
|  | ARID5A |  |  |  |  |  |  |
