# Supplementary figures and images for: Tissue Treg Secretomes and Transcription Factors Shared With Stem Cells Contribute to a Treg Niche to Maintain Treg-Ness With 80% Innate Immune Pathways, and Functions of Immunosuppression and Tissue Repair
Source: Front Immunol. 2021 Feb 5;11:632239. doi: 10.3389/fimmu.2020.632239 (PMC7892453; doi:10.3389/fimmu.2020.632239)

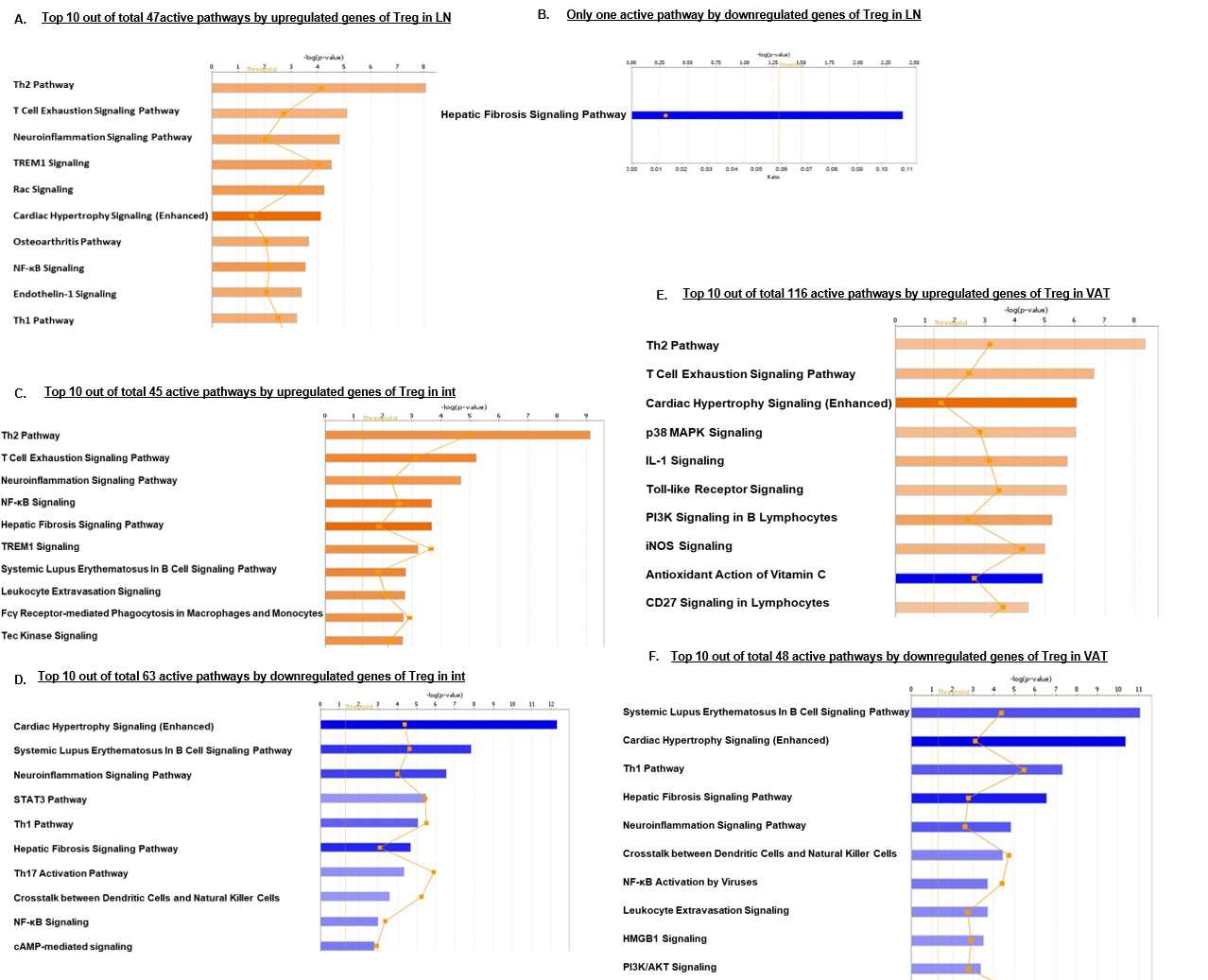

Supplement: Supplementary Figure 1 — Ingenuity Pathway Analysis also showed expression changes of upregulated and downregulated genes of Treg vs. Tconv from different tiers of four tissues we studied were involved in canonical pathways to maintain their roles of homeostasis, the results of active pathways also confirmed the tiers of these four tissues we defined (cutoff: z-score>2). [file Image_1.tif]

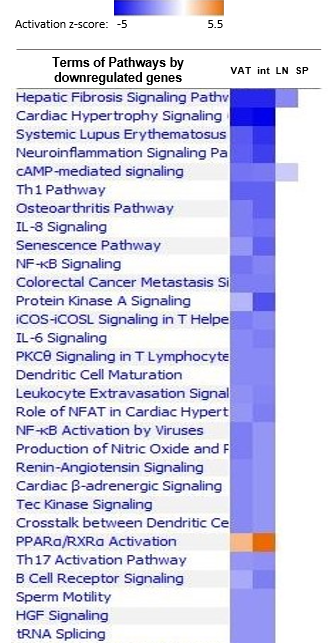

Supplement: Supplementary Figure 2 — Ingenuity Pathway Analysis of comparison analysis among all the four tissues showed common top 10 pathways by downregulated genes of Treg also indicate with the tier changed, efficiency of the common pathways could be changed responsively. [file Image_2.tif]

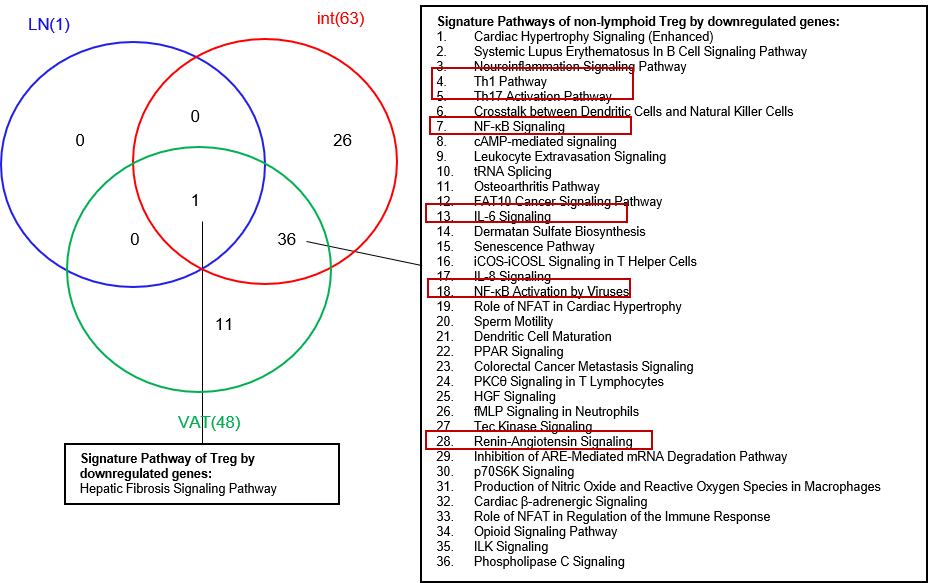

Supplement: Supplementary Figure 3 — Venn Diagram showed that the Hepatic Fibrosis Signaling Pathyway was shared by downregulated genes of Treg in LN, int, and VAT compared to Tconv, indicating an important function of Treg during hepatic fibrosis which we identified in our latter data. Thirty-six pathways were shared by that of Treg in peripheral tissues int and VAT, indicating their function could be amplified in peripheral non-lymphoid tissues rather that lymphoid tissues. [file Image_3.tif]

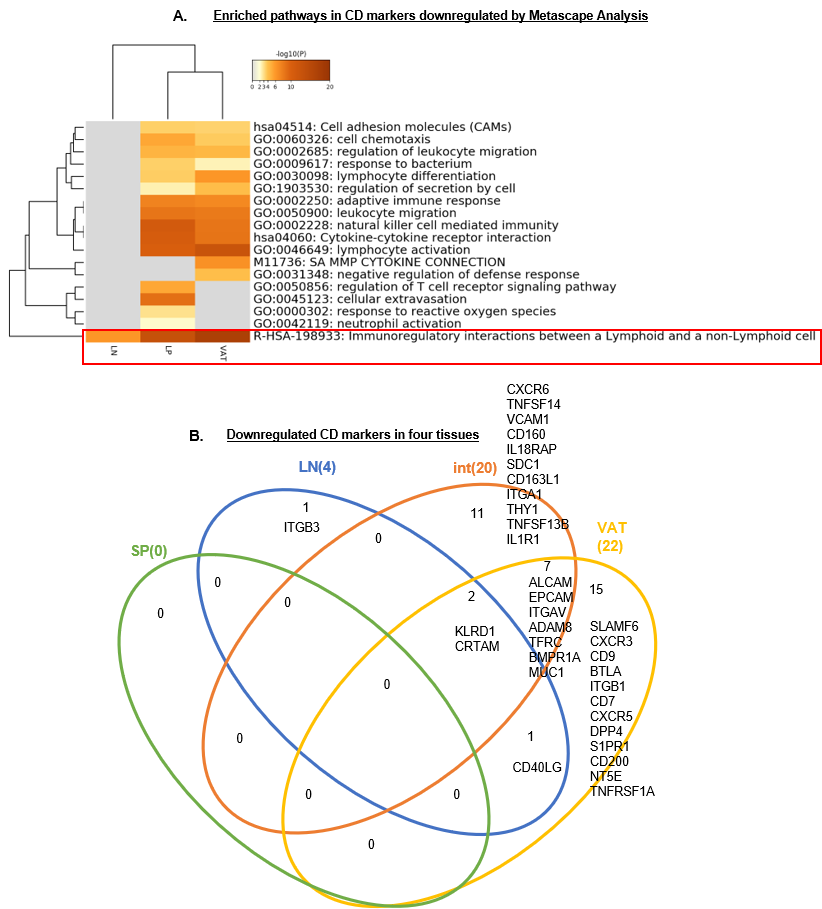

Supplement: Supplementary Figure 4 — The expression changes of total 373 CD markers (https://www.proteinatlas.org/search/protein_class:CD+markers) shared (logFC) in four tissue Treg indicated the modulation and plasticity of Treg in tissues. The Metascape analysis showed that cytokine-cytokine receptor interactions, TNFs binding their physiological receptors, and cytokine production were all activated by upregulated CD markers, showing important functions of cytokines and TNF receptors of Treg; and Immunoregulatory interactions between a lymphoid and a non-lymphoid cell were downregulated in Treg. [file Image_4.tif]

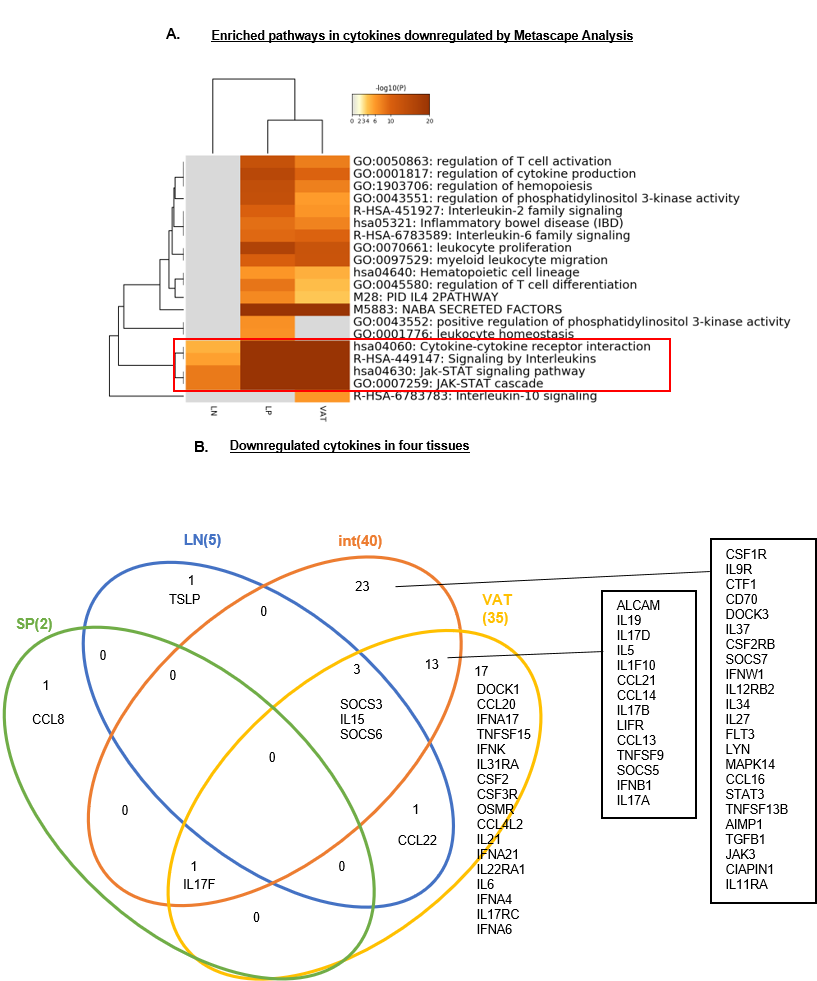

Supplement: Supplementary Figure 5 — Summary of expression changes of total 1176 cytokines and their interactors (https://www.proteinatlas.org/search/cytokine) (logFC) in four tissues we studied indicated important influence of Treg in cytokine production and modulation of tissue specific microenvironment. Enriched pathway analysis by metascape showed that JAT-STAT signaling pathway and cytokine-cytokine receptor interaction were both modulated by up- and downregulated cytokines in Treg. One interesting finding was that although we have indentified IL2 may be the upstream regulator of Treg modulation and its receptor IL2RB could induce this modulation, IL2 was downregulated in Treg from int and VAT, which means a possibility that other interactors of IL2RB participate this process. As IL2RB could interacts with Jak1 and RACK-1 according to PUBMED gene database (https://www-ncbi-nlm-nih-gov.libproxy.temple.edu/gene/3560) and metascape analysis also showed the important role of Jak-STAT signaling pathway, Jak-STAT may be a significant pathway in the modulation process of Treg, especially in non-lymphoid tissues such as int and VAT. [file Image_5.tif]

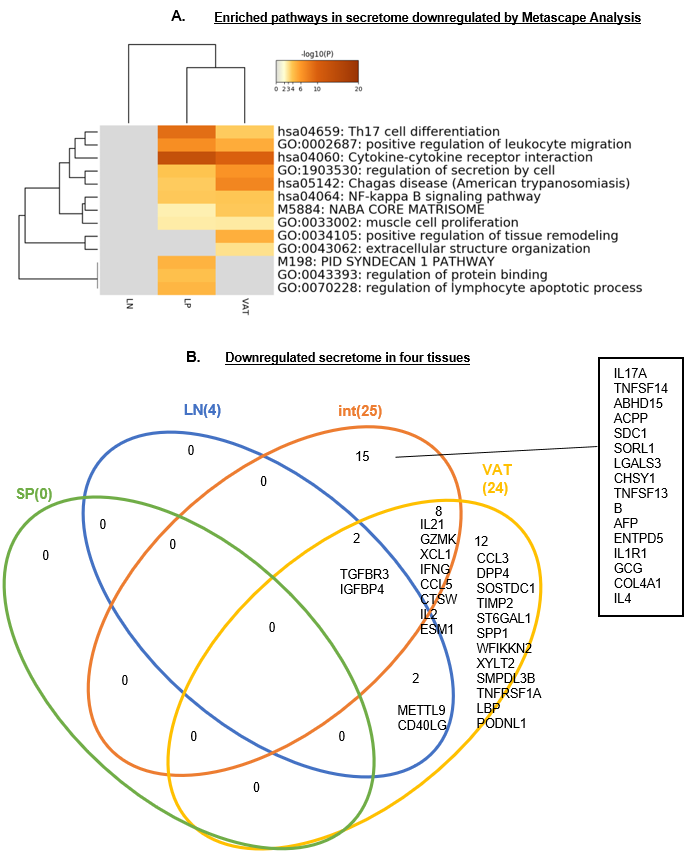

Supplement: Supplementary Figure 6 — The expression changes of total 1,706 secretome shared (logFC) in four tissue Treg indicated that secretomic changes of Treg in different tissue Treg could mediate the regulation of leukocyte activation, cytokine production, and regulation of immune effector process by upregulated secretomic genes in four tissue Treg. [file Image_6.tif]

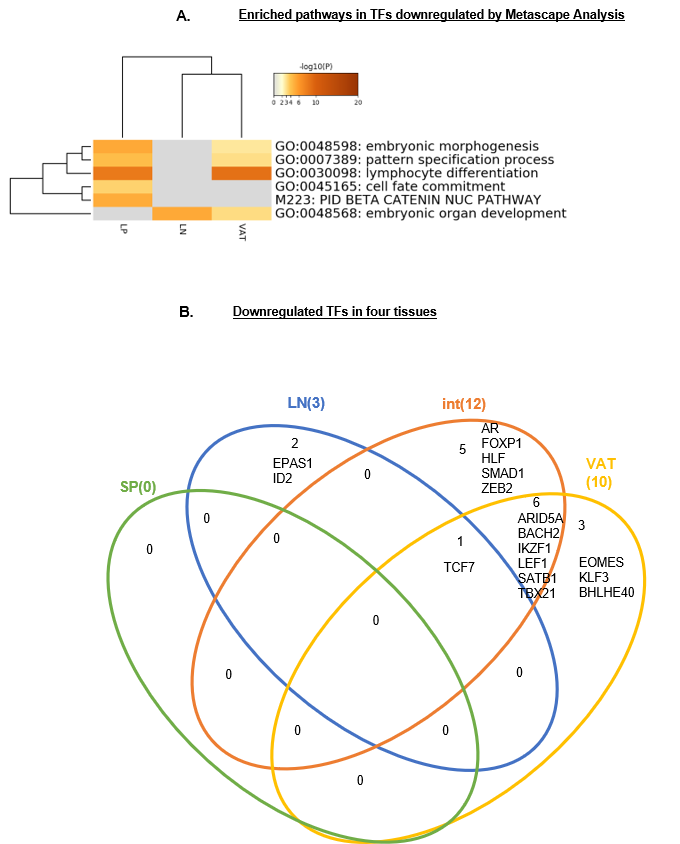

Supplement: Supplementary Figure 7 — Expression changes of total of 1,496 transcription factors (TFs) were analyzed in our studys. The original gene lists were all obtained according to the leading program the Human Protein Atlas(HPA, https://www.proteinatlas.org/). [file Image_7.tif]

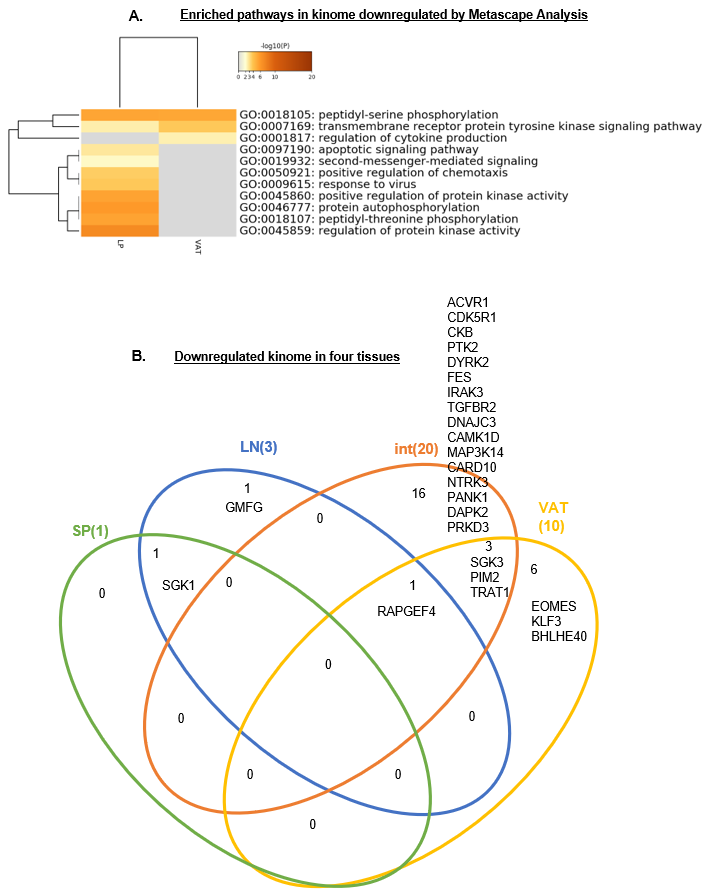

Supplement: Supplementary Figure 8 — The expression changes of kinome (all the kinases encoded by human genome) were identified in four tissue Treg. [file Image_8.tif]

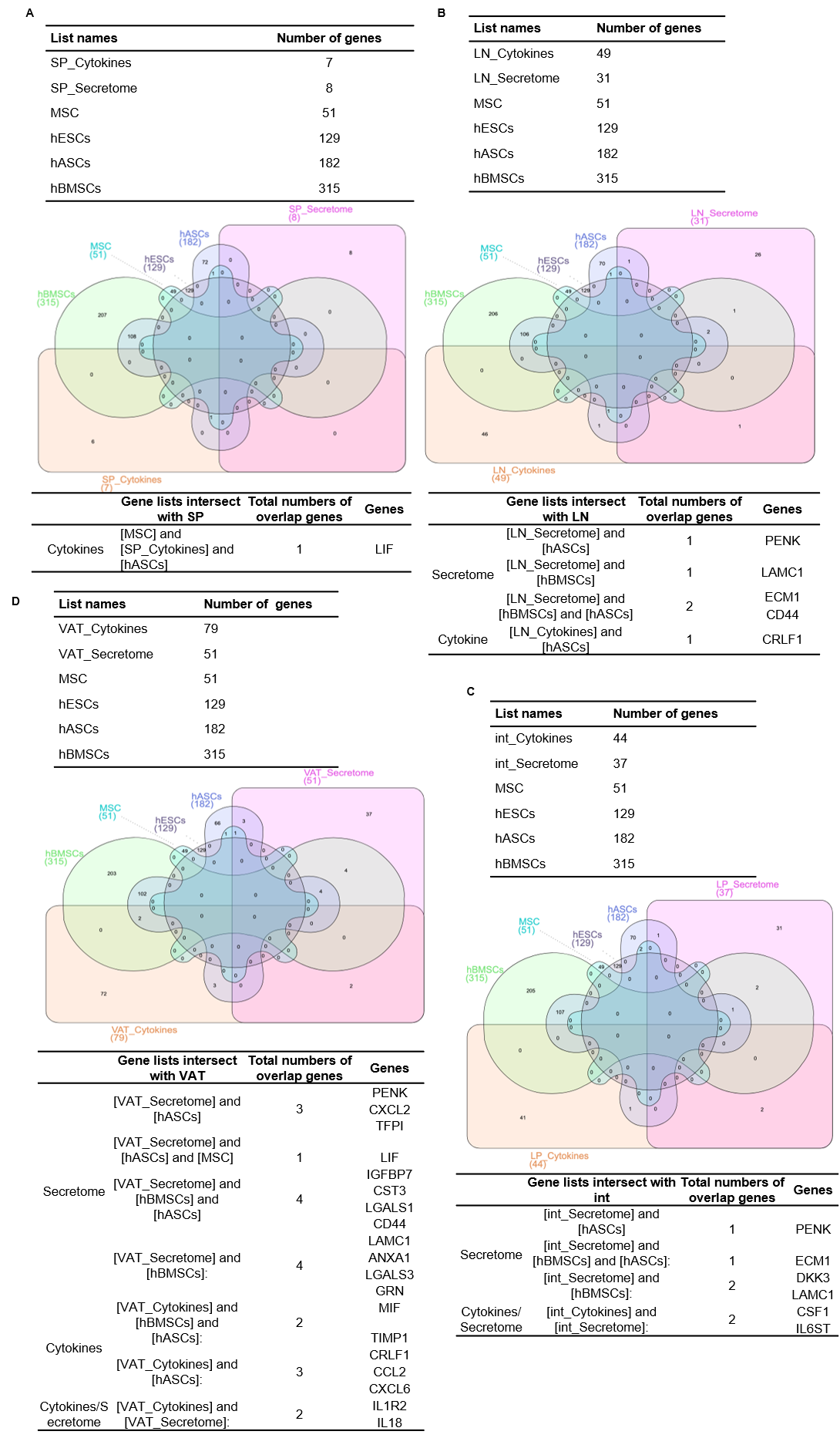

Supplement: Supplementary Figure 9A — The cytokine and secretomic genes shared between splenic Treg and four types of stem cells were identified (hESCs, hBMSCs, hASCs, MSCs), which were illustrated by Venn diagram. [file Image_9.tif]

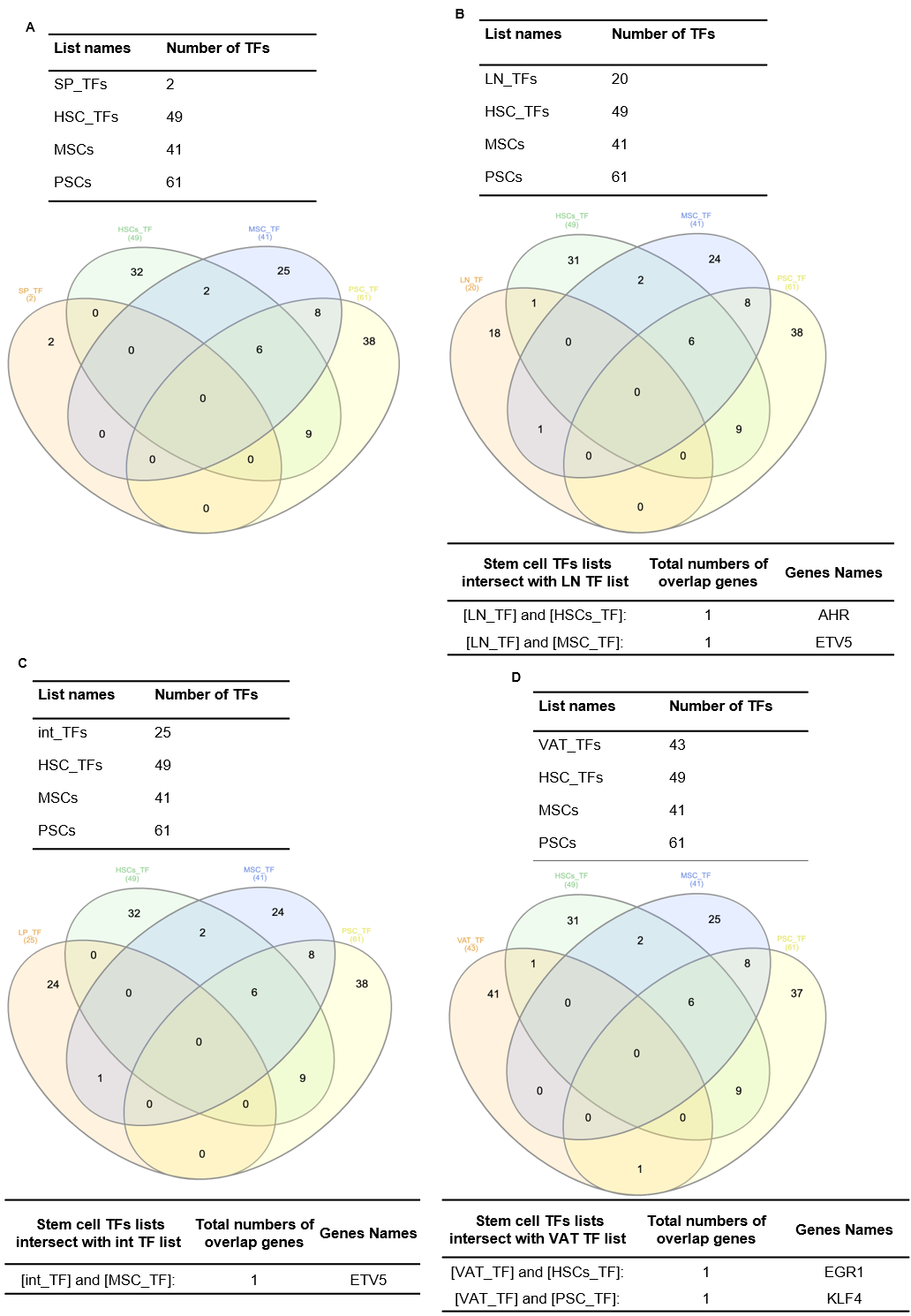

Supplement: Supplementary Figure 10A — Transcription factors (TFs) of Tregs in spleen overlap with three types of stem cells (HSC_TF, MSC_TF, and PSC_TF). There is no overlapped TFs in spleen among other three stem cell groups. [file Image_10.tif]
